# Global prevalence and disability-adjusted life years of hypertensive heart disease: A trend analysis from the Global Burden of Disease Study 2019

**DOI:** 10.7189/jogh.14.04172

**Published:** 2024-08-30

**Authors:** An-Bang Liu, Yan-Xia Lin, Ting-Ting Meng, Peng Tian, Jian-Lin Chen, Xin-He Zhang, Wei-Hong Xu, Yu Zhang, Dan Zhang, Yan Zheng, Guo-Hai Su

**Affiliations:** 1 Shandong First Medical University and Shandong Academy of Medical Sciences, Jinan, Shandong, China; 2 Department of Cardiology, Central Hospital Affiliated to Shandong First Medical University, Jinan, Shandong, China; 3 Research Center of Translational Medicine, Central Hospital Affiliated to Shandong First Medical University, Jinan, Shandong, China; 4 Jinan Central Hospital, Shandong University, Jinan, Shandong, China; 5 School of Clinical Medicine, Shandong Second Medical University, Weifang, Shandong, China

## Abstract

**Background:**

As hypertensive heart disease (HHD) presents a significant public health challenge globally, we analysed its global, regional, and national burdens and trends from 1990 to 2019.

**Methods:**

We used data from the Global Burden of Disease (GBD) 2019 study, focussing on the age-standardised prevalence rates (ASPRs) of HHD prevalence, age-standardised disability-adjusted life year (DALY) rates, average annual percentage change (AAPC), and risk factor attributions. We compared the HHD burden across sociodemographic index (SDI) strata, gender, age groups, and 204 countries and territories.

**Results:**

In 2019, the global prevalence of HHD was estimated at 18 598 thousand cases, with DALYs reaching 21 508 thousand. From 1990 to 2019, the ASPRs increased (AAPC = 0.21; 95% confidence interval (CI) = 0.17, 0.24), while the age-standardised DALY rates decreased (AAPC = −0.45; 95% CI = −1.23, −0.93). We observed the highest increase in ASPRs in high-middle SDI quantile countries, and an overall negative correlation between age-standardised DALY rates and SDI. Individuals above 70 years of age were the most affected, particularly elderly women. There has been a significant increase in HHD burden attributed to high body mass index (BMI) since 1990. The burden of HHD is concentrated in the middle SDI quintile, with population ageing and growth being major drivers for the increase in DALYs. We identified opportunities for reducing age-standardised DALY rates in the middle SDI quintile or lower.

**Conclusion:**

Despite a declining trend in the age-standardised DALY rates, the ASPRs of HHD continue to rise, especially in high-middle SDI regions. Meanwhile, countries in middle and lower SDI quintiles face a higher burden of age-standardised DALY rates. Targeted attention towards elderly women and controlling high BMI, alongside enhancing hypertension and HHD management awareness, is crucial for reducing the global burden of HHD.

Hypertension, defined as a systolic/diastolic blood pressure ≥140/90 mm Hg, is a major risk factor for cardiovascular diseases (CVDs) and the leading cause of disability-adjusted life years (DALYs) worldwide [1–3]. It affects approximately 31% of the global adult population (around 1.4 billion) and is characterised by a rising prevalence which expected to exceed 1.6 billion by 2025 [1,3].

According to prior studies, earlier age at onset of hypertension was associated with increased risk of CVD and all-cause mortality [4]. Hypertensive heart disease (HHD), a common consequences of hypertension-targeted organ damage, is characterised by left ventricular hypertrophy (with around 40% prevalence in hypertensive patients), which can lead to systolic and diastolic dysfunction [5,6]. By definition, HHD is the result of chronic hypertension, with interstitial myocardial fibrosis being the underlying cause of hypertension-induced cardiac remodelling [7–9]. Notably, patients with HHD have a higher risk of cardiovascular events and mortality than patients with hypertension alone [7–9]. Moreover, HHD may progress to serious cardiovascular complications like atrial fibrillation, coronary heart disease, stroke, heart failure, peripheral arterial disease, myocardial infarction, and sudden death [5,9–13].

Despite hypertension being a widespread chronic condition, global blood pressure control rates remain alarmingly low, at just 32.5% among the treated population [14]. Furthermore, a high body mass index (BMI) is considered to be one of the main causes of DALYs in HHD patients [15]. Moreover, excessive dietary salt intake affects endothelial function and structure, disrupts the regulation of the neural-humoral-immune system, and increases systemic peripheral resistance, which is associated with the development of hypertension and accelerates the onset of HHD [16]. HHD has also been identified as the second leading cause of heart failure after ischaemic heart disease [17,18].

Hypertension is a major global health challenge, primarily contributing to CVD and cerebrovascular disorders [19]. HHD results from chronic pressure and volume overload in hypertensive individuals, causing increased afterload, myocardial wall stress, and fibroblast proliferation, leading to left ventricular hypertrophy, the principal pathological marker of HHD [13,20]. Notably, left ventricular hypertrophy elevates CVD risk and mortality, independent of blood pressure [13]. Consequently, HHD emerges as a critical public health concern worthy of prioritisation and attention. However, HHD epidemiology varies globally due to geographic and demographic factors, and prior studies did not assess trends or were focussed on specific regions [21–23]. Updated analyses are needed to understand the epidemiological characteristics of HHD in different countries and populations to address the emerging challenges.

We used data from the Global Burden of Disease (GBD) 2019 study to assess the prevalence and DALYs of HHD from 1990 to 2019, stratified by location, age, sex, and sociodemographic index (SDI). In doing so, we aimed to provide a detailed, up-to-date epidemiological analysis of HHD worldwide and help public health managers make informed, evidence-based decisions regarding the prevention and management of HHD.

## METHODS

### Data sources

We extracted the data on the HHD burden from the GBD 2019 database [24], which is managed by the Institute for Health Metrics and Evaluation. It includes data on the disease burden at the global, regional, and national levels from 1990 to 2019, which can be queried via the Global Health Data Exchange. Details and methods used in the GBD 2019 study have been published elsewhere [25,26]. The GBD 2019 study adhered to the Guidelines for Accurate and Transparent Reporting of Health Estimates (GATHER) [27]. The GBD 2019 research team used DisMod-MR, version 2.1 (Institute for Health Metrics and Evaluation, University of Washington, Seattle, WA, USA) [25], a Bayesian meta-regression modelling tool, to estimate the prevalence and burden of HHD, while they calculated 95% uncertainty intervals (UI) using the 25th and 95th values from 1000 draws of the posterior distributions [28]. As we used publicly available data from the GBD 2019 in our analyses, we did not require specific ethical approval.

### Definition

HHD is classified under ICD-9 codes 402.0–402.91 and ICD-10 codes I11.0–I11.9 [25] (see Methods section in the **Online Supplementary Document**). We retrieved data on the burden of HHD from vital registries, hospitals, and physician visit data, which can be downloaded from the GBD 2019 Data Entry Source Tool website [25,29].

We reported on the prevalence, disability-adjusted life years (DALYs), and their 95% UIs for HHD worldwide, across 21 regions and in 204 countries from 1990 to 2019 (see Methods in the **Online Supplementary Document**). We used DALYs for our analyses as they serve as an indicator of the disease burden, determined by the sum of years lived with disability and the years of life lost. We otherwise utilised age-standardised rates to assess and compare burdens in countries or regions with different age structures.

We further used SDI, an indicator reflecting the development status of a country or region, stratified into five levels corresponding to the quintiles of the SDI (low, low-middle, middle, high-middle, and high). The SDI is a combined measure of the total fertility rate of individuals below 25 years old, the educational attainment of individuals aged 15 and above, and lagging per capita income distribution in a given area. The SDI ranges from 0 to 1, where 0 signifies the lowest possible level of developmental status associated with a health outcome while 1 signifies the highest [25].

### Risk factors

GBD 2019 provided an assessment of attributable DALYs and mortality for 87 risk factors at global and regional levels [30]. The risk factors for HHD include a high BMI, alcohol consumption, diet high in sodium, lead exposure, and low and high temperature. Since the percentage of DALYs attributable to high systolic blood pressure was 100%, we reported the percentage of other risk factors. Details of the definitions of these risk factors and methods for quantifying the percentage contribution to DALYs have been explained elsewhere [21–23], while the relevant risk factors for HHD DALYs are available in the GBD Outcomes Tool [24].

### Statistical analyses

The methodology of GBD 2019 has been detailed elsewhere [25,26]. Here we quantified the global burden of HHD using prevalence rates and DALYs, as well as age-standardised rates and average estimated annual percentage change (AAPC). The age-standardised rate reported per 100 000 population compares disease burdens across varying age structures, while the AAPC and its 95% confidence interval (CI) acts as a combined measure reflecting patterns of age-standardised rates over time. Specifically, it is the weighted average of the annual percentage change which indicates the overall annual changes (increases, decreases, or stability), whereby an AAPC of 0.01 denotes a 1% annual increase. We used the joinpoint regression programme, version 4.9.0.0 (Surveillance Research Program, National Cancer Institute, National Institute of Health, Bethesda, MD, USA) for a 30-year trend analysis of the HHD disease burden by employing connected-point regression for the AAPC. This programme, by default, fits the simplest model to the data, allowing up to five connectors for a six-segment final model. A smoothed spline model demonstrated the changing age-standardised prevalence rates (ASPRs), age-standardised DALY rates, and SDI relationship from 1990–2019 globally and across 21 regions, using the Pearson correlation coefficient to quantify the correlation.

We further used decomposition analysis to describe the factors linked to changes in DALYs from 1990 to 2019. The overall difference in DALYs consists of three elements: ageing, population growth, and epidemiologic changes (age-specific rates of DALYs, which combine potential age- and population-adjusted mortality and morbidity). To evaluate the correlation between DALYs burden and SDI, we employed frontier analysis to identify effective differences among 204 countries in terms of the minimum attainable burden determined by their developmental status. Frontier analysis refers to the assessment of potentially achievable age-standardised DALY rates based on developmental status as measured by the SDI; this allows us to better quantify the maximum level of DALYs that can be achieved across the developmental spectrum. For example, the greater a country's effective difference distance from the frontier line, the greater room for improvement in DALYs should be possible for that country or region based on its position on the development spectrum [31,32] (Methods in the **Online Supplementary Document**).

We analysed and visualised all data in R, version 4.2.1 (R Core Team, Vienna, Austria). *P*-values <0.05 denoted statistical significance.

## RESULTS

### Prevalence and changes in hypertensive heart disease

**Figure 1:**
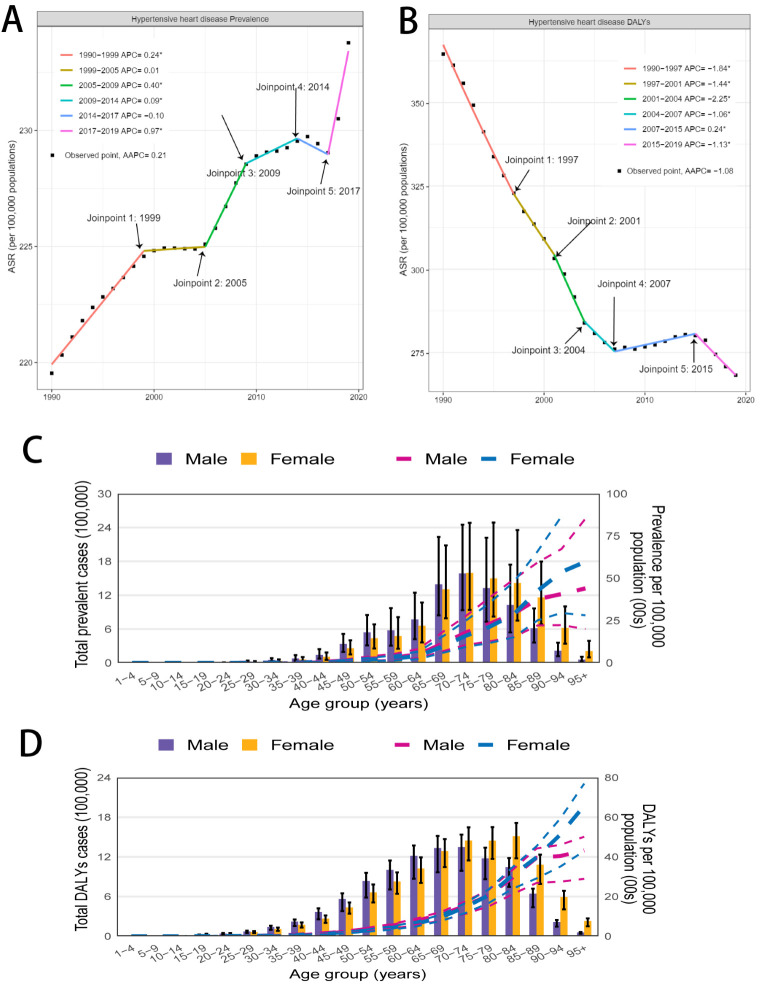
Global changes in age-standardised rates of HHD from 1990 to 2019. **Panel A.** Global changes of ASPRs. **Panel B.** Global changes of age-standardised DALY rates. **Panel C.** Age-specific cases and rates of prevalence of HHD by sex in 2019. **Panel D.** Age-specific cases and rates of DALYs. DALY – disability-adjusted life year.

There were 18 598 HHD cases (in thousands) in 2019 globally, with an age-standardised rate of 233.8 per 100 000 population – a 6.5% increase from 1990. We observed the highest increases in this period in high-middle SDI countries (14.6%; 95% UI = 9.8, 20.1) (Table S1 in the **Online Supplementary Document**). The global ASPRs for HHD similarly increased between 1990 and 2019 (AAPC = 0.21; 95% CI = 0.17, 0.24), with varying trends: a slow increase from 1999–2005 and 2009–14, a decline from 2014–17, and a significant rise after 2017 (**Figure 1**, Panel A). Despite a decreasing ASPR in the middle SDI quintile (AAPC = −0.17; 95% CI = −0.20, −0.13), it still had the highest burden from 1990 to 2019 (Figure S1, Panel C in the **Online Supplementary Document**).

**Figure 2:**
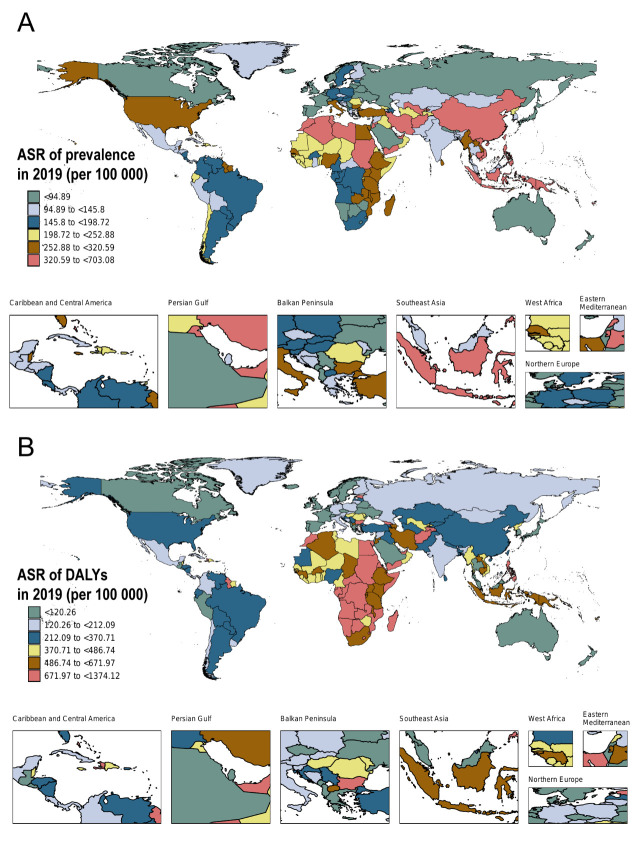
Age-standardised rates of HHD for both sexes in 204 countries and territories, 2019. **Panel A.** Age-standardised prevalence. **Panel B.** Age-standardised DALYs. DALY – disability-adjusted life year.

Looking at specific regions in 2019, East Asia had the highest ASPR (426.1 per 100 000 population; 95% UI = 306.6, 574.8), while Eastern Europe had the lowest (56.5 per 100 000 population; 95% UI = 38.2, 83) (Table S1 in the **Online Supplementary Document**). At the national level, Ukraine and the Cook Islands recorded the lowest (11.9 per 100 000 population; 95% UI = 8, 17.3) and highest (703.1 per 100 000 population; 95% UI = 532.9, 920.7) ASPRs, respectively (Table S3 in the **Online Supplementary Document**). East Asia, Southeast Asia, Oceania, North Africa and the Middle East had the high ASPR burden for HHD, while East Asia and Southeast Asia were the two most significant regions in terms of all-age rates of prevalence (**Figure 2**, Panel A; Figure S2, Panel A in the **Online Supplementary Document**).

**Figure 3:**
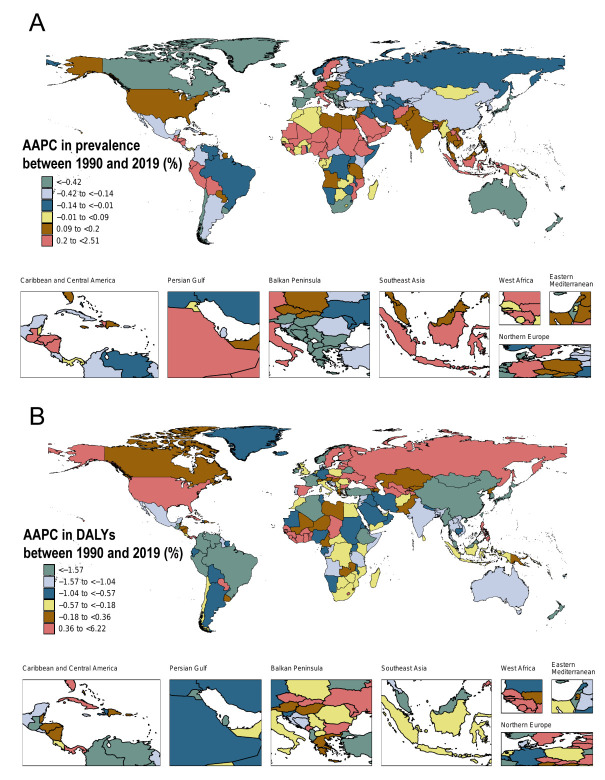
AAPC of age-standardised rates of HHD for both sexes in 204 countries and territories from 1990 to 2019. **Panel A.** AAPC of age-standardised prevalence. **Panel B.** AAPC of age-standardised DALYs. DALY – disability-adjusted life year.

Bolivia (AAPC = 2.51), Italy (AAPC = 1.35), and Cameroon (AAPC = 1.15) experienced significant increases in the prevalence of HDD from 1990 to 2019. Conversely, France (AAPC = −1.66), Croatia (AAPC = −1.66), and Serbia (AAPC = −1.78) saw the largest decreases (**Figure 3**, Panel A and Table S3 in the **Online Supplementary Document**), aligning with percentage changes in the ASPR (Figure S2, Panel C in the **Online Supplementary Document**).

### DALYs and changes in hypertensive heart disease

In 2019, the global burden of HHD DALYs was 21 508 thousand, with an age-standardised rate of 268.2 per 100 000, declining 26.4% since 1990. The steepest decrease occurred in the middle SDI quintile (−41.1%; 95% UI = −49.2, −30.9) (Table S2 in the **Online Supplementary Document**). Furthermore, the global age-standardised DALY rate for HHD fell from 1990 to 2019 (AAPC = −1.08; 95% CI = −1.23, −0.93), with a slow increase from 2007 to 2015 and a significant drop thereafter (**Figure 1**, Panel B), particularly in the middle SDI quintile (Figure S1, Panel D in the **Online Supplementary Document**).

Regionally in 2019, central sub-Saharan Africa had the highest age-standardised DALY rate (970.3 per 100 000 population; 95% UI = 625.8, 1310.4) while Australasia had the lowest (38.9 per 100 000 population; 95% UI = 32.9, 46.6) (Table S2 in the **Online Supplementary Document**). At the national level, Israel had the lowest highest age-standardised DALY rate in 2019 (27.8 per 100 000 population; 95% UI = 21.8, 63.6), while Afghanistan had the highest (1374.1 per 100 000 population; 95% UI = 467.2, 2020.7) (Table S4 in the **Online Supplementary Document**). Most countries in Africa, Oceania, and Southeast Asia had a high age-standardised DALY rate burden for HHD, while all-age rates of DALYs showed heterogeneity in different regions of the world (**Figure 2**, Panel B; Figure S2, Panel B in the **Online Supplementary Document**).

Estonia (AAPC = 6.22), Latvia (AAPC = 5.72), and the Republic of Moldova (AAPC = 5.32) had the most notable increases in their age-standardised DALY rates from 1990 to 2019. Conversely, Colombia (AAPC = −3.78), the Republic of Korea (AAPC = −4.31), and Israel (AAPC = −4.89) saw significant decreases (**Figure 3**, Panel B and Table S4 in the **Online Supplementary Document**), echoed in the percentage changes in age-standardised DALY rates (Figure S2, Panel D in the **Online Supplementary Document**).

### Age and sex patterns

The prevalence cases of HHD peaked in the age group of 70–74 for both sexes and declined thereafter. The prevalence cases of HHD were higher in men aged 65–79 years and women aged 70–84 years (**Figure 1**, Panel C). The number of DALYs due to HHD peaked in the 70–74-year-old group for men and the 80–84-year-old group for women, while the number of DALYs was higher for men than for women in all age groups <70 years (**Figure 1**, Panel D). Furthermore, we observed that the age-specific prevalence and DALY rates of HHD increased with age. However, the rate of DALYs in men plateaued in 85–89-year-old age group. From 1990 to 2019, the ASPRs (Figure S1, Panel A in the **Online Supplementary Document**) indicated that, although men had higher prevalence, the changes were relatively stable, while women experienced a significant increase. The age-standardised DALY rates (Figure S1, Panel B in the **Online Supplementary Document**) suggested that the burden for both sexes was decreasing, but there was an increasing trend for men's burden from 2007 to 2015. Furthermore, age-stratified trends in crude rates of prevalence and DALYs (Figure S1, Panels E and F in the **Online Supplementary Document**) from 1990 to 2019 indicated that the individuals over the age of 70 bear the highest burden of HHD.

### Association with the sociodemographic index

Regionally from 1990 to 2019, we observed an inverted U-shaped relationship between the SDI and the ASPR. This means that as SDI rose initially, so did the prevalence, peaking around 0.45, then plateauing between 0.55 and 0.65 and declining again. The overall correlation between ASPR and SDI was r = 0.36 (95% UI = −0.42, −0.29). Eastern sub-Saharan Africa (4.6%; 95% UI = 1.6, 7.6), Oceania (1.1%; 95% UI = −4.8, 7.8), North Africa and the Middle East (2.7%; 95% UI = −2.2, 8.3), Southeast Asia (1.5%; 95% UI = −0.6, 3.5), and East Asia (−5.9%; 95% UI = −8.6, −2.8) had higher than expected burdens in ASPR from 1990 to 2019 (Table S1 and Figure S3, Panel A in the **Online Supplementary Document**).

There was a negative linear association between SDI and the age-standardised DALY rates for HHD during the same period. As SDI increased, the age-standardised DALY rates decreased, with a correlation of r = −0.73 (95% UI = −0.77, −0.7). Eastern sub-Saharan Africa (−31.2%; 95% UI = −43.4, −9.5), Central sub-Saharan Africa (−15.1%; 95% UI = −33.3, 7.3), Oceania (−8.8%; 95% UI = −25.9, 13.6), North Africa and the Middle East (−25%; 95% UI = −42, −7.4), and southern sub-Saharan Africa (−4.7%; 95% UI = −17.5, 5.9), had higher than expected burdens in age-standardised DALY rates from 1990 to 2019 (Table S2 and Figure S3, Panel B in the **Online Supplementary Document**).

Additionally, we visualised the prevalence and DALY rates against SDI for 204 regions in 1990 and 2019 and found that age-standardised rates of both prevalence and DALYs generally decreased with higher SDI (Figure S4 in the **Online Supplementary Document**).

### Risk factors

The proportion of HHD DALYs attributable to individual risk factors (except high systolic blood pressure) varied across GBD regions. Globally in 1990, high BMI (25.5%), high sodium diet (20.8%), and lead exposure (9.3%) contributed most to HHD DALYs (Figure S5, Panel A in the **Online Supplementary Document**). The highest contributors in 2019, in turn, were high BMI (40.5%), high sodium diet (16.3%), and alcohol use (9.5%) (Figure S5, Panel B in the **Online Supplementary Document**). In particular, high BMI increased from 1990 to 2019 in all 21 regional levels, while other risk factors showed slight differences in the increase or decrease in changes (Figure S5 in the **Online Supplementary Document**).

### Decomposition analysis in DALYs

**Figure 4:**
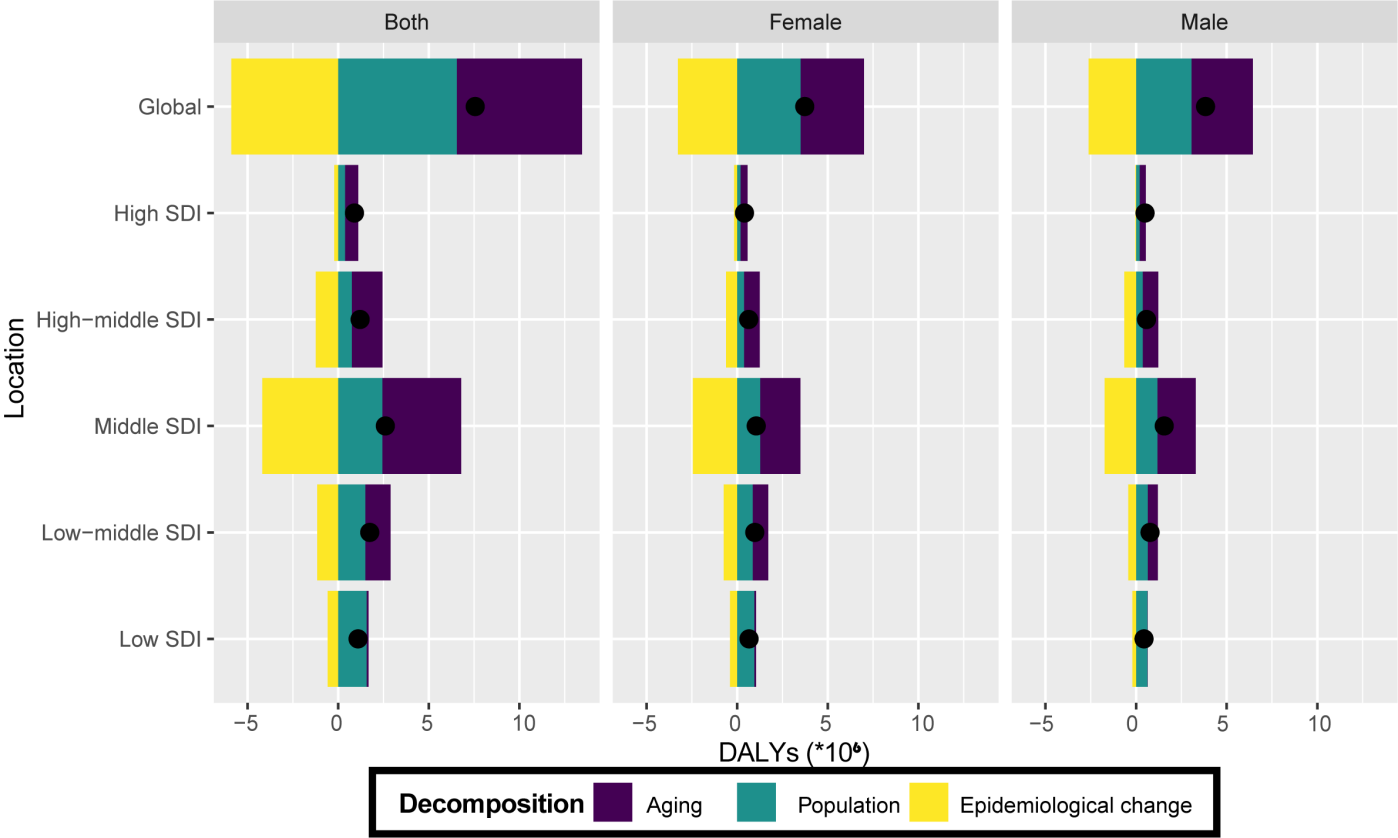
Changes in HHD DALYs of both sexes, women, and men according to population-level determinants of ageing, population growth, and epidemiological change from 1990 to 2019 at the global level and by SDI quintile. The black dot represents the overall value of change contributed by all three components. For each component, the magnitude of a positive value indicates a corresponding increase in HHD DALYs attributed to the component; the magnitude of a negative value indicates a corresponding decrease in HHD DALYs attributed to the related component. DALY – disability-adjusted life year, SDI – sociodemographic index.

From 1990 to 2019, there has been a significant increase in DALYs numbers of HHD worldwide from 1990 (13 943.6 (in thousands)) to 2019 (21 508 (in thousands)), with the highest increase observed in the middle SDI quintile. Men and women performed similarly in this increase (**Figure 4** and Table S2 in the **Online Supplementary Document**). Ageing was the primary contributor to the global HHD burden (91.27%), followed by population growth (86.59%) and epidemiological changes (−77.85%). The impact of ageing was most pronounced in the middle SDI quintile (166.99%), decreasing progressively in the high-middle (140.12%), high (81.03%), low-middle (80.28%), and low SDI quintiles (9.25%). The surge in HHD DALYs in low SDI quintile was largely driven by population growth (143.42%). From 1990 to 2019, both globally and in five SDI quintiles, the epidemiology changes for DALYs showed a decline, with a maximum decrease of −160.63% for the middle SDI quintile and a minimum decrease of −23.29% for the high SDI quintile (**Figure 4** and Table S5 in the **Online Supplementary Document**). The decomposition analysis table of 204 countries or regions showed a substantial disparity in the contributions of the three determinants to the change in DALYs (Table S5 in the **Online Supplementary Document**).

### Frontier analysis of age-standardised DALY rates

**Figure 5:**
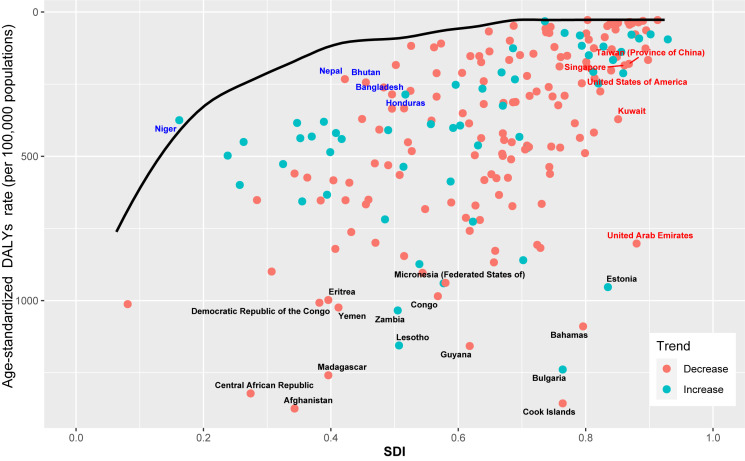
Frontier analysis of HHD in 2019 based on SDI and age-standardised DALY rate per 100 000 population. The frontier line portrayed in black, representing the potentially achievable age-standardized DALY rate based on SDI. The ‘effective difference’, or the gap between the observed points and potentially realisable age-standardised DALY rates, is measured from this black borderline. Red dots indicate a decrease in age-standardised HHD DALY rate from 1990 to 2019, while green dots indicate increase in age-standardised HHD DALY rate from 1990 to 2019. The top 15 countries with the largest effective difference (largest HHD DALYs gap from the frontier) are labelled in black. Countries and territories with low SDI (<0.5) of the top five with the lowest effective difference are labelled in blue (Niger, Nepal, Bhutan, Bangladesh, and Honduras). Countries and territories with high SDI (>0.85) of the top five with the highest effective difference are labelled in red (Taiwan (province of China), Singapore, USA, Kuwait, and United Arab Emirates).

We performed a frontier analysis using age-standardised DALY rates and SDIs to evaluate the unachieved health benefits of HHD in countries or regions with varying levels of SDI in 2019 (**Figure 5**).

Typically, countries with higher SDIs show lower effective differences, yet in our analysis, developed countries were not the only ones with a strong performance in this regard. For example, Niger, a low SDI country (SDI <0.20), showed a minimal effective difference. Conversely, higher SDI countries (SDI >0.85) such as the United Arab Emirates had a potential for greater reductions in their effective differences. We also explored the burden and effective differences in age-standardised DALY rates of HHD in 204 countries or regions with different levels of SDI in 2019 and found significant heterogeneity in the effective differences across the entire SDI spectrum (**Figure 5** and Table S6 in the **Online Supplementary Document**).

## DISCUSSION

Our findings indicate that there were approximately 18.6 million prevalent cases and 21.51 million DALYs of HHD globally in 2019. Notably, while HHD prevalence showed an upward trend, DALYs showed a downward trend, with AAPCs of 0.21 and −1.08, respectively. Prior research found that the global prevalence of HHD increased by 137.9% over the three-decade period [25]. We had similar findings, with an observed increase in HHD cases from 7.8 million in 1990 to 18.6 million in 2019. Further, we saw that the high-middle SDI regions had the most pronounced increase in their ASPR (14.6%). At the regional level, Andean Latin America had the highest surge (16.7%), with Bolivia showing the largest increase globally (103.3%).

Regions such as Eastern sub-Saharan Africa, Oceania, North Africa and the Middle East, Southeast Asia, and East Asia had higher ASPRs than other areas. This increase is likely associated with the doubling of the hypertensive population aged 30–79 years from 1990 to 2019 (from 623 million to 1.28 billion). Notably, higher hypertension prevalence rates have been observed in certain countries within Central Asia, Eastern Europe, Oceania, Southern Africa, Latin America, and the Caribbean regions [33,34]. In contrast, our results suggest that the ASPRs of HHD were the lowest in Eastern Europe, which seems to contradict the higher prevalence of hypertension reported in the region in prior studies [34,35]. Although HHD is a target organ damage of hypertension, its prevalence is not necessarily consistent with hypertension. Conversely, improvements in the treatment and control of hypertension in high-income Eastern and Central Europe from 1990 to 2019 have led to many untreated hypertensive patients in these regions being treated and their condition being controlled [34]. Yet simultaneously, the slow population growth in the region and ageing factors may explain the low ASPR of HHD despite the high prevalence of hypertension in Eastern Europe. Results from two other studies in the same period support our conclusion that the ASPR was lowest in Eastern Europe and highest in East Asia in 2019 [7,22]. Furthermore, we observed a significant increase in ASPRs between 2017 and 2019. This trend may correlate with the widespread adoption of the revised hypertension diagnostic criteria by the American Heart Association (AHA) in 2017, which lowered the systolic/diastolic blood pressure (SBP/DBP) threshold from 140/90 mm Hg to 130/80 mm Hg [36].

Despite the rising HHD prevalence, the changes of global and SDI quantiles in the age-standardised DALY rates for HHD have decreased. This indicates an improvement in the treatment and management of HHD over the past three decades. However, we observed the greatest increases in age-standardised DALY rates in Eastern Europe (59.3%), Central Asia (39.7%), and high-income North America (24.7%). As we discussed above, although Eastern Europe had the lowest ASPR of HHD in Eastern Europe in 2019, population growth, ageing, and higher prevalence of hypertension may account for the highest increase in age-standardised DALY rates for HHD in the three decades [34,35]. Additionally, we found that countries within the middle SDI had higher ASPR compared to other quintiles. Previous studies have reported similar results, with age-standardised DALYs trending upward in Eastern Europe, Central Asia, and high-income North America [7,20]. Age-standardised prevalence of HHD and temporal trends in hypertension over the past 40 years were on the rise in low- or middle-income regions such as East Asia and Central Latin America [35]. Five countries – China, India, Russia, Indonesia, and the USA – have been reported to account for more than half of the global DALYs associated with systolic blood pressure of at least 110 to 115 mm Hg [1]. The performance of these countries also somewhat supported the characterisation of the HHD burden in East Asia, Eastern Europe, Central Asia, and high-income North America in our study.

We found significantly higher ASPRs or DALYs in HHD in middle or low SDI countries than in other regions, which might be attributed to lower hypertension control rates in these contexts [37]. A prior study found that the blood pressure control rate among hypertensive patients receiving treatment in North Africa and the Middle East was 43.1% [38]. Factors contributing to lower control rates include weaker primary health care systems, limited insurance coverage, lack of affordable combination medications, and inadequate screening [38]. Some of the reasons for the low hypertension control rates of the population may include unhealthy high-sodium dietary practices, lack of physical activity, increasing prevalence of obesity, and poor patient adherence to antihypertensive medications. Additionally, health care providers often fail to follow guidelines due to the lack of financial resources, facilities, and personnel, leading to insufficient motivation to implement recommended hypertension care plans. Notably, the diagnosis of HHD in low-income countries may be underestimated, possibly due to limitations in diagnostic methods (such as electrocardiography or echocardiography) or the capacity of health systems [21,33,39]. Promoting lifestyle modifications, adhering to medical guidelines, strengthening blood pressure control, and implementing population-based care models in health care policies could contribute to improving the prevalence of HHD [19,40].

Similarly, the change in DALYs of HHD we found in our decomposition analysis was particularly pronounced in countries within the middle SDI quintile, with ageing and population growth identified as the two main drivers of DALYs change. Moreover, the prevalence and DALYs of HHD increase with age in both men and women, with older women having higher rates and numbers of DALYs compared to men. This could be associated with the loss of hormonal protection against HHD in postmenopausal women [41]. Moreover, the impact of vascular ageing (inducing hypertension) may be greater in women due to longer life expectancy, as well as lower socioeconomic status and psychological factors that they might be more prone to depression [1,42]. The effects of HHD on cardiac structure and function are similar to those of ageing [43]. With the global increase in the elderly population, the proportion of people over 60 is expected to approximately double by 2050, rising from 12% in 2015 to 22% in 2050 [43]. Therefore, effective prevention and management of HHD in the elderly population should be considered.

The ASPRs of HHD increased most significantly in the high-middle SDI regions in 1990–2019, which is consistent with previous findings reported by Qian and colleagues [22]. This may be associated with enhanced screening methods, unhealthy lifestyles such as physical inactivity, smoking, high salt diet, and increased obesity in these areas [1,44,45]. The age-standardised DALY rates for HHD in high SDI countries were lower relative to other SDI countries in 2019. This might be due to lower SDI countries allocating a smaller proportion of their gross domestic product to health care, and the lower disease awareness, coupled with insufficient screening methods [7].

The risk of HHD DALYs attributable to high BMI is significant, with increasing trends observed globally and across 21 regions. Visceral obesity increases the likelihood of chronic hypertension and the incidence of cardiovascular events [46]. A high-sodium diet is another risk factor contributing to the burden of HHD. Salt intake has a significant impact on the incidence of hypertension and the resulting prevalence of HHD, hence the need to focus on improving dietary patterns, such as the Dietary Approach to Stop Hypertension diet, recommending a daily salt intake of <5 g/d [47,48]. Alcohol consumption is also an important risk factor that cannot be overlooked in the attribution of HHD DALYs. The relationship between alcohol consumption and elevated blood pressure is dose-responsive, and important reduction in blood pressure can be observed one month after cessation of alcohol intake [49]. High BMI means obesity, more plasma volume, and the need to perfuse more tissue, resulting in increased cardiac output. Systemic circulatory renin-angiotensin-aldosterone system are also dysregulated in patients with visceral obesity. A high-calorie, high-salt diet increases angiotensin II and aldosterone, increasing blood pressure and promoting left ventricular hypertrophy leading to HHD [50,51]. Alcohol consumption may also promote increased vasoconstrictor factors and oxidative stress, leading to hypertension [52]. Risk factors like lead exposure and low temperature, impacting the DALYs of HHD less due to lower environmental exposure. Conversely, risk factors that account for higher proportions, like high BMI, high salt diet, and alcohol consumption, may be more strongly associated with an increase in poor lifestyle habits.

The frontier analysis indicated significant heterogeneity in effective difference across different SDI ranges. Countries with lower SDI, such as Niger (SDI = 0.162), have achieved excellent results despite limited resources, while the Cook Islands, as a higher SDI country (SDI = 0.764), had a higher age-standardised DALY rates and effective difference. This reflects the GBD theme 'development is not destiny' [31,32], suggesting that a country or region's status in development should not impede its ability to adjust policies and utilise existing resources to seize potential opportunities to reduce the burden of HHD. Given the limited health care resources in low and middle-income countries, we encourage governments and health care institutions in these nations to alleviate the burden of HHD through measures such as improving sanitary conditions, strengthening early screening, and promoting healthy lifestyles [19,33]. Overall, we recommend that areas at high risk for HHD adopt a population-wide health strategy that encourages screening and monitoring of blood pressure among community. In response to risk factors, there needs to be a focus on initiatives to control BMI and high sodium diets and reduce alcohol consumption [1,15,16,19,33].

This study has certain limitations. The definition of HHD in the GBD 2019 study is relatively narrow, lacking detailed information on the clinical and pathological characteristics of HHD, such as ventricular hypertrophy and its degree of severity [7,23]. The definition of ICD-coded HHD may not be applicable and generalised in different countries worldwide, leading to potential bias such as the effect of missing data in some contexts. Nevertheless, the GBD data set measured 95% UI to approximate the exact results as closely as possible. The occurrence of HHD increases the risk of stroke and coronary artery disease, independent of blood pressure, and contributes to an increased all-cause mortality [13,53]. Additionally, the number of deaths attributed to HHD is relatively low compared to its prevalence and DALYs, with global deaths totalling to 925 675 in 2017 and the age-standardised death rate in 2019 being 15.16 per 100 000 population [20,22]. Considering that HHD is not a primary cause of death and its mortality rate is low, we did not include mortality as a measure, instead focussing on prevalence and DALYs to explore the burden of HHD. Most of the risk factors for HHD in our study were derived from self-reporting, which may have some recall bias. However, the common risk factors for hypertension and HHD need to be emphasised and controlled for to reduce the burden of HHD. Simultaneously, strengthening hypertension screening in high-risk areas and in older populations, and increasing home-based self-monitoring of blood pressure and timely interventions are strategies that can help to improve the management of hypertension and HHD globally. Despite these limitations, our study provides a comprehensive review of the global, regional, and national burden and trends of HHD.

## CONCLUSIONS

We found that from 1990 to 2019, despite a decline in the age-standardised DALY rates for HHD, the prevalence has significantly increased globally. The affected population were mainly older adults aged over 70, with elderly women bearing a heavier burden. Attention must be given to the continuous upward trend in HHD prevalence in high-middle SDI quintile countries, although it should also be acknowledged that the middle SDI quintile countries bear a higher burden of HHD DALYs, with considerable opportunity to narrow the effective difference between observed and achievable age-standardised DALY rates. High BMI is a significant risk factor contributing to the burden of HHD. The formulation of more concrete and effective public health strategies for the early detection and management of HHD remains crucial for health care institutions to address the growing burden of HHD.

## Additional material


Online Supplementary Document
