# Supplementary material for: Global prevalence and disability-adjusted life years of hypertensive heart disease: A trend analysis from the Global Burden of Disease Study 2019
Source: J Glob Health. 2024 Aug 30;14:04172. doi: 10.7189/jogh.14.04172 (PMC11364089; doi:10.7189/jogh.14.04172)
Supplement: Online Supplementary Document [file jogh-14-04172-s001.pdf]

Supplementary Material Content

1. Supplementary Methods

1.1 Description of GBD regions and countries

1.2 Definitions of hypertensive heart disease

1.3 Decomposition analysis

1.4 Frontier analysis

2. **Supplementary Figures**

2.1 **Figure S1**. Global changes of age-standardized rates (ASR) of prevalence (A) and DALYs (B) of hypertensive heart disease from 1990 to 2019 by sex. Global changes of ASR of prevalence (C) and DALYs (D) of hypertensive heart disease from 1990 to 2019 by SDI quintiles. Global changes crude rate of prevalence (E) and DALYs (F) of hypertensive heart disease from 1990 to 2019 by age. ALYs, disability-adjusted life years.

2.2 **Figure S2**. All-age rates of prevalence (A) and DALYs (B) of hypertensive heart disease for both sexes in 204 countries and territories, 2019. The percentage change in age-standardized prevalence (C) and DALYs (D) rates of hypertensive heart disease for both sexes in 204 countries and territories from 1990 to 2019. DALYs, disability-adjusted life years.

2.3 **Figure S3**. Trend in age-standardized prevalence (A) and DALYs (B) rates of hypertensive heart disease globally and for 21 geographic regions by SDI, 1990–2019. Thirty points are plotted for each of the 21 geographic regions, with points from left to right depicting estimates for each year from 1990 to 2019. DALYs, disability-adjusted life years; SDI, socio-demographic index.

2.4 **Figure S4**. Hypertensive heart disease related burden in 204 countries and territories ranked by SDI in 1990 and 2019. Crude prevalence rate (A), age-standardized prevalence rate (B), Crude DALYs rate (C), and age-standardized DALYs rate (D) of hypertensive heart disease in 1990 and 2019 in 204 countries and territories, for both sexes. DALYs. disability-adjusted life years; SDI. socio-demographic index.

2.5 **Figure S5**. Percentage contributions of major risk factors to DALYs of hypertensive heart disease in 1990 (A) and 2019 (B). DALYs, disability-adjusted life years.

3. **Supplementary Tables**

3.1 **Table S1**. Numbers and age-standardized prevalence rates of hypertensive heart disease in 1990 and 2019, percentage change in ASR from 1990 to 2019, and their average annual percentage changes (AAPCs) from 1990 to 2019 at global, SDI groups, and geographic regions.

3.2 **Table S2**. Numbers and age-standardized DALYs rates of hypertensive heart disease in 1990 and 2019, percentage change in ASR from 1990 to 2019, and their average annual percentage changes (AAPCs) from 1990 to 2019 at global, SDI groups, and geographic regions.

3.3 **Table S3.** Prevalent cases of hypertensive heart disease in 1990 and 2019, percentage change in the age-standardized rates (ASRs) per 100,000, and their average annual percentage changes (AAPCs) from 1990 to 2019 by location.

3.4 **Table S4.** DALYs due to hypertensive heart disease in 1990 and 2019, percentage change in the age-standardized rates (ASRs) per 100,000, and their average annual percentage changes (AAPCs) from 1990 to 2019 by location.

3.5 **Table S5.** Changes in DALYs number according to population-level determinants from 1990 to 2019.

3.6 **Table S6.** Frontier DALYs, and effective difference by country or territory.

Supplementary Methods

Description of GBD regions and countries

GBD regions	Countries
High-income Asia Pacific	Brunei Darussalam, Japan, Republic of Korea, Singapore
High-income North America	Canada, United States of America
Western Europe	Andorra, Austria, Belgium, Cyprus, Denmark, Finland, France, Germany, Greece, Guernsey, Iceland, Ireland, Isle of Man, Israel, Italy, Jersey, Liechtenstein, Luxembourg, Malta, Monaco, Netherlands, Norway, Portugal, San Marino, Spain, Sweden, Switzerland, United Kingdom, Vatican City
Australasia	Australia, New Zealand
Andean Latin America	Bolivia, Ecuador, Peru
Tropical Latin America	Brazil, Paraguay
Central Latin America	Colombia, Costa Rica, El Salvador, Guatemala, Honduras, Mexico, Nicaragua, Panama, Venezuela (Bolivarian Republic of)
Southern Latin America	Argentina, Chile, Uruguay
Caribbean	Antigua and Barbuda, Bahamas, Barbados, Belize, Cuba, Dominica, Dominican Republic, Grenada, Guyana, Haiti, Jamaica, Saint, Kitts and Nevis, Saint Lucia, Saint Vincent and the Grenadines, Suriname, Trinidad and Tobago
Central Europe	Albania, Bosnia and Herzegovina, Bulgaria, Croatia, Czechia, Hungary, Kosovo, Montenegro, North Macedonia, Poland, Romania, Serbia, Slovakia, Slovenia
Eastern Europe	Belarus, Estonia, Latvia, Lithuania, Republic of Moldova, Russian Federation, Ukraine
Central Asia	Armenia, Azerbaijan, Georgia, Kazakhstan, Kyrgyzstan, Mongolia, Tajikistan, Turkmenistan, Uzbekistan
North Africa and Middle East	Afghanistan, Algeria, Bahrain, Egypt, Iran (Islamic Republic of), Iraq, Jordan, Kuwait, Lebanon, Libya, Morocco, Oman, Palestine, Qatar, Saudi Arabia, Syrian Arab Republic, Tunisia, Turkiye, United Arab Emirates, Western Sahara, Yemen
South Asia	Bangladesh, Bhutan, India, Nepal, Pakistan
Southeast Asia	Cambodia, Indonesia, Lao People's Democratic Republic, Malaysia, Maldives, Mauritius, Myanmar, Philippines, Seychelles, Sri Lanka, Thailand, Timor-Leste, Viet Nam
East Asia	China, Democratic People's Republic of Korea, Taiwan (Province of China)
Oceania	Fiji, Kiribati, Marshall Islands, Micronesia (Federated States of), Nauru, Niue, Palau, Papua New Guinea, Samoa, Solomon, Islands, Tonga, Tuvalu, Vanuatu
Western Sub-Saharan Africa	Benin, Burkina Faso, Cabo Verde, Cameroon, Chad, Cote d'Ivoire, Gambia, Ghana, Guinea, Guinea-Bissau, Liberia, Mali, Mauritania, Niger, Nigeria, Sao Tome and Principe, Senegal, Sierra Leone, Togo
Eastern Sub-Saharan Africa	Burundi, Comoros, Djibouti, Eritrea, Ethiopia, Kenya, Madagascar, Malawi, Mozambique, Rwanda, Somalia, South Sudan, Sudan, Uganda, United Republic of Tanzania, Zambia
Central Sub-Saharan Africa	Angola, Central African Republic, Congo, Democratic Republic of the Congo, Equatorial Guinea, Gabon
Southern Sub-Saharan Africa	Botswana, Eswatini, Lesotho, Namibia, South Africa, Zimbabwe

Definitions of hypertensive heart disease

ICD-9		ICD-10	
402.0	Malignant hypertensive heart disease	I11.0	Hypertensive heart disease with heart failure
402.00	Malignant hypertensive heart disease without heart failure	I11.9	Hypertensive heart disease without heart failure
402.01	Hypertensive heart disease with heart failure		
402.1	Benign hypertensive heart disease		
402.10	Benign hypertensive heart disease without heart failure		
402.11	Benign hypertensive heart disease with heart failure		
402.9	Unspecified hypertensive heart disease		
402.90	Unspecified hypertensive heart disease without heart failure		
402.91	Unspecified hypertensive heart disease with heart failure		

ICD, International Classification of Diseases.

Decomposition analysis

We used the decomposition methods developed by Das Gupta to decompose the disability-adjusted life-years (DALYs) of hypertensive heart disease by population aging, population growth and epidemiologic changes [1, 2]. DALY is calculated as:

$$DALY_{a,g,e,t} = \sum_{k=1}^{20} a_{k,t} * p_t * e_{k,t}$$

where $DALY_{a,g,e,t}$ represents DALYs number cumulated by population aging, population growth and epidemiologic changes in year t, $a_{k,t}$ is the proportion of population for the age group k at year t, p_t is the population size at year t, and $e_{k,t}$ is represented by DALYs rate for a specific age group k at year t.

We determined the effect of one factor on DALYs change on the basis that other factors remained unchanged. Therefore, the formula goes as follows if we calculated the contribution of population aging to DALYs change**:**

$$Effect_{a_{2019}} = \left[\frac{DALY_{a_{2019},p_{2019},e_{2019}} + DALY_{a_{2019},p_{1990},e_{1990}}}{3} + \frac{DALY_{a_{2019},p_{1990},e_{2019}} + DALY_{a_{2019},p_{2019},e_{1990}}}{6}\right] - \left[\frac{DALY_{a_{1990},p_{2019},e_{2019}} + DALY_{a_{1990},p_{1990},e_{1990}}}{3} + \frac{DALY_{a_{1990},p_{1990},e_{2019}} + DALY_{a_{1990},p_{2019},e_{1990}}}{6}\right]$$

Frontier analysis

We carried out an improved data envelopment analysis (DEA) in frontier analysis [1-3]. DEA is a non-parametric method, which is used to estimate the production boundary under the convexity assumption. “Free disposal hull” (fdh) option was selected to model the ideal achievable age-standardized DALYs on the basis of sociodemographic index. In order to minimize the modelling error as it as possible, 100 bootstrapped samples of the data were put into model with incorporation of year, location and other covariates. Mean age-standardized DALYs for the given sociodemographic index were calculated from 100 bootstrapped sampling. A smooth frontier (span=0.3, degree=1) was then fitted though Loess regression. In the process of boundary generation, outliers were eliminated by excluding super-efficient elements. The

distance between the observed age-standardized DALYs and the frontier values, named "effective difference", was to determine the gap of age-standardized DALYs that could be attained at a given level of development.

For example, when the observed rate of a country was far below the frontier value given its sociodemographic index, it implied there might be a great deal of unrecognized opportunities for the country for improvement in terms of age standardized DALYs. Besides, the change of effective difference for a country over time provided a pattern of the changing burden with sociodemographic index.

Supplementary References:

1. Li H, Lu W, Wang A, Jiang H, Lyu J: Changing epidemiology of chronic kidney disease as a result of type 2 diabetes mellitus from 1990 to 2017: Estimates from Global Burden of Disease 2017. Journal of diabetes investigation, 12: 346-356, 2021. DOI: 10.1111/jdi.13355.
2. Xie Y, Bowe B, Mokdad AH, Xian H, Yan Y, Li T, Maddukuri G, Tsai CY, Floyd T, Al-Aly Z: Analysis of the Global Burden of Disease study highlights the global, regional, and national trends of chronic kidney disease epidemiology from 1990 to 2016. Kidney international, 94: 567-581, 2018. DOI: 10.1016/j.kint.2018.04.011.
3. Access GBDH, Quality Collaborators. Electronic address cue, Access GBDH, Quality C: Healthcare Access and Quality Index based on mortality from causes amenable to personal health care in 195 countries and territories, 1990-2015: a novel analysis from the Global Burden of Disease Study 2015. Lancet, 390: 231-266, 2017. DOI: 10.1016/S0140-6736(17)30818-8.

Supplementary Figures

Figure S1. Global changes of age-standardized rates (ASR) of prevalence (A) and DALYs (B) of hypertensive heart disease from 1990 to 2019 by sex. Global changes of ASR of prevalence (C) and DALYs (D) of hypertensive heart disease from 1990 to 2019 by SDI quintiles. Global changes crude rate of prevalence (E) and DALYs (F) of hypertensive heart disease from 1990 to 2019 by age. ALYs, disability-adjusted life years.

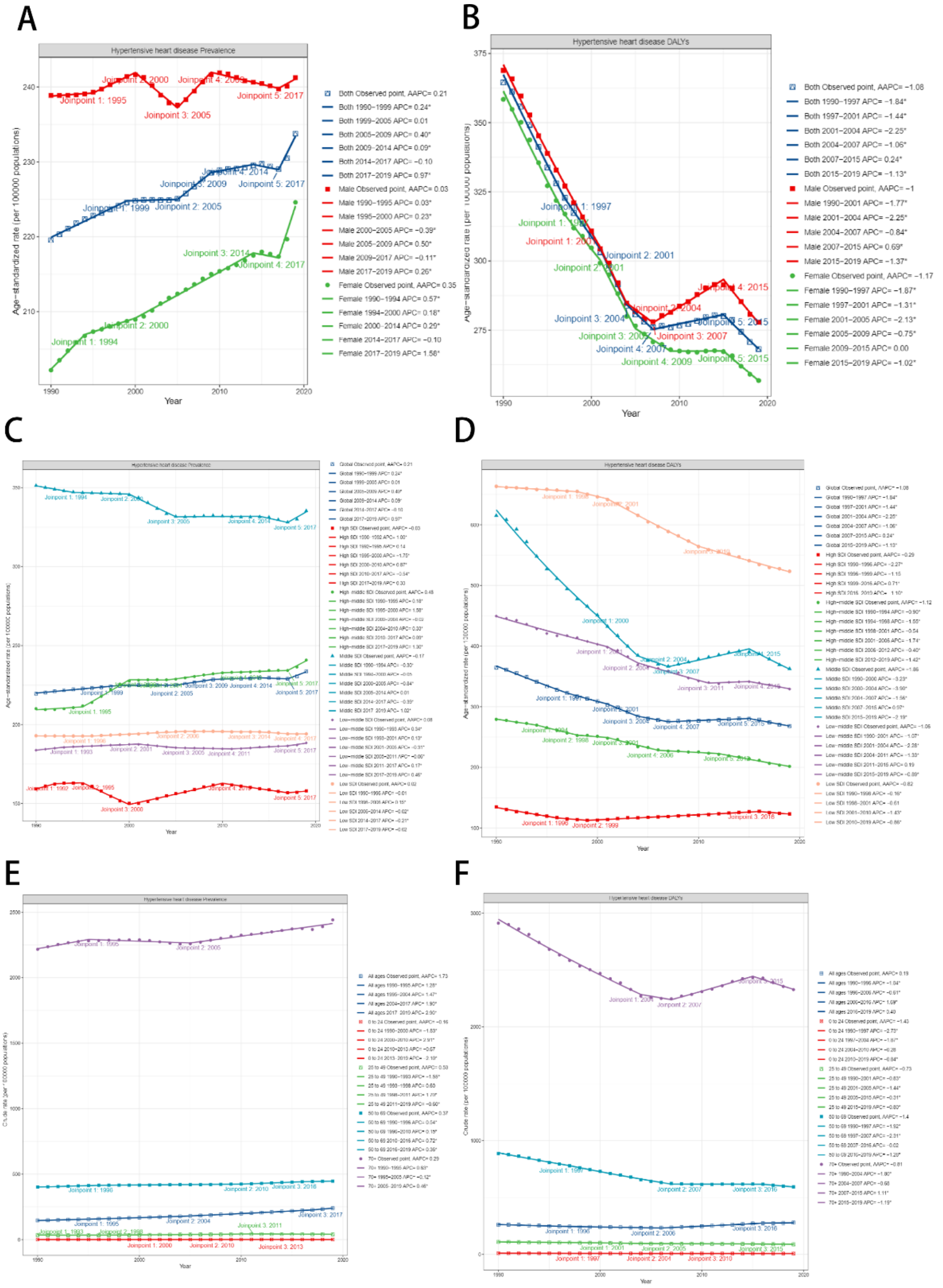

Figure S2. All-age rates of prevalence (A) and DALYs (B) of hypertensive heart disease for both sexes in 204 countries and territories, 2019. The percentage change in age-standardized prevalence (C) and DALYs (D) rates of hypertensive heart disease for both sexes in 204 countries and territories from 1990 to 2019. DALYs, disability-adjusted life years.

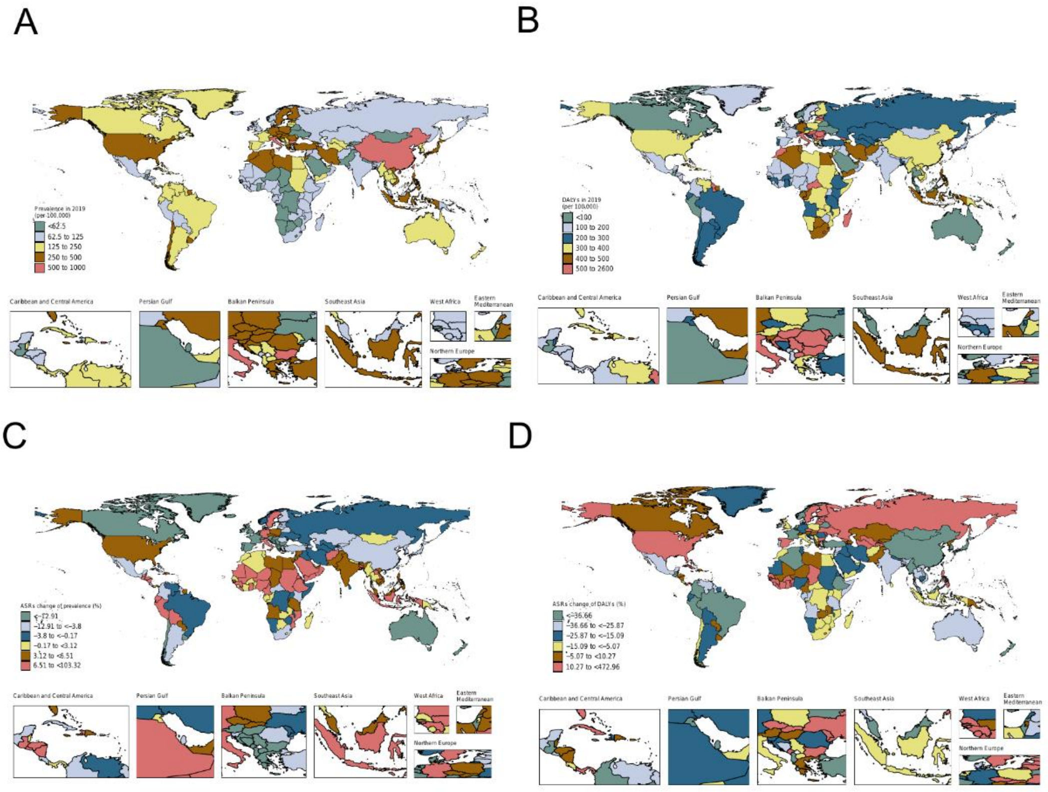

Figure S3. Trend in age-standardized prevalence (A) and DALYs (B) rates of hypertensive heart disease globally and for 21 geographic regions by SDI, 1990–2019. Thirty points are plotted for each of the 21 geographic regions, with points from left to right depicting estimates for each year from 1990 to 2019. DALYs, disability-adjusted life years; SDI, socio-demographic index.

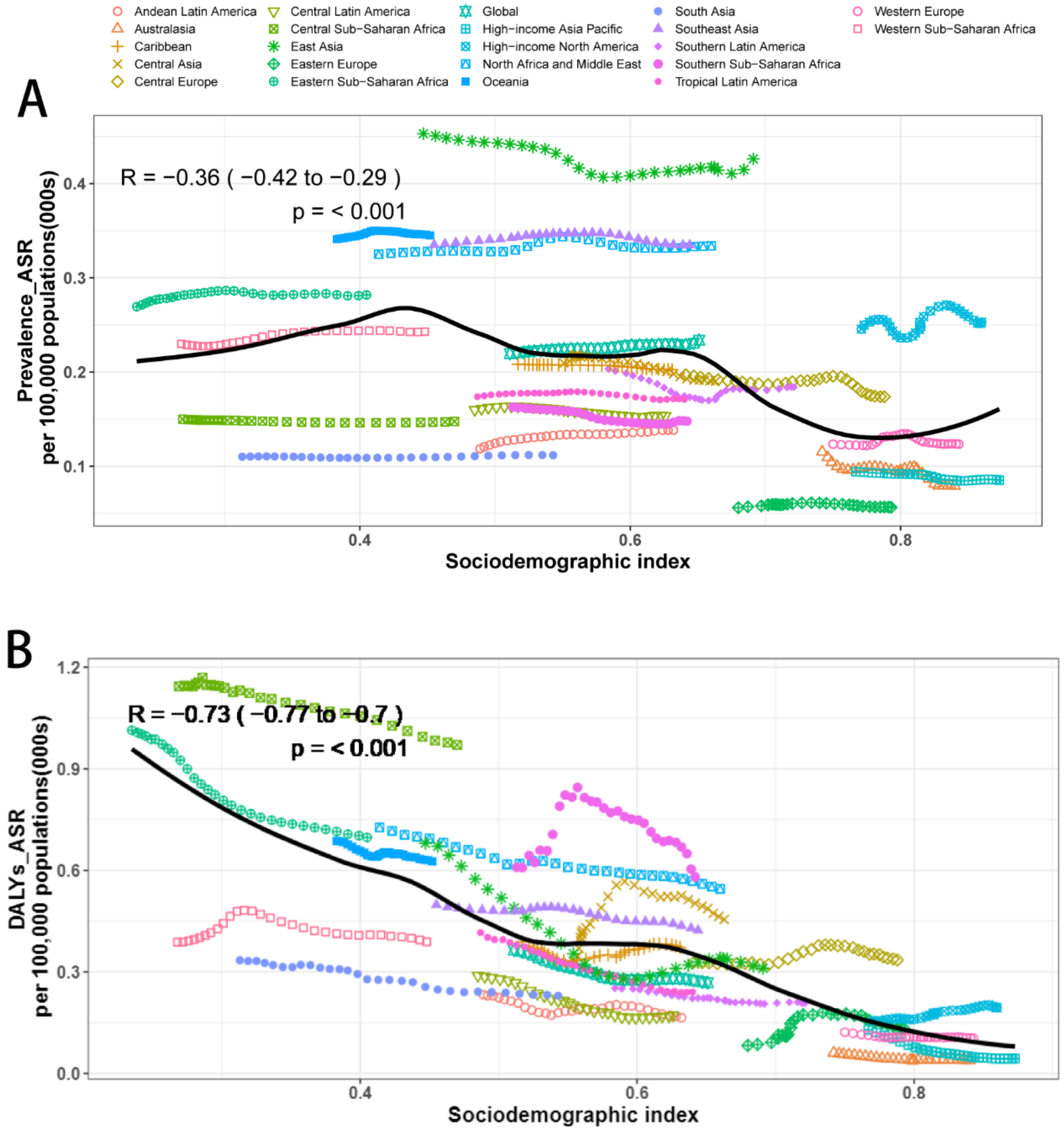

Figure S4. Hypertensive heart disease related burden in 204 countries and territories ranked by SDI in 1990 and 2019. Crude prevalence rate (A), age-standardized prevalence rate (B), Crude DALYs rate (C), and age-standardized DALYs rate (D) of hypertensive heart disease in 1990 and 2019 in 204 countries and territories, for both sexes. DALYs. disability-adjusted life years; SDI. socio-demographic index.

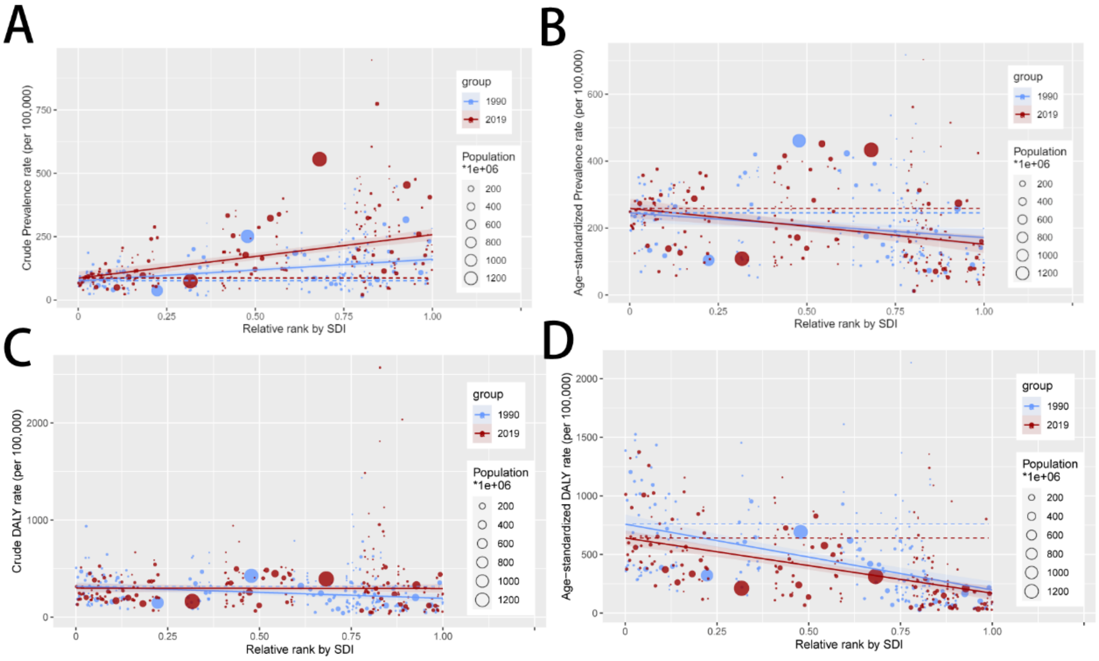

Figure S5. Percentage contributions of major risk factors to DALYs of hypertensive heart disease in 1990 (A) and 2019 (B). DALYs, disability-adjusted life years.

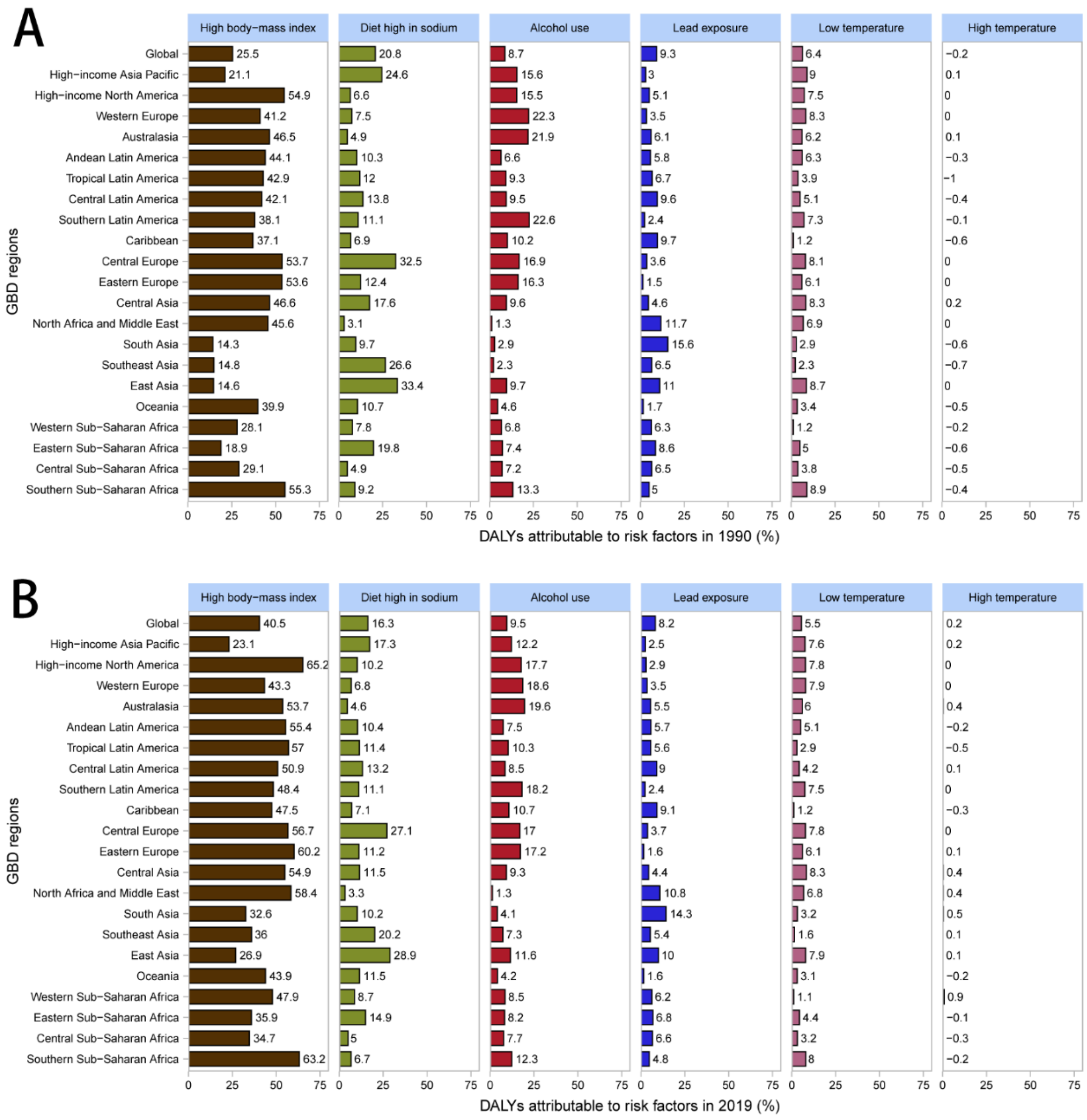

Supplementary Tables

Table S1. Numbers and age-standardized prevalence rates of hypertensive heart disease in 1990 and 2019, percentage change in ASR from 1990 to 2019, and their average annual percentage changes (AAPCs) from 1990 to 2019 at global, SDI groups, and geographic regions.

	1990-Prevalence		2019-Prevalence		Percentage change in ASRs from 1990 to 2019 (%)	AAPC (95% CI)
	No. in thousands (95% UI)	ASRs per 100000 (95% UI)	No. in thousands (95% UI)	ASRs per 100000 (95% UI)		
Global	7817.3 (5616.2,10643)	219.5 (158.8,299.4)	18598 (13544.4,24898.4)	233.8 (170.5,312.9)	6.5 (3.6,9.8)	0.21 (0.17, 0.24)
**SDI groups**						
High SDI	1634.7 (1192,2187.4)	159.2 (117.5,211.2)	3062.7 (2270.6,4089)	158 (118.6,206.9)	-0.8 (-8.6,7.9)	-0.03 (-0.09, 0.03)
High-middle SDI	2005.6 (1405.6,2840.4)	210.1 (149.9,295.7)	4848.2 (3464.8,6677.5)	240.9 (172.6,328.7)	14.6 (9.8,20.1)	0.48 (0.43, 0.52)
Middle SDI	2956 (2110.3,4004)	351.4 (253,473.8)	7578.9 (5501.5,10180.2)	335.4 (243.8,449.2)	-4.5 (-6.7, -2.2)	-0.17 (-0.2, -0.13)
Low-middle SDI	849.5 (598.3,1181.9)	183.8 (131.7,256.3)	2248.3 (1607.9,3122.6)	188.4 (135.2,261.5)	2.5 (0.3,4.8)	0.08 (0.07, 0.1)
Low SDI	367.3 (256.1,514.5)	192.9 (135.5,268)	850.3 (595.1,1169.1)	194.1 (136.7,268.9)	0.6 (-1.4,2.9)	0.02 (0, 0.04)
**Geographic regions**						
High-income Asia Pacific	166.8 (114.3,231.9)	94.2 (65.1,131.6)	434.6 (304.5,608.3)	85.1 (61.5,114.6)	-9.7 (-21.8,5)	-0.34 (-0.37, -0.32)
High-income North America	843.3 (603,1130.9)	203.4 (136.8,295.5)	1540.5 (1125.4,2037.7)	253.1 (188.8,330.6)	3.1 (-9.7,18)	0.09 (-0.02, 0.2)
Western Europe	738.8 (523.9,1040.8)	452.9 (326.2,616.5)	1298.3 (923.6,1798.4)	123.5 (89.2,169.2)	0.1 (-6.4,7.6)	0.02 (-0.03, 0.07)
Australasia	26.1 (18.8,35.7)	115.3 (83.8,156.4)	42.6 (30.5,57.3)	147.8 (95.8,216)	-31.4 (-37, -25.7)	-1.27 (-1.36, -1.18)
Andean Latin America	21 (14.1,30.9)	118.6 (79.4,174.2)	74.4 (51.5,105.6)	138.4 (95.1,196.9)	16.7 (7.6,28.9)	0.53 (0.5, 0.56)
Tropical Latin America	128.4 (87.3,183.8)	173.8 (120.6,246.8)	392.8 (272.3,557.7)	171 (118.4,242.8)	-1.6 (-5,2.4)	-0.05 (-0.1, 0)
Central Latin America	113.2 (79.2,160.5)	269.4 (184.8,372.8)	343.6 (244.6,482.5)	153.5 (109,216.1)	-4 (-7.2, -0.5)	-0.14 (-0.17, -0.11)
Southern Latin America	86.5 (58,127.9)	159.9 (112.3,227.4)	158.6 (108.7,229.2)	184 (127,265.4)	-9.5 (-17.2, -1.1)	-0.34 (-0.41, -0.26)
Caribbean	51.5 (37.4,70.9)	208.4 (152,285.1)	104.8 (76.6,144.5)	202.5 (147.9,279.7)	-2.8 (-6.5,1.5)	-0.09 (-0.1, -0.08)

Central Europe	272.4 (187.1,391.8)	197.3 (138.5,280.2)	392.4 (275.3,556)	173.8 (123.5,242)	-11.9 (-19.6, -2.9)	-0.43 (-0.48, -0.38)
Eastern Europe	146.9 (97.9,218.3)	56.1 (38.3,81.9)	198.9 (133.6,295.1)	56.5 (38.2,83)	0.7 (-2.5,3.4)	0.03 (-0.02, 0.08)
Central Asia	86.6 (62,117)	123.5 (89.5,169.7)	116.9 (83.4,159.1)	190.2 (133.2,262.4)	-9.2 (-14.9, -4.3)	-0.33 (-0.38, -0.28)
North Africa and Middle East	505 (359.7,689.9)	150 (97.3,224.1)	1331.8 (975.3,1764.5)	333.9 (249.1,446.5)	2.7 (-2.2,8.3)	0.09 (0.06, 0.11)
South Asia	432.6 (302.8,619.9)	110.2 (77.4,156.8)	1305.1 (917.1,1870.8)	79.1 (57.2,106.2)	1.5 (-0.6,3.5)	0.05 (0.04, 0.06)
Southeast Asia	737.4 (527.5,986.8)	335 (244.4,456.5)	1847.7 (1330.7,2473)	334.8 (244.8,451.6)	-0.1 (-3.3,3.4)	0 (-0.02, 0.02)
East Asia	3042.4 (2152.4,4183.5)	209.4 (150.7,285.6)	8080.4 (5821.1,11009.5)	426.1 (306.6,574.8)	-5.9 (-8.6, -2.8)	-0.21 (-0.26, -0.16)
Oceania	7.8 (5.5,10.5)	341 (241.6,471.3)	18.8 (13.4,25.7)	344.9 (248.5,477.9)	1.1 (-4.8,7.8)	0.04 (0.03, 0.05)
Western Sub-Saharan Africa	171.4 (121.2,233.7)	229.9 (161.2,310)	381.3 (267.1,513.6)	242.9 (168.7,330.3)	5.6 (2.2,8.9)	0.19 (0.15, 0.23)
Eastern Sub-Saharan Africa	171 (117.5,234.5)	245.5 (178.2,325.2)	394.2 (272.5,537.5)	281.8 (193.7,387.8)	4.6 (1.6,7.6)	0.15 (0.13, 0.18)
Central Sub-Saharan Africa	28.2 (18.2,43.4)	325 (237.4,443.5)	66 (43.8,99)	147.6 (95.6,222.5)	-1.6 (-8.6,5.5)	-0.06 (-0.06, -0.05)
Southern Sub-Saharan Africa	40 (26.2,58.7)	162.8 (105.9,237.5)	74.2 (48.4,110.5)	111.8 (78.3,159)	-9.2 (-13.1, -5.4)	-0.33 (-0.35, -0.31)

SDI: socio-demographic index; ASR: age-standardized rate; AAPC: average annual percent change; UI: uncertainty interval.

Table S2. Numbers and age-standardized DALYs rates of hypertensive heart disease in 1990 and 2019, percentage change in ASR from 1990 to 2019, and their average annual percentage changes (AAPCs) from 1990 to 2019 at global, SDI groups, and geographic regions.

	1990-DALYs		2019-DALYs		Percentage change in ASRs from 1990 to 2019 (%)	AAPC (95% CI)
	No. in thousands (95% UI)	ASRs per 100000 (95% UI)	No. in thousands (95% UI)	ASRs per 100000 (95% UI)		
Global	13943.6 (11312.1,15650.1)	364.6 (297.7,406.7)	21508 (16400.1,23899.9)	268.2 (204.6,298.1)	-26.4 (-35.5, -15.7)	-1.08 (-1.23, -0.93)
**SDI groups**						
High SDI	1390.2 (1182.9,1522.4)	135 (115.6,147.7)	2290.9 (1802.1,2506)	123.3 (95.1,134.6)	-8.6 (-27, -3.1)	-0.29 (-0.45, -0.13)
High-middle SDI	2799.9 (2519.9,3101.6)	279.3 (252.9,311.7)	4011.3 (3322.8,4444.4)	201.7 (166,223)	-27.8 (-43, -18.4)	-1.12 (-1.28, -0.96)
Middle SDI	5715.8 (3995.5,6479.1)	615.2 (439.1,691.6)	8320.7 (5669.7,9286.8)	362.6 (246.5,406)	-41.1 (-49.2, -30.9)	-1.86 (-2.04, -1.69)
Low-middle SDI	2496.9 (1737.4,3028.7)	449.5 (321.8,543.4)	4239.7 (3498.2,4991.1)	329.1 (272.6,388.2)	-26.8 (-37.6, -9.7)	-1.06 (-1.25, -0.87)
Low SDI	1532.7 (996.8,2036)	663.4 (448.7,880.7)	2629.3 (1800.1,3422.9)	523.3 (358.8,683.3)	-21.1 (-33, -5.7)	-0.82 (-0.89, -0.75)
**Geographic regions**						
High-income Asia Pacific	234.5 (153.1,252.8)	129.2 (81.3,139.9)	231.3 (187.4,320.8)	44 (36.5,64.6)	-65.9 (-71, -28.6)	-3.65 (-3.8, -3.5)
High-income North America	526 (451.7,581.9)	155.3 (133.2,171.8)	1102 (735.5,1201.1)	193.7 (126.5,210.2)	24.7 (-14.5,32.4)	0.76 (0.61, 0.91)
Western Europe	721.6 (623.7,807.7)	121.9 (105.6,136.7)	1123 (847.3,1253.2)	103.5 (80.7,115.7)	-15.1 (-34, -4.2)	-0.56 (-0.68, -0.44)
Australasia	13.7 (10.8,15.3)	60.5 (47.9,67.4)	20.4 (16.9,24.2)	38.9 (32.9,46.6)	-35.6 (-41, -9.9)	-1.51 (-1.65, -1.37)
Andean Latin America	47.2 (40.5,53.9)	232.3 (199.5,264.9)	90.9 (74.6,108.3)	164.6 (134.9,195.7)	-29.1 (-43.2, -13.3)	-1.23 (-1.47, -0.99)
Tropical Latin America	370.6 (285.8,391.5)	415.4 (325.7,440.7)	572.4 (515.6,781.2)	240.4 (215.7,327.9)	-42.1 (-47.6, -8.8)	-1.85 (-2.03, -1.67)
Central Latin America	224.8 (161.1,238.5)	288.8 (208.2,307.2)	392.5 (329.2,503.1)	171.5 (144.2,219.6)	-40.6 (-50.1, -6.3)	-1.81 (-2.06, -1.56)
Southern Latin America	112.5 (102.4,127.7)	251.7 (229.9,292.9)	175.5 (153.8,209.6)	207 (182.3,247.8)	-17.8 (-26.8, -7.8)	-0.65 (-0.93, -0.37)
Caribbean	100.7 (86.7,120.6)	388.8 (336.2,467.5)	192.4 (158.2,229.2)	372.3 (306.2,444.1)	-4.2 (-20.1,12.6)	-0.1 (-0.22, 0.01)
Central Europe	466.5 (437.9,562.1)	328.3 (306.6,398.8)	730.9 (546.3,846.7)	334.8 (249.4,387.4)	2 (-28.4,17.6)	0.06 (-0.02, 0.14)
Eastern Europe	225.5 (202.5,326.7)	82.7 (74,120)	447.2 (297.6,517.3)	131.7 (88.5,152.6)	59.3 (-6.5,85.1)	1.65 (0.9, 2.41)
Central Asia	147.4 (131.1,191.1)	325.3 (288.5,423.3)	292.5 (252.6,335)	454.6 (390,515.3)	39.7 (8.2,68.9)	1.22 (0.95, 1.48)

North Africa and Middle East	1153.3 (702.9,1469.1)	726.3 (448.4,913.1)	2180.4 (1285.2,2768.7)	545 (315.8,682.4)	-25 (-42, -7.4)	-0.99 (-1.13, -0.84)
South Asia	1684.2 (998.2,2289.4)	334.1 (206.3,452.4)	3002.4 (2193.4,3898.3)	229.2 (166.1,296.9)	-31.4 (-44.7, -5)	-1.27 (-1.72, -0.81)
Southeast Asia	1233 (859.6,1457.9)	497.9 (358.1,584.1)	2498.9 (1585.1,2834.2)	422.9 (274.2,476.8)	-15.1 (-29.6,4.1)	-0.54 (-0.64, -0.43)
East Asia	5144.4 (3759.9,5899.1)	680 (516,768.8)	5780.8 (4053.6,6713.5)	310.4 (217.5,359.4)	-54.4 (-63.6, -44.7)	-2.68 (-2.97, -2.38)
Oceania	21 (14.5,28.6)	686.4 (481.2,916.5)	46.4 (29.9,63.5)	626.3 (419.1,841.6)	-8.8 (-25.9,13.6)	-0.32 (-0.4, -0.24)
Western Sub-Saharan Africa	345.3 (250.3,439.7)	387.9 (287,490.3)	761.6 (475.9,972.6)	388.9 (247.3,487.7)	0.2 (-36.7,26.8)	0.03 (-0.07, 0.13)
Eastern Sub-Saharan Africa	752 (457.8,1090.5)	1013.4 (625.8,1450.8)	1077.4 (658.7,1587.1)	696.9 (418.4,1038.7)	-31.2 (-43.4, -9.5)	-1.28 (-1.35, -1.22)
Central Sub-Saharan Africa	250 (156.3,326)	1143.1 (733.8,1487.5)	479.6 (307.6,648.1)	970.3 (625.8,1310.4)	-15.1 (-33.3,7.3)	-0.56 (-0.65, -0.46)
Southern Sub-Saharan Africa	169.6 (150.4,196.2)	608.8 (535.7,712)	309.5 (277.9,349.1)	580.3 (519.5,648.9)	-4.7 (-17.5,5.9)	-0.16 (-0.73, 0.4)

DALYs: disability-adjusted life years; SDI: socio-demographic index; ASR: age-standardized rate; AAPC: average annual percent change; UI: uncertainty interval.

Table S3. Prevalent numbers of hypertensive heart disease in 1990 and 2019, percentage change in the age-standardized rates (ASRs) per 100,000, and their average annual percentage changes (AAPCs) from 1990 to 2019 by location.

	1990		2019		Percentage change in ASRs from 1990 to 2019	AAPC (95% CI)
	No. in thousands (95% UI)	ASRs per 100000 (95% UI)	No. in thousands (95% UI)	ASRs per 100000 (95% UI)		
Global	7817.3 (5616.2,10643)	219.5 (158.8,299.4)	18598 (13544.4,24898.4)	233.8 (170.5,312.9)	6.5 (3.6,9.8)	0.21 (0.17, 0.24)
Andean Latin America	21 (14.1,30.9)	118.6 (79.4,174.2)	74.4 (51.5,105.6)	138.4 (95.1,196.9)	16.7 (7.6,28.9)	0.53 (0.5, 0.56)
Bolivia (Plurinational State of)	1.2 (0.4,2.4)	52.5 (21,102)	7.8 (4.5,12.4)	106.8 (63.9,163.8)	103.3 (47,250.2)	2.51 (2.3, 2.73)
Ecuador	9.3 (6.3,13.6)	201.1 (136.8,288.6)	31.8 (21.9,44.9)	224 (154.7,315.6)	11.4 (0.2,27.7)	0.37 (0.36, 0.38)
Peru	10.6 (7.3,15.3)	100.2 (67.6,145.1)	34.8 (24.5,48.6)	109.7 (76.7,154.3)	9.5 (-1.4,23)	0.31 (0.3, 0.32)
Australasia	26.1 (18.8,35.7)	115.3 (83.8,156.4)	42.6 (30.5,57.3)	79.1 (57.2,106.2)	-31.4 (-37, -25.7)	-1.27 (-1.36, -1.18)
Australia	22.4 (16.1,30.7)	119.3 (86.6,161)	36.3 (25.7,49.4)	80.1 (57.4,108.1)	-32.8 (-38.9, -26.3)	-1.34 (-1.45, -1.22)
New Zealand	3.7 (2.5,5.4)	96.2 (65.9,140.3)	6.2 (4.3,8.6)	73.8 (51.6,100.8)	-23.3 (-34.2, -9.2)	-0.9 (-1, -0.8)
Caribbean	51.5 (37.4,70.9)	208.4 (152,285.1)	104.8 (76.6,144.5)	202.5 (147.9,279.7)	-2.8 (-6.5,1.5)	-0.09 (-0.1, -0.08)
Antigua and Barbuda	0.2 (0.1,0.2)	282.3 (215.8,356.3)	0.3 (0.2,0.4)	283 (208.1,385.4)	0.2 (-16.7,18.9)	0.01 (-0.02, 0.03)
Bahamas	0.5 (0.3,0.6)	336.7 (248.4,447.8)	1.1 (0.8,1.5)	332 (245.9,444.5)	-1.4 (-10.4,7.4)	-0.04 (-0.06, -0.03)
Barbados	0.7 (0.5,1)	225.3 (163.3,316.9)	1.1 (0.8,1.6)	224.2 (162.1,310.3)	-0.5 (-9.1,8.7)	-0.01 (-0.04, 0.01)
Belize	0.2 (0.2,0.3)	259.6 (187.9,363.2)	0.6 (0.5,0.9)	263.7 (187.4,369.6)	1.6 (-8.1,11.5)	0.05 (0.02, 0.09)
Bermuda	0.1 (0.1,0.1)	139.4 (99.5,192.7)	0.2 (0.1,0.3)	128.4 (93.4,183.8)	-7.9 (-16,1.9)	-0.28 (-0.3, -0.26)
Cuba	16.2 (11.7,23.1)	159.5 (116.7,222.6)	28.3 (20.6,39.6)	144.2 (105.1,200.8)	-9.6 (-18.1, -1.4)	-0.35 (-0.38, -0.31)
Dominica	0.2 (0.1,0.3)	271 (198.1,370.7)	0.2 (0.2,0.3)	263.5 (190.5,356.5)	-2.8 (-12,6.2)	-0.09 (-0.12, -0.07)
Dominican Republic	7.4 (5.3,10.3)	227.1 (164.2,314.6)	20.7 (14.9,29.2)	236.9 (168.8,336.5)	4.4 (-4.9,15)	0.14 (0.12, 0.16)
Grenada	0.2 (0.1,0.2)	220.6 (157.8,305.1)	0.2 (0.2,0.3)	221.3 (159.1,306)	0.3 (-9.1,10.1)	0.01 (-0.01, 0.03)
Guyana	0.9 (0.7,1.3)	288.5 (209.6,394.9)	1.4 (1,2)	277.1 (198.2,377.2)	-3.9 (-12.8,5.4)	-0.14 (-0.15, -0.13)

Haiti	4.8 (3.3,6.7)	186.3 (133.6,256.5)	11.2 (7.9,15.5)	201.3 (142.5,281.7)	8.1 (-2.8,19.7)	0.27 (0.25, 0.28)
Jamaica	5.6 (4.1,7.4)	304.6 (222.3,398.4)	9.8 (7.3,12.7)	317.8 (234.4,416.8)	4.3 (-4.4,13)	0.15 (0.12, 0.17)
Puerto Rico	9.4 (6.6,13.3)	258.5 (187.3,360)	18.6 (13.1,26.7)	239.4 (169.9,335.1)	-7.4 (-15.8,2.3)	-0.26 (-0.27, -0.25)
Saint Kitts and Nevis	0.1 (0.1,0.1)	219.4 (157,307.5)	0.1 (0.1,0.2)	216 (153,305.2)	-1.5 (-10.8,8.9)	-0.05 (-0.07, -0.03)
Saint Lucia	0.2 (0.2,0.3)	258.5 (188.7,354.5)	0.5 (0.4,0.7)	256.2 (184.7,355.5)	-0.9 (-9.2,9.2)	-0.03 (-0.04, -0.02)
Saint Vincent and the Grenadines	0.2 (0.1,0.3)	291.3 (212.2,396)	0.4 (0.3,0.5)	290.5 (209.4,395.6)	-0.3 (-9.2,10.5)	-0.01 (-0.02, 0)
Suriname	0.6 (0.4,0.8)	256.3 (184.2,353.7)	1.5 (1.1,2)	265.3 (194.2,365.6)	3.5 (-6.5,14)	0.12 (0.09, 0.14)
Trinidad and Tobago	2.1 (1.5,2.9)	263 (188,366.3)	4.5 (3.2,6.4)	252.8 (182,354)	-3.9 (-12.6,5.3)	-0.14 (-0.15, -0.12)
United States Virgin Islands	0.2 (0.1,0.2)	214.2 (153.8,295.8)	0.4 (0.3,0.5)	197.7 (139.9,278.4)	-7.7 (-16.2,0.8)	-0.27 (-0.31, -0.24)
Central Asia	86.6 (62,117)	209.4 (150.7,285.6)	116.9 (83.4,159.1)	190.2 (133.2,262.4)	-9.2 (-14.9, -4.3)	-0.33 (-0.38, -0.28)
Armenia	2.9 (2,4.1)	129.6 (90.5,185.5)	4.9 (3.3,6.9)	121.3 (82,172.4)	-6.4 (-15.3,4.5)	-0.23 (-0.26, -0.19)
Azerbaijan	7.7 (5.4,11)	180.4 (124.1,261.3)	12 (8.1,17.3)	165.7 (113,242.6)	-8.2 (-17.5,2.3)	-0.29 (-0.34, -0.25)
Georgia	14.3 (9.8,20.7)	258.6 (178.6,366.2)	14.1 (9.3,20.5)	220.6 (145.1,323.7)	-14.7 (-27, -4.1)	-0.54 (-0.57, -0.52)
Kazakhstan	14.4 (9.8,20.6)	128.6 (88.1,183.9)	17.4 (11.7,25.5)	114.5 (77,165.5)	-11 (-21, -0.5)	-0.39 (-0.47, -0.32)
Kyrgyzstan	2.9 (2,4.2)	103.2 (71.5,148.6)	3.7 (2.6,5.3)	95.9 (65.9,137.7)	-7.1 (-15.2,2.4)	-0.26 (-0.29, -0.22)
Mongolia	1 (0.7,1.4)	103.4 (72,149.2)	1.9 (1.3,2.7)	103.3 (71,150.7)	-0.2 (-10.8,11.4)	0 (-0.05, 0.05)
Tajikistan	6.2 (4.4,8.3)	235.1 (166.1,324.1)	8.4 (5.9,11.6)	225.6 (159.4,316.1)	-4 (-13.7,6.9)	-0.14 (-0.17, -0.11)
Turkmenistan	3.8 (2.5,5.2)	223.3 (151.1,315.7)	7.4 (5.1,10.3)	220.6 (150.3,315.8)	-1.2 (-11.6,9.8)	-0.05 (-0.08, -0.02)
Uzbekistan	33.5 (24.9,45)	329.3 (241,444)	47.3 (33.3,63.9)	320.6 (230.2,430)	-2.6 (-12.8,7.6)	-0.09 (-0.12, -0.07)
Central Europe	272.4 (187.1,391.8)	197.3 (138.5,280.2)	392.4 (275.3,556)	173.8 (123.5,242)	-11.9 (-19.6, -2.9)	-0.43 (-0.48, -0.38)
Albania	2 (1.4,2.9)	112.7 (76.4,160.7)	4 (2.7,5.9)	92.5 (62,133.5)	-17.9 (-26.3, -8.8)	-0.68 (-0.71, -0.64)
Bosnia and Herzegovina	4.8 (3.3,6.8)	143 (98.6,203.2)	6.5 (4.3,9.6)	110.4 (74.3,158.4)	-22.8 (-32.1, -13.2)	-0.88 (-0.93, -0.84)
Bulgaria	38 (25.2,54.9)	333.1 (231,466.8)	41.9 (27.9,60.5)	270.1 (185.4,379)	-18.9 (-28.4, -9.2)	-0.72 (-0.75, -0.68)
Croatia	10.8 (7.1,15.9)	181.6 (121.3,262.7)	10.8 (7.4,15.5)	112 (77.6,158.7)	-38.3 (-52.1, -20.5)	-1.66 (-1.71, -1.61)
Czechia	15.6 (10.9,21.9)	117 (82.9,161.2)	26.8 (18.2,37.6)	120.9 (84.1,167.1)	3.4 (-14.3,26.6)	0.11 (-0.02, 0.23)
Hungary	36.2 (24.1,53.4)	251.2 (169.9,363.7)	34.9 (21.6,54)	168.2 (106.6,254.3)	-33.1 (-43.7, -24.2)	-1.37 (-1.41, -1.33)

Montenegro	0.4 (0.3,0.6)	75.1 (51.8,109.3)	0.6 (0.4,0.8)	57.5 (39.2,82.9)	-23.5 (-32.2, -14)	-0.92 (-0.94, -0.9)
North Macedonia	3.6 (2.4,5.1)	226.5 (155.3,324.9)	5.3 (3.5,8.1)	181.4 (123.4,269.1)	-19.9 (-29, -10.7)	-0.77 (-0.78, -0.75)
Poland	77.5 (52.9,113.5)	188.5 (130.8,272.4)	143.5 (100,203.6)	196.8 (137.5,278.7)	4.4 (-7.7,18.6)	0.16 (0.11, 0.2)
Romania	56.8 (37.1,83.5)	222.3 (148.6,317.2)	80.8 (52.1,121.2)	200.3 (129.5,297.5)	-9.9 (-25.6,7.1)	-0.38 (-0.45, -0.32)
Serbia	12.4 (8.2,17.9)	125.4 (82.5,182.1)	12.4 (7.5,18.9)	74.8 (46.3,111.5)	-40.3 (-55.9, -23.8)	-1.78 (-1.85, -1.7)
Slovakia	9.4 (6.4,13.6)	160.4 (112.1,227.4)	15.6 (11.3,21.2)	167.6 (122.2,225.1)	4.5 (-15.4,32.6)	0.14 (0.06, 0.23)
Slovenia	4.9 (3.3,7.3)	206.1 (142.2,299.9)	9.2 (6.7,12.5)	189.2 (138.1,254.1)	-8.2 (-24.9,16.1)	-0.26 (-0.35, -0.17)
Central Latin America	113.2 (79.2,160.5)	159.9 (112.3,227.4)	343.6 (244.6,482.5)	153.5 (109,216.1)	-4 (-7.2, -0.5)	-0.14 (-0.17, -0.11)
Colombia	27.8 (19.5,39.4)	187.4 (131.9,265.6)	93.7 (67.1,129.8)	175.1 (124.7,245.8)	-6.5 (-14.8,3.3)	-0.24 (-0.29, -0.18)
Costa Rica	2.8 (2,4)	175.1 (123.1,245.9)	8.3 (5.9,11.5)	164.7 (116.5,228.9)	-5.9 (-15.4,3.9)	-0.2 (-0.23, -0.18)
El Salvador	2.9 (1.9,4.1)	104.3 (71.1,151.2)	7.1 (5,9.9)	114.6 (80,161.4)	9.9 (-1.8,22.6)	0.33 (0.3, 0.35)
Guatemala	2.1 (1.4,3.2)	77.7 (51.7,115.4)	10 (6.8,14.4)	96.6 (67.3,139.4)	24.3 (8.9,42.5)	0.75 (0.74, 0.77)
Honduras	2.3 (1.5,3.3)	129.1 (84.1,188.3)	7.7 (5.2,11)	143.1 (97.7,204.7)	10.9 (-0.1,25.3)	0.36 (0.33, 0.39)
Mexico	54.8 (37.1,79.6)	150.3 (103.9,218.1)	151.5 (104.7,217.6)	139.5 (96.5,200.9)	-7.2 (-9.4, -4.6)	-0.26 (-0.29, -0.23)
Nicaragua	2.1 (1.4,3)	162.4 (110.4,235.4)	6.7 (4.8,9.6)	175.5 (122.7,248.8)	8.1 (-2,19.7)	0.27 (0.25, 0.29)
Panama	1.8 (1.3,2.6)	133.3 (92.2,189.2)	5.6 (4,7.7)	134 (94.9,188)	0.5 (-9.3,10.4)	0.02 (0.01, 0.03)
Venezuela (Bolivarian Republic of)	16.6 (11.1,23.9)	205.9 (139.3,292.7)	53.1 (36,77.1)	198.1 (134.1,286.9)	-3.8 (-13.5,6.5)	-0.11 (-0.25, 0.02)
Central Sub-Saharan Africa	28.2 (18.2,43.4)	150 (97.3,224.1)	66 (43.8,99)	147.6 (95.6,222.5)	-1.6 (-8.6,5.5)	-0.06 (-0.06, -0.05)
Angola	4.6 (3,6.9)	143.8 (93.8,215)	13.9 (9.1,20.9)	150.7 (98.3,225.9)	4.8 (-5.3,15.8)	0.16 (0.15, 0.17)
Central African Republic	1.2 (0.8,1.9)	132.3 (85.5,200.2)	2.1 (1.3,3.1)	124.4 (80.2,187.9)	-5.9 (-16.3,4.3)	-0.2 (-0.23, -0.18)
Congo	1.4 (0.9,2.2)	156.4 (100.9,234.5)	3.5 (2.2,5.2)	157.5 (100.4,237.3)	0.7 (-8.9,11.5)	0.03 (0.01, 0.04)
Democratic Republic of the Congo	20 (12.7,31)	152.2 (97.6,227.3)	44.4 (29.4,66.7)	146.9 (95.4,221.9)	-3.5 (-12.1,5.9)	-0.12 (-0.13, -0.12)
Equatorial Guinea	0.2 (0.1,0.4)	137.4 (89.4,206.6)	0.6 (0.4,0.9)	153 (98.6,233.6)	11.3 (-0.1,24.3)	0.38 (0.35, 0.41)
Gabon	0.8 (0.5,1.2)	158.8 (102,235.7)	1.5 (1,2.2)	159.8 (103.4,239.1)	0.6 (-8.1,10.1)	0.02 (-0.01, 0.05)
East Asia	3042.4 (2152.4,4183.5)	452.9 (326.2,616.5)	8080.4 (5821.1,11009.5)	426.1 (306.6,574.8)	-5.9 (-8.6, -2.8)	-0.21 (-0.26, -0.16)
China	2974.5 (2099.4,4091.3)	461 (331.7,627.5)	7903.5 (5679.1,10769)	433.5 (311.6,583)	-6 (-8.7, -2.9)	-0.22 (-0.26, -0.17)

Democratic People's Republic of Korea	28.2 (20.2,38.9)	230.8 (165.9,320.1)	64.5 (46.2,90.7)	220 (156.2,308.7)	-4.7 (-13.7,6.6)	-0.17 (-0.18, -0.16)
Taiwan (Province of China)	39.8 (28.4,56)	305.1 (217.7,415.3)	112.5 (83.6,150.7)	281.8 (209.4,374.7)	-7.7 (-21.1,7.9)	-0.28 (-0.33, -0.24)
Eastern Europe	146.9 (97.9,218.3)	56.1 (38.3,81.9)	198.9 (133.6,295.1)	56.5 (38.2,83)	0.7 (-2.5,3.4)	0.03 (-0.02, 0.08)
Belarus	3.7 (2.6,5.3)	29.5 (20.5,41.9)	4.5 (3.1,6.4)	28.4 (19.7,39.7)	-3.8 (-13.4,6.6)	-0.14 (-0.19, -0.08)
Estonia	4.2 (2.8,6.2)	208.4 (142.5,307.6)	5.6 (3.7,8.3)	189.6 (126.9,279.9)	-9 (-20,3.2)	-0.33 (-0.4, -0.26)
Latvia	2.5 (1.7,3.7)	69.2 (46.9,102)	2.9 (1.9,4.5)	64.7 (43.4,95.1)	-6.6 (-18.3,6.5)	-0.23 (-0.27, -0.19)
Lithuania	2.9 (2,4.2)	64.2 (44.4,92.3)	3.5 (2.3,5.2)	56.8 (38.9,83)	-11.5 (-21.3, -0.6)	-0.42 (-0.48, -0.36)
Republic of Moldova	3.8 (2.6,5.6)	98.8 (68.1,142.1)	5.6 (3.8,8.2)	97 (66,140.8)	-1.8 (-12.7,10.3)	-0.06 (-0.11, -0.02)
Russian Federation	121.6 (80,182.3)	73.1 (49.2,107.9)	167.6 (112.3,251.8)	70.5 (47.4,105.1)	-3.4 (-6.6, -0.7)	-0.11 (-0.18, -0.05)
Ukraine	8.2 (5.4,12.1)	12.3 (8.2,17.9)	9.2 (6,13.7)	11.9 (8,17.3)	-3.6 (-12.5,4.9)	-0.13 (-0.16, -0.1)
Eastern Sub-Saharan Africa	171 (117.5,234.5)	269.4 (184.8,372.8)	394.2 (272.5,537.5)	281.8 (193.7,387.8)	4.6 (1.6,7.6)	0.15 (0.13, 0.18)
Burundi	5.5 (3.6,7.9)	265.7 (177.4,378.8)	10.6 (7.2,14.8)	280.6 (187.4,401.4)	5.6 (-3.6,15.4)	0.18 (0.14, 0.22)
Comoros	0.6 (0.4,0.9)	301.5 (202.7,425.1)	1.4 (0.9,2)	305.1 (204,433.9)	1.2 (-8.3,11.6)	0.03 (0.01, 0.06)
Djibouti	0.3 (0.2,0.5)	299.9 (200.9,428.8)	1.6 (1.1,2.2)	319.3 (213.6,459.3)	6.5 (-2.3,16.6)	0.22 (0.2, 0.23)
Eritrea	1.7 (1.1,2.4)	219.5 (149.5,314.3)	5.3 (3.6,7.3)	242.3 (162.6,343.8)	10.4 (0.4,21.8)	0.34 (0.33, 0.35)
Ethiopia	38.3 (26.4,51.9)	235 (162.6,316.3)	99.1 (69.8,132.5)	273.6 (188.4,367.9)	16.4 (10,22.8)	0.52 (0.5, 0.55)
Kenya	23.4 (16.5,30.8)	328.1 (229.5,431.7)	57.9 (41.1,76.8)	310.5 (217.1,420.5)	-5.4 (-9.5, -0.7)	-0.24 (-0.37, -0.12)
Madagascar	12.6 (8.6,17.6)	280.4 (188.7,397.2)	25.8 (17.6,35.9)	287 (192.4,412.5)	2.4 (-6.2,11.6)	0.08 (0.05, 0.1)
Malawi	9.7 (6.5,13.7)	296.3 (200.9,419.7)	18.5 (12.2,26.2)	289.1 (190.9,417.2)	-2.4 (-10.7,7)	-0.08 (-0.12, -0.05)
Mozambique	12.2 (7.9,18)	237.5 (155.6,357.7)	24 (15.7,34.8)	254.1 (165.2,372.8)	7 (-3.5,17.3)	0.24 (0.22, 0.27)
Rwanda	5.9 (4,8.5)	236.6 (157.1,340.5)	13 (8.7,18.4)	249.5 (166.8,357.2)	5.5 (-3.6,16.9)	0.18 (0.15, 0.21)
Somalia	5 (3.4,6.9)	249.6 (167.4,356.8)	13.5 (9,19.2)	248.7 (167.5,359.6)	-0.4 (-9.8,9.5)	-0.02 (-0.09, 0.05)
South Sudan	6 (4,8.5)	279 (186.5,396.9)	9.6 (6.6,13.2)	287.1 (193.8,403.9)	2.9 (-6.3,12.8)	0.1 (0.08, 0.12)
Uganda	16 (10.7,22.3)	282.2 (191.8,399.9)	33.3 (23.1,45.7)	274.4 (189.5,380.7)	-2.8 (-11.6,6.9)	-0.1 (-0.14, -0.05)
United Republic of Tanzania	27 (18.1,38)	283.2 (187.5,395.9)	64.9 (44.5,90.6)	299.2 (201.9,421.5)	5.6 (-3.9,15.3)	0.19 (0.17, 0.2)

Zambia	6.5 (4.4,9.3)	271.9 (180.2,393.2)	15.5 (10.4,21.8)	275.4 (183.4,392.7)	1.3 (-7.9,11)	0.05 (0.04, 0.06)
High-income Asia Pacific	166.8 (114.3,231.9)	94.2 (65.1,131.6)	434.6 (304.5,608.3)	85.1 (61.5,114.6)	-9.7 (-21.8,5)	-0.34 (-0.37, -0.32)
Brunei Darussalam	0.1 (0.1,0.1)	109.3 (75.3,154.9)	0.2 (0.1,0.3)	85.8 (56.6,123.9)	-21.5 (-31.8, -11.5)	-0.83 (-0.87, -0.79)
Japan	136.2 (91.9,193.3)	89.8 (61.2,126.9)	332.4 (228.4,475.4)	78.4 (55.7,106.8)	-12.7 (-26.9,5.5)	-0.46 (-0.5, -0.43)
Republic of Korea	27.8 (19.7,38.1)	120.7 (85.4,167.1)	94 (65.8,128.8)	111.6 (78,153)	-7.5 (-16.5,3)	-0.28 (-0.33, -0.22)
Singapore	2.8 (2,3.7)	140.6 (103.3,191.3)	8 (5.8,11)	106.5 (76.7,146.2)	-24.3 (-30.5, -17.4)	-0.94 (-1.06, -0.82)
High-income North America	843.3 (603,1130.9)	245.5 (178.2,325.2)	1540.5 (1125.4,2037.7)	253.1 (188.8,330.6)	3.1 (-9.7,18)	0.09 (-0.02, 0.2)
Canada	39.4 (29.4,51.7)	123.4 (93.1,159.8)	52.3 (39,67.8)	78.9 (59.2,102.8)	-36 (-41.9, -29.4)	-1.53 (-1.56, -1.5)
Greenland	0.1 (0,0.1)	195.6 (142.9,264)	0.1 (0.1,0.1)	133.4 (97.9,178.6)	-31.8 (-38.2, -25.2)	-1.31 (-1.34, -1.28)
United States of America	803.8 (577.2,1075)	258.3 (187,343.2)	1488.1 (1084.3,1971.7)	274.3 (203.7,359.4)	6.2 (-7.6,22.1)	0.19 (0.08, 0.29)
North Africa and Middle East	505 (359.7,689.9)	325 (237.4,443.5)	1331.8 (975.3,1764.5)	333.9 (249.1,446.5)	2.7 (-2.2,8.3)	0.09 (0.06, 0.11)
Afghanistan	21.3 (15,29.4)	333.3 (243,456.3)	39.6 (28.8,53.7)	357.6 (261.6,491.3)	7.3 (-2.9,19.1)	0.24 (0.21, 0.27)
Algeria	42.1 (29.6,58.4)	371.8 (270.1,508.7)	119.8 (86.5,165.7)	374.7 (270.4,512.6)	0.8 (-7.2,9.8)	0.03 (0.01, 0.04)
Bahrain	0.3 (0.2,0.4)	158.4 (115.7,218.3)	1.5 (1.1,2.1)	145.3 (105.2,197.9)	-8.3 (-15.6, -0.3)	-0.29 (-0.32, -0.26)
Egypt	71.4 (45.9,105.3)	270.7 (177.2,399.7)	162.5 (104.4,243.8)	283.1 (183.8,416.5)	4.6 (-5.3,14.5)	0.15 (0.14, 0.16)
Iran (Islamic Republic of)	98.9 (70.7,136)	420.1 (303,566.2)	285.1 (206.7,380.5)	406.3 (293.7,546.3)	-3.3 (-5.3, -1.3)	-0.12 (-0.14, -0.09)
Iraq	18.2 (13.2,25)	252.5 (184.7,351.6)	51.9 (37,71.8)	249.8 (179.6,349.7)	-1.1 (-9.9,8.3)	-0.04 (-0.05, -0.02)
Jordan	6.4 (4.7,8.5)	536.7 (395.1,710.9)	33.8 (25,44.7)	561.6 (417.9,747.6)	4.6 (-4.3,15.4)	0.15 (0.13, 0.17)
Kuwait	2.9 (2.2,3.8)	512.2 (373.8,682.8)	13 (9.5,17)	514.9 (376.3,690.6)	0.5 (-7.8,9.8)	0.02 (0, 0.03)
Lebanon	8.7 (6.3,11.9)	410.5 (300.1,556.5)	22.3 (16.1,30.9)	428.8 (312,592.5)	4.5 (-3.1,14.1)	0.15 (0.14, 0.16)
Libya	6.5 (4.8,8.6)	370.8 (272.5,501.2)	18.9 (13.9,25.3)	389.5 (282.7,534.1)	5.1 (-4.3,15)	0.17 (0.15, 0.19)
Morocco	44.3 (31.8,60.2)	353 (255.5,483.2)	103.5 (74.3,142.7)	355.9 (258.7,487.7)	0.8 (-8.1,10.3)	0.03 (0, 0.06)
Oman	1.2 (0.9,1.6)	183.7 (141.5,241.6)	4 (2.8,5.3)	223.1 (162.4,303.1)	21.4 (6.1,40.1)	0.67 (0.61, 0.73)
Palestine	2.7 (2,3.7)	336.9 (245.9,464.8)	7.3 (5.2,9.9)	337 (243.8,466.3)	0 (-9.3,9.9)	0 (-0.02, 0.02)
Qatar	0.2 (0.1,0.2)	116.6 (89.2,152)	1.3 (0.9,1.8)	105.2 (76.2,143.1)	-9.7 (-22.7,5.8)	-0.35 (-0.41, -0.29)
Saudi Arabia	5 (3.6,6.7)	87.7 (63.4,119.7)	17 (11.9,23.3)	94.9 (69.5,129)	8.1 (-1.1,19.4)	0.27 (0.25, 0.3)

Sudan	28.3 (20.3,39.6)	330.6 (239.6,456.9)	62.9 (45.4,85.5)	364.6 (263.6,500)	10.3 (0.2,21.7)	0.34 (0.28, 0.4)
Syrian Arab Republic	5.1 (3.6,7)	106.1 (76.3,145.1)	12.9 (9.1,17.9)	112.3 (81.1,156.4)	5.8 (-2.5,15.4)	0.2 (0.19, 0.2)
Tunisia	17.5 (12.3,23.9)	371.8 (267.3,501.6)	46.3 (33.7,62.5)	378.2 (277.5,513.6)	1.7 (-7.4,11.3)	0.06 (0.01, 0.1)
Turkey	106.6 (78.7,144.9)	327.7 (242.1,450.8)	263.3 (201.4,340.6)	313.1 (236.5,406.3)	-4.5 (-20.6,16.9)	-0.17 (-0.26, -0.09)
United Arab Emirates	1.9 (1.4,2.5)	403.1 (292.2,547.1)	18.4 (12.8,25.8)	424 (305.2,577.3)	5.2 (-4.5,17.3)	0.17 (0.14, 0.2)
Yemen	15.3 (11,21.3)	351.7 (258.8,479.7)	45.3 (32.5,61.5)	375.9 (274.8,511.6)	6.9 (-3.3,18.7)	0.23 (0.21, 0.25)
Oceania	7.8 (5.5,10.5)	341 (241.6,471.3)	18.8 (13.4,25.7)	344.9 (248.5,477.9)	1.1 (-4.8,7.8)	0.04 (0.03, 0.05)
American Samoa	0.1 (0,0.1)	276.1 (197.9,384.5)	0.1 (0.1,0.2)	274 (198.2,376.7)	-0.7 (-10,8.2)	-0.02 (-0.04, 0)
Cook Islands	0.1 (0.1,0.1)	717.4 (544.6,928.2)	0.2 (0.1,0.2)	703.1 (532.9,920.7)	-2 (-9.9,6)	-0.07 (-0.08, -0.06)
Fiji	1.1 (0.8,1.5)	388.5 (277.3,539.1)	2.3 (1.6,3.1)	383.6 (275.9,529.3)	-1.2 (-10.5,8.2)	-0.03 (-0.08, 0.01)
Guam	0.3 (0.2,0.4)	428.3 (309.3,599)	0.8 (0.6,1.1)	443.9 (320.4,622.7)	3.6 (-5.3,12.9)	0.12 (0.08, 0.16)
Kiribati	0.1 (0,0.1)	229.3 (163,326.7)	0.1 (0.1,0.2)	238.6 (169,336.9)	4.1 (-6,14.6)	0.15 (0.12, 0.17)
Marshall Islands	0 (0,0.1)	351.6 (250.3,490)	0.1 (0.1,0.1)	357.8 (256.7,502.2)	1.8 (-7.7,11.9)	0.05 (0.01, 0.09)
Micronesia (Federated States of)	0.1 (0.1,0.2)	364.8 (265,510.9)	0.2 (0.1,0.3)	363.3 (261.3,497.9)	-0.4 (-9.8,9.9)	-0.02 (-0.05, 0)
Nauru	0 (0,0)	331.3 (233.1,468.9)	0 (0,0)	328.9 (236,464.5)	-0.7 (-9.9,9.2)	-0.02 (-0.03, -0.01)
Niue	0 (0,0)	381.1 (273,530.2)	0 (0,0)	382.4 (276.5,536.4)	0.3 (-8.3,10)	0.01 (-0.01, 0.04)
Northern Mariana Islands	0 (0,0)	201.7 (145.9,284.9)	0.1 (0.1,0.1)	208.6 (148.2,292.7)	3.4 (-5.3,12.6)	0.12 (0.1, 0.15)
Palau	0 (0,0)	80.3 (57.9,111.4)	0 (0,0)	79.4 (57,110.9)	-1.2 (-10.1,9)	-0.04 (-0.07, -0.01)
Papua New Guinea	4.7 (3.2,6.4)	332 (232.7,462)	12.2 (8.6,16.5)	339.7 (243.4,475.7)	2.3 (-7.1,13.3)	0.08 (0.07, 0.09)
Samoa	0.3 (0.2,0.4)	407.2 (292.8,564.3)	0.5 (0.4,0.7)	398.8 (287.5,554.5)	-2 (-9.9,7.1)	-0.07 (-0.08, -0.05)
Solomon Islands	0.3 (0.2,0.4)	264.8 (187.9,375.7)	0.6 (0.4,0.8)	267.6 (188.9,379.9)	1 (-9.1,12.2)	0.03 (0.02, 0.05)
Tokelau	0 (0,0)	381.4 (273.9,537.3)	0 (0,0)	398.5 (291.5,548.7)	4.5 (-4.7,15.9)	0.15 (0.14, 0.16)
Tonga	0.1 (0.1,0.2)	257.3 (184.8,356.9)	0.2 (0.1,0.3)	255 (181.9,355.7)	-0.9 (-8.9,8)	-0.03 (-0.04, -0.02)
Tuvalu	0 (0,0)	352.6 (253.2,491.4)	0 (0,0)	365.3 (263,508.3)	3.6 (-5.9,13.5)	0.12 (0.11, 0.14)
Vanuatu	0.2 (0.1,0.3)	347.2 (247.2,482.8)	0.5 (0.3,0.7)	336.9 (242.7,473)	-3 (-11.5,6.7)	-0.11 (-0.14, -0.07)
South Asia	432.6 (302.8,619.9)	110.2 (77.4,156.8)	1305.1 (917.1,1870.8)	111.8 (78.3,159)	1.5 (-0.6,3.5)	0.05 (0.04, 0.06)

Bangladesh	44.1 (31.4,62.6)	117.4 (84,164.6)	144.5 (101.8,203.1)	126.4 (90,175.2)	7.7 (-2.7,19.2)	0.25 (0.25, 0.26)
Bhutan	0.2 (0.1,0.2)	86.7 (62.2,121.7)	0.4 (0.3,0.6)	90.1 (63.5,125.8)	3.9 (-6.2,14.9)	0.13 (0.12, 0.14)
India	319.1 (220.3,462.7)	104 (72.2,148.7)	1035.6 (720,1489)	108.6 (75.5,155.2)	4.4 (2.4,6.4)	0.15 (0.13, 0.17)
Nepal	4.7 (3.4,6.7)	69.4 (48.9,97.9)	12.9 (9.1,18.5)	69.9 (49.5,98.3)	0.6 (-9.1,12.1)	0.02 (-0.01, 0.04)
Pakistan	64.6 (44.7,91.6)	133.5 (92.6,188.5)	111.7 (78.3,159.4)	138.6 (96.2,196.9)	3.8 (-2.3,9.8)	0.13 (0.11, 0.15)
Southeast Asia	737.4 (527.5,986.8)	335 (244.4,456.5)	1847.7 (1330.7,2473)	334.8 (244.8,451.6)	-0.1 (-3.3,3.4)	0 (-0.02, 0.02)
Cambodia	11.7 (8.2,16)	312.2 (222.2,430.1)	34.1 (24.1,47.1)	328 (231.9,452.5)	5.1 (-4.7,15.4)	0.17 (0.15, 0.19)
Indonesia	344.2 (244.7,463)	423.2 (305.1,573.1)	837.6 (596.6,1138.4)	451.9 (322.9,611)	6.8 (3.4,10.3)	0.22 (0.21, 0.24)
Lao People's Democratic Republic	4.7 (3.2,6.6)	264.4 (186.4,367.8)	10.6 (7.5,14.6)	283.9 (203.3,396.6)	7.4 (-3.1,20.3)	0.25 (0.23, 0.26)
Malaysia	7.4 (5.4,10.1)	90.3 (65.5,123.1)	23 (16.6,31.4)	95 (69.8,130.4)	5.2 (-4.6,14.9)	0.17 (0.15, 0.2)
Maldives	0.1 (0.1,0.1)	149.1 (107.1,205)	0.4 (0.3,0.6)	157.3 (112,219.2)	5.6 (-4.7,15)	0.18 (0.17, 0.2)
Mauritius	2.5 (1.8,3.5)	390.1 (280.3,540.1)	6.1 (4.4,8.5)	371.1 (270,519.5)	-4.9 (-13.5,5.5)	-0.17 (-0.19, -0.15)
Myanmar	62.8 (44.2,88.1)	316.6 (222.5,449.1)	131.9 (92.5,180.4)	319.2 (226.3,449.4)	0.8 (-9,11.8)	0.03 (0.02, 0.03)
Philippines	101.3 (72.5,134.4)	392.2 (283.8,523.2)	284.6 (203.8,378.8)	415.9 (302,553.8)	6 (4.5,7.6)	0.2 (0.17, 0.23)
Seychelles	0.3 (0.2,0.4)	466.6 (339,643.4)	0.5 (0.3,0.6)	469 (334.3,649.3)	0.5 (-8.2,10.9)	0.02 (0, 0.04)
Sri Lanka	29.5 (20.4,41.1)	313.1 (219.2,435.1)	75.6 (52.3,105.3)	312.5 (220.9,435.7)	-0.2 (-9.5,10.3)	-0.01 (-0.02, 0.01)
Thailand	35.1 (25.1,48.7)	113.8 (81.4,158.8)	117.4 (83.7,161.3)	117.2 (83.6,162.7)	2.9 (-6.3,13)	0.1 (0.09, 0.11)
Timor-Leste	0.7 (0.5,0.9)	295 (210.8,413.4)	2.4 (1.6,3.3)	320.6 (224.6,445.6)	8.7 (-2.2,19.7)	0.29 (0.27, 0.31)
Viet Nam	136.4 (96.9,187.3)	365.4 (261.5,511.9)	321.1 (240.3,420.5)	381.2 (283.6,512.5)	4.3 (-8.8,19.6)	0.14 (0.13, 0.16)
Southern Latin America	86.5 (58,127.9)	203.4 (136.8,295.5)	158.6 (108.7,229.2)	184 (127,265.4)	-9.5 (-17.2, -1.1)	-0.34 (-0.41, -0.26)
Uruguay	8.6 (5.9,12.4)	218.2 (152.4,309.7)	10.2 (7.1,14.3)	167.5 (118.3,233.6)	-23.2 (-30.3, -15.2)	-0.91 (-0.92, -0.89)
Argentina	53 (34.7,78.8)	177.3 (116.8,260.8)	89.5 (60.9,132.3)	159.6 (109.1,235)	-10 (-20.7,2.2)	-0.35 (-0.45, -0.26)
Chile	25 (16.7,36.2)	288.6 (194.7,412.9)	58.9 (40.3,83.4)	245.3 (167.6,346.7)	-15 (-24, -4.8)	-0.56 (-0.58, -0.54)
Southern Sub-Saharan Africa	40 (26.2,58.7)	162.8 (105.9,237.5)	74.2 (48.4,110.5)	147.8 (95.8,216)	-9.2 (-13.1, -5.4)	-0.33 (-0.35, -0.31)
Botswana	0.4 (0.3,0.7)	90.1 (57.6,135.9)	1.1 (0.7,1.6)	91.1 (58,140)	1.2 (-7.8,9.4)	0.04 (0.02, 0.05)
Eswatini	0.2 (0.1,0.3)	86.7 (54.9,130.8)	0.4 (0.3,0.7)	85.6 (54,130.4)	-1.2 (-11.3,8.6)	-0.05 (-0.07, -0.02)

Lesotho	0.7 (0.4,1.1)	77.7 (49,117.1)	0.8 (0.5,1.3)	77.9 (49.4,120.8)	0.3 (-9.2,10.7)	0.01 (0, 0.02)
Namibia	0.6 (0.4,0.9)	90 (57.1,137.7)	1.1 (0.7,1.7)	88.7 (56.6,135.8)	-1.5 (-11.4,8.7)	-0.05 (-0.06, -0.04)
South Africa	36 (23.5,53.3)	190 (122.5,278.2)	67.3 (43.8,100.1)	166.1 (108.1,241.9)	-12.6 (-16.4, -8.6)	-0.46 (-0.48, -0.44)
Zimbabwe	2 (1.4,3)	58.3 (41,84.5)	3.4 (2.2,5.3)	57.9 (36.3,87.7)	-0.6 (-19.7,23.4)	-0.02 (-0.03, -0.02)
Tropical Latin America	128.4 (87.3,183.8)	173.8 (120.6,246.8)	392.8 (272.3,557.7)	171 (118.4,242.8)	-1.6 (-5,2.4)	-0.05 (-0.1, 0)
Brazil	125.5 (85.5,180)	174.8 (120.9,248.5)	385.1 (265.8,547)	171.5 (118.6,244)	-1.8 (-5.2,2.2)	-0.06 (-0.11, -0.01)
Paraguay	2.9 (2,4.1)	143.3 (99.1,206.5)	7.7 (5.5,10.9)	149.8 (105.1,215.1)	4.5 (-5.9,16.5)	0.15 (0.14, 0.17)
Western Europe	738.8 (523.9,1040.8)	123.5 (89.5,169.7)	1298.3 (923.6,1798.4)	123.5 (89.2,169.2)	0.1 (-6.4,7.6)	0.02 (-0.03, 0.07)
Andorra	0.1 (0.1,0.1)	198.7 (140.4,277.4)	0.2 (0.2,0.3)	146.1 (102.9,206.6)	-26.5 (-33, -19.5)	-1.06 (-1.12, -0.99)
Austria	34.9 (25.4,47.4)	276.2 (205.6,368.2)	35.3 (25.9,47.2)	171.8 (127.2,227.3)	-37.8 (-43.6, -30.7)	-1.65 (-1.79, -1.51)
Belgium	14.4 (10,20.7)	90.6 (64.5,127.6)	18.4 (14,24.7)	70.7 (53.9,93.7)	-22 (-36.1, -4.5)	-0.86 (-0.89, -0.83)
Cyprus	0.7 (0.5,1)	94.7 (65.2,134.7)	1.4 (1,2)	73.6 (53.6,100.9)	-22.3 (-33.6, -6.8)	-0.84 (-0.98, -0.69)
Denmark	3.9 (2.8,5.2)	45.3 (33.7,60)	3.8 (2.7,5.3)	30.6 (22.5,41.7)	-32.4 (-37.8, -26)	-1.33 (-1.42, -1.24)
Finland	8 (6,10.5)	112.7 (84.9,146.3)	14.9 (11.1,19.9)	107 (80.7,140.4)	-5.1 (-15.6,6.5)	-0.17 (-0.24, -0.1)
France	110.4 (77.5,149.3)	125 (89.3,168.3)	125.4 (90,171.9)	77 (55.6,105.1)	-38.4 (-44.7, -31.5)	-1.66 (-1.71, -1.61)
Germany	185.3 (131.9,254.5)	141.3 (102.3,192.2)	344.5 (241.2,476.4)	160.3 (114.6,215.7)	13.4 (1,27.9)	0.41 (0.23, 0.59)
Greece	19.6 (13.6,28.2)	128.7 (90.2,181.6)	27.9 (19.5,39.6)	98 (70.3,138.9)	-23.9 (-30.4, -16.3)	-0.93 (-1, -0.86)
Iceland	0.3 (0.2,0.4)	85.7 (60.9,119.2)	0.4 (0.3,0.6)	69.5 (50.9,94.7)	-18.9 (-29.1, -5.4)	-0.7 (-0.77, -0.63)
Ireland	2.3 (1.7,3.3)	57.6 (40.8,79.8)	3.1 (2.2,4.4)	40.2 (28.5,56.4)	-30.3 (-36.6, -23.5)	-1.24 (-1.29, -1.18)
Israel	2.7 (1.9,3.7)	56.1 (40.4,76.7)	5.1 (3.7,7.1)	41.9 (30,57.2)	-25.3 (-31.4, -18.7)	-1 (-1.05, -0.95)
Italy	169.8 (105.3,258.5)	190.8 (123.4,282.4)	466.7 (314.6,664.2)	272.5 (188.1,386.7)	42.9 (24.4,71.8)	1.35 (0.98, 1.73)
Luxembourg	0.7 (0.5,1)	120.8 (85.3,170.1)	1.3 (1,1.8)	121.1 (88.3,161.2)	0.2 (-17.3,23.6)	-0.04 (-0.17, 0.09)
Malta	0.5 (0.4,0.7)	122.2 (86.8,170.8)	1.2 (0.8,1.7)	119.1 (84.8,166.1)	-2.6 (-15.1,14.9)	-0.08 (-0.15, 0)
Monaco	0.1 (0.1,0.1)	105.7 (75,146.2)	0.1 (0.1,0.1)	74.6 (52.3,104.4)	-29.5 (-36, -22.5)	-1.19 (-1.26, -1.12)
Netherlands	9.6 (7.2,12.7)	46.7 (35.3,61.4)	14 (10,19.2)	38.2 (27.4,51.9)	-18.2 (-31.9, -3.6)	-0.69 (-0.72, -0.67)
Norway	4.6 (3,6.8)	61.3 (41.5,89.6)	6.3 (4.4,8.9)	60.3 (41.9,84.8)	-1.7 (-12.5,13)	-0.07 (-0.08, -0.05)

Portugal	15.4 (10.6,22.1)	113.7 (79.4,159.8)	20.7 (14.7,28.8)	72.4 (51.7,99.5)	-36.3 (-45.8, -25.8)	-1.56 (-1.67, -1.45)
San Marino	0 (0,0.1)	148.5 (105.8,209.3)	0.1 (0.1,0.1)	105.6 (75,151.4)	-28.9 (-35.2, -21.1)	-1.17 (-1.22, -1.11)
Spain	57.4 (41,80.9)	104.1 (74.7,142.2)	81.5 (56.6,116.4)	72.1 (51.1,102.3)	-30.8 (-40.1, -20.8)	-1.28 (-1.33, -1.22)
Sweden	22.5 (15.2,32.6)	139.7 (96.6,196.1)	37.3 (25.2,54)	150.1 (103.6,214)	7.5 (-3.1,19.6)	0.3 (0.13, 0.46)
Switzerland	10.9 (7.6,15.5)	95.8 (68.5,133.5)	16.7 (12.2,22.8)	82.3 (61.4,110.8)	-14.1 (-28.8,5.5)	-0.51 (-0.63, -0.38)
United Kingdom	64.3 (43.4,94.8)	68.1 (47.6,97.9)	70.7 (49.5,99.5)	52.6 (37.6,72.5)	-22.8 (-30.1, -13.8)	-0.9 (-1.06, -0.74)
Western Sub-Saharan Africa	171.4 (121.2,233.7)	229.9 (161.2,310)	381.3 (267.1,513.6)	242.9 (168.7,330.3)	5.6 (2.2,8.9)	0.19 (0.15, 0.23)
Benin	3.8 (2.5,5.6)	211.3 (137.5,309.6)	9 (6,12.8)	215.7 (142.1,313.4)	2.1 (-6.2,11)	0.07 (0.06, 0.08)
Burkina Faso	7.2 (4.7,10.8)	198.2 (129.9,288.2)	15.2 (10.1,21.8)	198.7 (131.1,290.2)	0.3 (-8.3,10)	0.01 (-0.02, 0.03)
Cabo Verde	0.6 (0.4,0.9)	257.1 (168.3,377)	1.1 (0.7,1.5)	265.6 (173.4,390.2)	3.3 (-5.5,12.8)	0.11 (0.09, 0.12)
Cameroon	4.7 (3.3,7)	134.5 (90.5,198.5)	18.5 (12.2,27)	189.1 (122.6,281.5)	40.6 (18.6,69.2)	1.15 (0.92, 1.37)
Chad	4.9 (3.1,7.3)	192.1 (125.1,284.9)	9.6 (6.3,14)	204.5 (133,301.2)	6.4 (-3.5,17.9)	0.2 (0.16, 0.24)
CÃ´te d'Ivoire	6.8 (4.4,9.9)	223.3 (144.5,331.5)	19.1 (12.7,27.7)	225.6 (145.1,336.2)	1 (-7,9.7)	0.03 (0.02, 0.04)
Gambia	0.7 (0.5,1)	237.7 (153.8,351.3)	2.1 (1.4,3)	244.8 (159,356.7)	3 (-5.6,13.1)	0.1 (0.07, 0.13)
Ghana	5.7 (3.9,8.1)	112.1 (76.8,162.6)	16.7 (10.8,25.2)	123.6 (78.5,185)	10.3 (-8.7,34.1)	0.34 (0.29, 0.4)
Guinea	6.5 (4.6,9.1)	221.3 (156.6,304.8)	11.8 (8.1,16.6)	243.1 (163.6,343.7)	9.9 (-4.6,23.7)	0.32 (0.3, 0.35)
Guinea-Bissau	0.6 (0.4,0.9)	195 (126.8,288.7)	1.2 (0.8,1.7)	208 (136.4,305.2)	6.7 (-2.9,17.5)	0.22 (0.2, 0.23)
Liberia	2.2 (1.4,3.3)	225.3 (146.6,327)	4.1 (2.8,5.9)	241.4 (156.4,352.6)	7.2 (-2.1,17.7)	0.23 (0.19, 0.28)
Mali	6.8 (4.4,10.2)	198 (129.1,288.1)	15.5 (10.2,22.6)	212.5 (138.4,311)	7.3 (-2,17.6)	0.24 (0.23, 0.25)
Mauritania	2.1 (1.3,3.1)	233.5 (150.2,342.6)	4.7 (3.1,6.9)	251.2 (165.4,367.3)	7.6 (-2.2,18.1)	0.25 (0.23, 0.26)
Niger	4.3 (2.8,6.3)	193.2 (124.9,283.6)	13.1 (8.6,19.1)	207.6 (135.9,300.5)	7.5 (-2.8,18.2)	0.25 (0.23, 0.26)
Nigeria	100.6 (71.9,131)	264.8 (190.1,342.9)	208.5 (152,268.9)	288 (205.8,375.3)	8.7 (4.8,13.2)	0.29 (0.22, 0.37)
Sao Tome and Principe	0.1 (0.1,0.2)	229.7 (149.7,336.8)	0.2 (0.1,0.3)	246.4 (163.7,358.9)	7.3 (-1.8,17.1)	0.24 (0.23, 0.25)
Senegal	7.2 (4.8,10.3)	257.6 (170.7,367.9)	16.9 (11.3,24.1)	257.9 (170,372)	0.1 (-8.2,8.9)	-0.01 (-0.05, 0.03)
Sierra Leone	4 (2.6,6)	233.9 (151.1,343.4)	7.3 (4.8,10.6)	237.7 (156.7,347.6)	1.6 (-7.8,11.1)	0.06 (0.04, 0.08)
Togo	2.3 (1.5,3.4)	226.9 (148.5,337.3)	6.7 (4.4,9.7)	226.6 (148.7,331.9)	-0.1 (-8.8,8.9)	-0.01 (-0.03, 0.01)

Table S4. DALYs due to hypertensive heart disease in 1990 and 2019, percentage change in the age-standardized rates (ASRs) per 100,000, and their average annual percentage changes (AAPCs) from 1990 to 2019 by location.

	1990		2019		Percentage change in ASRs from 1990 to 2019	AAPC (95% CI)
	No. in thousands (95% UI)	ASRs per 100000 (95% UI)	No. in thousands (95% UI)	ASRs per 100000 (95% UI)		
Global	13943.6 (11312.1,15650.1)	364.6 (297.7,406.7)	21508 (16400.1,23899.9)	268.2 (204.6,298.1)	-26.4 (-35.5, -15.7)	-1.08 (-1.23, -0.93)
Andean Latin America	47.2 (40.5,53.9)	232.3 (199.5,264.9)	90.9 (74.6,108.3)	164.6 (134.9,195.7)	-29.1 (-43.2, -13.3)	-1.23 (-1.47, -0.99)
Bolivia (Plurinational State of)	11.7 (7,15.8)	380 (233.6,512.8)	23.9 (16.6,33)	293.3 (206.4,400.1)	-22.8 (-46.4,10.5)	-0.9 (-0.98, -0.83)
Ecuador	19.9 (18.2,21.9)	382.4 (348.8,418.8)	45.1 (35.1,56)	318.9 (248.5,393)	-16.6 (-35.8,3.9)	-0.72 (-1.25, -0.18)
Peru	15.7 (13,18.3)	130 (108.6,152.9)	21.9 (16.5,29.4)	67.3 (50.9,90)	-48.2 (-62.3, -26.9)	-2.14 (-2.65, -1.62)
Australasia	13.7 (10.8,15.3)	60.5 (47.9,67.4)	20.4 (16.9,24.2)	38.9 (32.9,46.6)	-35.6 (-41, -9.9)	-1.51 (-1.65, -1.37)
Australia	10.7 (8.7,12.1)	57.2 (46.6,64.7)	17 (13.9,20)	38.4 (32,45.2)	-32.8 (-39.5, -10.6)	-1.35 (-1.47, -1.22)
New Zealand	2.9 (2.1,3.3)	77.3 (55.5,85.7)	3.4 (2.9,4.6)	41.9 (35.9,56.1)	-45.8 (-53.1, -7.2)	-2.11 (-2.48, -1.75)
Caribbean	100.7 (86.7,120.6)	388.8 (336.2,467.5)	192.4 (158.2,229.2)	372.3 (306.2,444.1)	-4.2 (-20.1,12.6)	-0.1 (-0.22, 0.01)
Antigua and Barbuda	0.3 (0.3,0.3)	544.4 (471.3,609.8)	0.5 (0.4,0.6)	537 (425.2,637.9)	-1.4 (-17.5,17.1)	0.13 (-0.26, 0.52)
Bahamas	1.7 (1.5,2)	1094.3 (966.5,1282.9)	4.3 (3.4,5.4)	1089.4 (866.6,1363.3)	-0.4 (-20.3,24.6)	-0.06 (-0.56, 0.45)
Barbados	0.8 (0.7,0.9)	285.6 (245.8,322.5)	1.3 (1,1.6)	260 (210.1,323)	-9 (-25,10.8)	-0.36 (-0.66, -0.06)
Belize	0.3 (0.3,0.4)	360.2 (314.5,435)	1.1 (0.9,1.3)	393.4 (321.9,463.7)	9.2 (-9.8,30.9)	0.33 (0.04, 0.62)
Bermuda	0.1 (0.1,0.1)	183.4 (141.3,207.1)	0.2 (0.1,0.2)	125.5 (96.9,156.1)	-31.6 (-47.7, -9.8)	-1.27 (-1.6, -0.94)
Cuba	12.1 (11,16.1)	118.7 (107.2,158.1)	39.9 (21.6,50.2)	209.3 (113.9,263.5)	76.4 (-17.2,128.3)	2.1 (1.78, 2.41)
Dominica	0.7 (0.6,0.8)	958.1 (844.8,1078.1)	0.7 (0.6,0.9)	817.5 (667.4,1013.1)	-14.7 (-32.1,8.6)	-0.57 (-0.89, -0.24)
Dominican Republic	13.7 (12,15.9)	377.4 (329.8,436.6)	36.7 (26.3,49.4)	401.7 (293.2,536.5)	6.4 (-25.4,44.9)	0.23 (-0.15, 0.61)
Grenada	0.4 (0.3,0.5)	537.6 (458.4,642.5)	0.5 (0.4,0.6)	491.1 (394.8,564.8)	-8.6 (-25.3,7.5)	-0.24 (-0.59, 0.1)
Guyana	6.1 (4.6,7)	1610.6 (1207.2,1843.6)	7.3 (5.5,9.5)	1157.4 (894.9,1490)	-28.1 (-46.2, -3.3)	-1.13 (-1.37, -0.9)

Haiti	30.7 (19.9,49)	950.9 (628.6,1653.7)	52.9 (31,82.8)	762.5 (450.2,1181)	-19.8 (-44.5,15.2)	-0.73 (-0.86, -0.61)
Jamaica	13 (8.2,14.3)	721 (461.2,791.3)	13.7 (10.9,18)	451.1 (357.4,594.8)	-37.4 (-52.4,7.7)	-1.38 (-4.67, 2.01)
Puerto Rico	8.3 (7.5,10.4)	232.6 (210.5,288)	15.4 (11.6,19.7)	229.5 (168.4,294.7)	-1.3 (-30.1,27.2)	-0.08 (-0.56, 0.4)
Saint Kitts and Nevis	0.2 (0.2,0.2)	582.2 (466,661.7)	0.3 (0.2,0.4)	466.3 (371.5,571.7)	-19.9 (-37,2)	-0.72 (-1.23, -0.2)
Saint Lucia	0.5 (0.4,0.6)	609.9 (509.3,683.8)	0.9 (0.7,1.1)	420.4 (346.5,511.5)	-31.1 (-42.7, -13.4)	-1.27 (-1.68, -0.86)
Saint Vincent and the Grenadines	0.5 (0.4,0.6)	705.5 (615.5,813.9)	0.9 (0.7,1)	670.5 (557.4,788.3)	-5 (-19.4,11.7)	-0.16 (-0.33, 0.01)
Suriname	1.4 (1.2,1.5)	534.8 (467.3,590.5)	2.6 (2.1,3.1)	437 (351.5,530.3)	-18.3 (-34.1,1.9)	-0.58 (-1.09, -0.06)
Trinidad and Tobago	6 (4.3,6.5)	736.9 (530.8,800.6)	5.9 (4.3,9.8)	323.2 (236.8,539.1)	-56.1 (-68.4, -13)	-2.76 (-3.12, -2.39)
United States Virgin Islands	0.4 (0.3,0.5)	493.9 (405.2,611.6)	0.9 (0.7,1)	489.1 (407.8,575)	-1 (-24.7,27.4)	0.08 (-0.14, 0.3)
Central Asia	147.4 (131.1,191.1)	325.3 (288.5,423.3)	292.5 (252.6,335)	454.6 (390,515.3)	39.7 (8.2,68.9)	1.22 (0.95, 1.48)
Armenia	5.1 (3.2,6.3)	229.2 (125.4,287.1)	9.4 (5.1,11.2)	233.4 (123.4,280)	1.9 (-19.1,28.9)	0.09 (-0.22, 0.41)
Azerbaijan	25.5 (21.1,32.6)	533.7 (439,692)	39.3 (29.1,52.6)	510.5 (373.7,682.3)	-4.4 (-42.7,36.7)	-0.06 (-0.48, 0.37)
Georgia	15.2 (11.3,32.3)	265 (197.8,541.5)	54.5 (28.1,65.6)	860.1 (465,1036.7)	224.6 (2.2,370.6)	4.12 (3.7, 4.54)
Kazakhstan	36.4 (30.5,57.9)	285.9 (238.9,480.8)	46.7 (36.9,87.5)	275.4 (217.9,531.3)	-3.7 (-21.9,21.1)	0.03 (-0.84, 0.91)
Kyrgyzstan	6.9 (5.7,8.6)	229.1 (191.8,286.6)	11.1 (8.3,13.3)	252.2 (193.6,299.7)	10.1 (-14.5,35.3)	0.35 (0.01, 0.69)
Mongolia	5 (2.8,6.2)	484.7 (279.2,593.1)	4.8 (3.5,6.2)	211 (161.8,267.9)	-56.5 (-67.9, -28.8)	-2.81 (-3.02, -2.6)
Tajikistan	19.1 (13.6,25.4)	695.7 (489.6,943.2)	35.5 (23,44.5)	873.6 (556.4,1085.4)	25.6 (-7.4,64.4)	0.83 (0.51, 1.15)
Turkmenistan	5.2 (4.5,6.3)	282.6 (242.6,341.1)	12 (9.2,15.5)	324.7 (250.5,415.8)	14.9 (-12.4,49.1)	0.49 (0.19, 0.79)
Uzbekistan	29 (23.6,36.1)	262.3 (213.7,327.4)	79.3 (55.9,102.7)	462.2 (311.8,622.6)	76.2 (13.3,168.6)	1.99 (1.78, 2.2)
Central Europe	466.5 (437.9,562.1)	328.3 (306.6,398.8)	730.9 (546.3,846.7)	334.8 (249.4,387.4)	2 (-28.4,17.6)	0.06 (-0.02, 0.14)
Albania	3.2 (2.8,3.6)	174.3 (155.6,197.4)	4.2 (3.2,5.6)	99.3 (74.6,130.8)	-43 (-57.9, -23.1)	-1.97 (-2.41, -1.53)
Bosnia and Herzegovina	7.9 (7.2,9.4)	208.5 (188.9,264.8)	8.3 (6.5,12.5)	144.6 (114.1,213.7)	-30.7 (-45.2, -11.2)	-1.31 (-1.78, -0.85)
Bulgaria	67.6 (60,113)	569.7 (504.6,965)	178.2 (110.8,224.8)	1238.8 (758.5,1564.6)	117.4 (-8.5,195.1)	2.81 (2.55, 3.07)
Croatia	19.6 (14.5,21.5)	332 (240.2,364)	22.9 (16.2,28.6)	247 (178.2,309.9)	-25.6 (-40.9, -3.7)	-1.07 (-1.45, -0.69)
Czechia	10 (8.3,21.6)	74 (61.5,159.5)	25.1 (18.6,30.9)	119.8 (88.5,149.3)	61.8 (-41.4,121.7)	1.86 (1.17, 2.55)
Hungary	65.1 (57.6,74.4)	456 (400.9,522.8)	85.4 (59.1,105)	436 (302.5,538.3)	-4.4 (-34.4,16.6)	0.02 (-0.35, 0.39)

Montenegro	0.4 (0.4,0.5)	69.2 (60.3,82.5)	0.8 (0.6,1)	81.3 (65.1,99.5)	17.5 (-11,49.8)	0.74 (0.35, 1.14)
North Macedonia	9.8 (8.8,11.3)	634 (557.6,736.9)	15.1 (11.9,18.4)	561 (441,673.3)	-11.5 (-31.3,8.9)	-0.35 (-0.64, -0.05)
Poland	90.9 (83.1,110.3)	213.7 (196.8,262.3)	134.5 (105.3,163)	189.4 (147.2,229.9)	-11.4 (-32.7,6)	-0.48 (-0.62, -0.33)
Romania	145.3 (122,156.2)	553.4 (473.3,594.9)	183.4 (144.4,223.3)	469.8 (369.8,573)	-15.1 (-30.2,2.1)	-0.54 (-0.94, -0.15)
Serbia	32.2 (27.6,40.2)	329.7 (281.1,410.6)	43.8 (34.2,53.4)	290 (222.3,351.3)	-12 (-35.8,12.3)	-0.34 (-0.7, 0.01)
Slovakia	8.1 (7,14)	138.8 (118.5,238.4)	18.8 (13.5,23.7)	207.3 (147.7,260.5)	49.3 (-35.4,100.5)	1.44 (0.88, 2)
Slovenia	6.4 (4.4,8.1)	266 (181,339.8)	10.3 (5.6,13.2)	202.7 (120.3,262.4)	-23.8 (-48.6,4.4)	-0.99 (-1.2, -0.78)
Central Latin America	224.8 (161.1,238.5)	288.8 (208.2,307.2)	392.5 (329.2,503.1)	171.5 (144.2,219.6)	-40.6 (-50.1, -6.3)	-1.81 (-2.06, -1.56)
Colombia	74.2 (42.2,81.1)	461.3 (259.5,506.2)	82.3 (62.3,131)	152.8 (115.3,244.9)	-66.9 (-75.4, -11.8)	-3.78 (-4.15, -3.41)
Costa Rica	3.3 (2.7,3.6)	195.6 (160.1,218.3)	8.5 (6.7,10.8)	166.1 (131.1,211.6)	-15.1 (-32.7,7.7)	-0.56 (-0.99, -0.14)
El Salvador	4.6 (4.1,5.3)	151.6 (136.3,181.6)	6.7 (5.1,8.5)	109.3 (84,139.7)	-27.9 (-45.8, -6.2)	-1.16 (-1.4, -0.92)
Guatemala	6.7 (5.1,7.7)	189.5 (157.6,214.5)	12.4 (9.7,17.5)	117.3 (92,170.2)	-38.1 (-51.7, -4.7)	-1.59 (-1.96, -1.22)
Honduras	6.3 (5.1,8.8)	291.4 (234.7,444.5)	16.8 (11.4,26.3)	285.5 (192.5,464.2)	-2 (-35.2,30.5)	-0.03 (-0.43, 0.38)
Mexico	73 (55,78.1)	190.5 (140.9,204.3)	151 (126,177.6)	136.6 (113,160.3)	-28.3 (-37.7, -10.2)	-1.1 (-1.36, -0.85)
Nicaragua	3.9 (3.4,4.3)	271.4 (236.6,303.4)	11.1 (8.9,13.2)	286 (230.8,334.7)	5.4 (-14.3,25.3)	0.29 (-0.41, 1)
Panama	1.5 (1.3,2)	102.1 (89.1,138.1)	5.2 (3.9,6.7)	125.6 (93.9,159.5)	23.1 (-16.9,60)	0.69 (0.45, 0.93)
Venezuela (Bolivarian Republic of)	51.3 (37.2,55.2)	547.7 (406.1,591.8)	98.4 (73.6,136.3)	350.8 (263.9,486.7)	-35.9 (-52.4,1.5)	-1.58 (-2.28, -0.87)
Central Sub-Saharan Africa	250 (156.3,326)	1143.1 (733.8,1487.5)	479.6 (307.6,648.1)	970.3 (625.8,1310.4)	-15.1 (-33.3,7.3)	-0.56 (-0.65, -0.46)
Angola	43.3 (27.1,58.3)	1134.6 (722.6,1537)	82.1 (50.1,111)	799.7 (488,1071.5)	-29.5 (-49.3,0.5)	-1.2 (-1.39, -1)
Central African Republic	16.8 (10,22.8)	1474.7 (912.7,2015.2)	27.1 (15.6,38.2)	1321.8 (787.6,1924.4)	-10.4 (-33.8,23.1)	-0.39 (-0.5, -0.27)
Congo	15 (9,19.5)	1452.5 (903.4,1864.7)	24 (15.3,32.3)	984.8 (633.5,1307.6)	-32.2 (-49.7, -7.4)	-1.33 (-1.55, -1.11)
Democratic Republic of the Congo	166 (99,224.6)	1093.7 (675.4,1495.4)	335.1 (205.7,475)	1007.4 (627,1419.5)	-7.9 (-30.6,23.3)	-0.32 (-0.41, -0.23)
Equatorial Guinea	2.6 (1.6,3.6)	1357.7 (827.2,1887.5)	2.9 (1.7,4.4)	672.7 (387.7,958.5)	-50.5 (-69.5, -16.8)	-2.36 (-2.59, -2.13)
Gabon	6.3 (3.9,8.1)	1171.7 (729.9,1509.1)	8.4 (5.3,11.3)	867.6 (555.1,1137.8)	-26 (-44.6,1.1)	-1.01 (-1.22, -0.8)
East Asia	5144.4 (3759.9,5899.1)	680 (516,768.8)	5780.8 (4053.6,6713.5)	310.4 (217.5,359.4)	-54.4 (-63.6, -44.7)	-2.68 (-2.97, -2.38)
China	5042.2 (3661.3,5792.7)	692.6 (526.3,786)	5594.9 (3877.3,6533)	312.9 (214.1,363.7)	-54.8 (-64.4, -44.9)	-2.71 (-3.01, -2.41)

Democratic People's Republic of Korea	53.2 (35.9,75.5)	386.3 (264.2,537.8)	114.6 (89,146)	376.1 (293.9,479.9)	-2.6 (-30.3,38)	-0.08 (-0.14, -0.02)
Taiwan (Province of China)	49 (32.6,53.1)	358.1 (244,389.6)	71.4 (57,96)	179.7 (143.6,241.4)	-49.8 (-60.5, -14.2)	-2.34 (-2.67, -2)
Eastern Europe	225.5 (202.5,326.7)	82.7 (74,120)	447.2 (297.6,517.3)	131.7 (88.5,152.6)	59.3 (-6.5,85.1)	1.65 (0.9, 2.41)
Belarus	14 (8.4,17.8)	108.6 (65.8,138.2)	7.9 (5.7,13.6)	51.4 (37.2,86.4)	-52.7 (-67,10.1)	-2.62 (-3.13, -2.1)
Estonia	3.4 (2.7,7.3)	166.4 (134.9,360.5)	26.7 (7.1,35)	953.3 (273.9,1252.7)	473 (-17.1,748.4)	6.22 (5.29, 7.17)
Latvia	1.7 (1.3,4.1)	47.6 (37.4,117.2)	10.1 (3.2,13.1)	247.7 (84.2,321.6)	420.1 (-15.9,697.3)	5.72 (5.03, 6.41)
Lithuania	3.5 (3,6)	79.3 (66.4,135.2)	9 (5.2,11.3)	166.3 (94.4,212.6)	109.8 (-18.5,184.8)	2.62 (1.78, 3.46)
Republic of Moldova	4.2 (3.5,9)	98.3 (80.8,211.4)	25.3 (8.3,31.3)	432.7 (144.2,534.9)	340 (-24.3,502.8)	5.32 (4.72, 5.92)
Russian Federation	181.3 (163,262.5)	102.6 (92,150.3)	346.2 (234.4,407.6)	149.7 (102.7,176.5)	45.9 (-11.5,72.5)	1.26 (0.37, 2.16)
Ukraine	17.3 (14.2,28.4)	26.2 (21.5,40.8)	22.1 (18,33.3)	31.9 (26.2,44.9)	21.9 (-6,53.8)	0.68 (-0.2, 1.56)
Eastern Sub-Saharan Africa	752 (457.8,1090.5)	1013.4 (625.8,1450.8)	1077.4 (658.7,1587.1)	696.9 (418.4,1038.7)	-31.2 (-43.4, -9.5)	-1.28 (-1.35, -1.22)
Burundi	23.7 (4.3,41.6)	1011 (183.1,1779)	28 (12.2,50.7)	651.7 (282.8,1155.6)	-35.5 (-56.3,76.3)	-1.5 (-1.63, -1.38)
Comoros	2.1 (1,3.2)	986.4 (504.6,1475.2)	3.1 (1.8,4.8)	666.6 (393.8,1027)	-32.4 (-52.1,11.6)	-1.33 (-1.7, -0.96)
Djibouti	1.3 (0.8,2)	977.4 (598.7,1417)	3.5 (2.1,5.6)	650.4 (384.7,977.3)	-33.5 (-51.6, -5.3)	-1.39 (-1.53, -1.25)
Eritrea	13.4 (7.2,20.7)	1331.1 (734.1,2158)	24.8 (13.1,40.3)	998 (550.8,1610.2)	-25 (-50.5,10.1)	-1.02 (-1.14, -0.89)
Ethiopia	241.9 (125.5,369)	1204.1 (684.4,1795.4)	218.1 (126.1,355.9)	559.7 (322.5,920.4)	-53.5 (-66.8, -19.7)	-2.61 (-2.72, -2.5)
Kenya	47 (29.8,72.5)	595.2 (379,902.8)	113.8 (70.8,180)	564.7 (343.2,903.3)	-5.1 (-20.9,13.5)	-0.18 (-0.26, -0.11)
Madagascar	77.2 (47.6,107)	1433.8 (883.5,1987.1)	140.2 (82.6,206.2)	1258.8 (740.1,1886.4)	-12.2 (-34.6,18.9)	-0.42 (-0.57, -0.27)
Malawi	30.8 (18.1,45)	835.9 (501.1,1214.3)	44.4 (25.7,71.3)	653 (378,1037.4)	-21.9 (-41.5,10.6)	-0.86 (-0.95, -0.76)
Mozambique	53.8 (32.9,78.4)	962.8 (599.9,1418.9)	91.2 (55.2,135.5)	899.4 (543.1,1336.3)	-6.6 (-31.6,29.5)	-0.24 (-0.4, -0.07)
Rwanda	36.1 (10.9,58.1)	1242.6 (378.7,2090.5)	32.6 (10.4,58)	591.5 (185.1,1040)	-52.4 (-66.7, -26)	-2.56 (-2.85, -2.26)
Somalia	36.3 (19.9,52.5)	1386.3 (804.6,1956.1)	64.1 (38.7,96.9)	1012.3 (619.3,1508.1)	-27 (-47.2,3.9)	-1.08 (-1.16, -1)
South Sudan	20.9 (11.8,31.9)	899.3 (510.4,1353.8)	20.7 (11.9,35.7)	573.8 (335.6,989.7)	-36.2 (-54.4, -7.9)	-1.54 (-1.64, -1.44)
Uganda	42.6 (18.1,73.3)	686.6 (295.5,1167.8)	77.5 (30,132)	583.2 (222.5,995.7)	-15.1 (-34.7,8.2)	-0.58 (-0.73, -0.43)
United Republic of Tanzania	95.9 (58.5,138.8)	926.8 (568.8,1342.4)	149.7 (78.3,240.1)	652.3 (339,1044.3)	-29.6 (-46.5, -7.6)	-1.2 (-1.32, -1.07)

Zambia	28.3 (18.3,37.1)	1016.3 (668.5,1308.7)	65 (39.9,88)	1034.1 (635.3,1388.8)	1.8 (-26.3,41.5)	0.04 (-0.1, 0.19)
High-income Asia Pacific	234.5 (153.1,252.8)	129.2 (81.3,139.9)	231.3 (187.4,320.8)	44 (36.5,64.6)	-65.9 (-71, -28.6)	-3.65 (-3.8, -3.5)
Brunei Darussalam	0.4 (0.3,0.5)	445.3 (371.4,541.4)	0.6 (0.5,0.9)	275.3 (233.6,369.9)	-38.2 (-49.5, -22.3)	-1.67 (-1.97, -1.38)
Japan	155.7 (89.8,170.1)	101.5 (56.5,111.5)	153.5 (120.5,221)	34.7 (28.1,56.1)	-65.8 (-71.3, -9.8)	-3.7 (-3.88, -3.52)
Republic of Korea	73.4 (52.8,80)	277.6 (214.5,304.3)	62.7 (51.2,93.1)	77.4 (62.8,111.5)	-72.1 (-77.5, -52)	-4.31 (-4.59, -4.03)
Singapore	5 (4.1,5.6)	230.9 (199.4,266.9)	14.5 (9,16.7)	184.9 (119,212.4)	-19.9 (-55.1, -7.6)	-0.76 (-1.22, -0.29)
High-income North America	526 (451.7,581.9)	155.3 (133.2,171.8)	1102 (735.5,1201.1)	193.7 (126.5,210.2)	24.7 (-14.5,32.4)	0.76 (0.61, 0.91)
Canada	12.9 (11.4,15.6)	40.7 (35.7,48.8)	26.1 (18.7,29.7)	39.1 (29.3,44.4)	-3.9 (-33.6,7.7)	-0.13 (-0.37, 0.11)
Greenland	0.1 (0.1,0.1)	207 (173.7,251)	0.1 (0.1,0.1)	156.1 (123.8,193.8)	-24.6 (-43.3, -2)	-0.95 (-1.29, -0.61)
United States of America	513 (439.5,567.5)	167.4 (143.2,184.8)	1075.8 (716.1,1171.7)	212.2 (138.1,230.5)	26.7 (-13,34.6)	0.82 (0.66, 0.97)
North Africa and Middle East	1153.3 (702.9,1469.1)	726.3 (448.4,913.1)	2180.4 (1285.2,2768.7)	545 (315.8,682.4)	-25 (-42, -7.4)	-0.99 (-1.13, -0.84)
Afghanistan	107 (31.3,165.7)	1525.4 (462.1,2324.2)	171.9 (55.3,258)	1374.1 (467.2,2020.7)	-9.9 (-34.3,24.1)	-0.36 (-0.43, -0.28)
Algeria	98.7 (48.3,137.8)	929.7 (447,1285.5)	167.6 (75.9,225)	562.3 (246.6,750.9)	-39.5 (-55.2, -14)	-1.71 (-1.81, -1.61)
Bahrain	0.4 (0.3,0.5)	219 (181.5,289.5)	1.1 (0.8,1.3)	121.3 (96.7,160.5)	-44.6 (-59, -25.4)	-2.08 (-2.88, -1.28)
Egypt	260.2 (127.6,396.4)	953 (460.2,1434.9)	479.8 (205.1,839.8)	827.6 (337.7,1419.8)	-13.2 (-44.2,19.6)	-0.49 (-0.83, -0.15)
Iran (Islamic Republic of)	147.3 (121.9,188.2)	645.8 (531.2,785.7)	340.5 (302.5,369)	498.6 (436.9,540.6)	-22.8 (-41.5, -5.7)	-0.88 (-1, -0.76)
Iraq	30.7 (22.5,39.8)	412 (302.6,532.3)	64.6 (50.8,79.4)	314.7 (251.5,376.4)	-23.6 (-45.7,8.1)	-0.95 (-1.18, -0.72)
Jordan	11.7 (9.4,14.3)	1008.4 (800.4,1254.3)	37.4 (25.9,45.4)	664.8 (440.8,798)	-34.1 (-53, -14.3)	-1.46 (-1.83, -1.08)
Kuwait	3.8 (2.9,4.3)	615.6 (483.9,701.9)	9.1 (7.4,12.5)	371.5 (302.9,490.8)	-39.6 (-51.7, -3.2)	-1.79 (-2.59, -0.98)
Lebanon	12.6 (6.3,19)	600.8 (302.3,895.9)	24 (10.7,32)	462.8 (205.5,616)	-23 (-55.3,19.7)	-0.89 (-0.95, -0.83)
Libya	8.1 (4.4,11.3)	440.8 (237,613.7)	23.2 (10.9,33.7)	470.8 (222.8,681.1)	6.8 (-27.7,53.9)	0.34 (-0.27, 0.96)
Morocco	99.5 (50.9,135.7)	775.4 (387.8,1072.6)	195 (89.4,268)	682.9 (305.9,920.6)	-11.9 (-35,23.2)	-0.42 (-0.64, -0.2)
Oman	3.2 (2.3,4.3)	519.7 (375.7,719.3)	5.7 (4.2,7.2)	385.5 (296.4,461.1)	-25.8 (-51.5,10)	-0.81 (-1.23, -0.38)
Palestine	4.4 (3.3,5.7)	561.2 (418.1,706.1)	11.5 (8.6,13.5)	587.4 (410.4,686.5)	4.7 (-29.6,45.4)	0.12 (-0.34, 0.57)
Qatar	0.2 (0.1,0.3)	178 (129.8,248.1)	0.6 (0.5,0.9)	87.1 (64.4,114.3)	-51.1 (-68.7, -24.7)	-2.4 (-3.14, -1.66)
Saudi Arabia	8 (5.7,11.5)	122.9 (89.8,188.6)	21.5 (15.2,27.8)	95.7 (73.3,120.8)	-22.1 (-55.6,14.6)	-0.87 (-1.07, -0.67)

Sudan	92.3 (40.8,127.6)	1017.4 (452.2,1393.8)	149.6 (64.3,229.9)	845.6 (353.8,1262.8)	-16.9 (-39.9,19.2)	-0.64 (-0.72, -0.57)
Syrian Arab Republic	11.1 (7.9,14.8)	221.1 (159.5,297)	16.6 (12.7,22.3)	152.8 (117.9,200.3)	-30.9 (-53.5,6.3)	-1.21 (-1.57, -0.85)
Tunisia	25.6 (13.9,32.6)	556.8 (298.9,711.3)	52.9 (24.6,74.5)	443.5 (205.2,622.2)	-20.4 (-44.2,12.7)	-0.81 (-0.88, -0.75)
Turkey	171.7 (119.4,232.6)	543.6 (368,743)	240.4 (172.9,294.9)	292.7 (204.4,360.6)	-46.2 (-63.8, -21.1)	-2.09 (-2.4, -1.79)
United Arab Emirates	3.9 (1.6,7.5)	865.1 (347.2,1576.1)	37.5 (13.4,79.4)	802.3 (297.6,1587.5)	-7.3 (-46.6,64.8)	-0.26 (-0.94, 0.43)
Yemen	52.1 (22,75.6)	1123.5 (477.7,1597.7)	127.8 (53.5,204.6)	1024.1 (426.8,1623.2)	-8.8 (-38.1,36.2)	-0.31 (-0.42, -0.2)
Oceania	21 (14.5,28.6)	686.4 (481.2,916.5)	46.4 (29.9,63.5)	626.3 (419.1,841.6)	-8.8 (-25.9,13.6)	-0.32 (-0.4, -0.24)
American Samoa	0.1 (0.1,0.1)	416.3 (311.7,509.7)	0.1 (0.1,0.2)	290.6 (239.1,350.1)	-30.2 (-46.1, -8.5)	-1.18 (-1.42, -0.95)
Cook Islands	0.3 (0.2,0.3)	2136.7 (1691.8,2614)	0.3 (0.3,0.4)	1356.2 (1110.7,1654.8)	-36.5 (-51.6, -13.9)	-1.55 (-1.62, -1.47)
Fiji	3.1 (2.3,3.9)	810.2 (620.2,1029.5)	4.7 (3.7,6)	633.9 (504.6,797.8)	-21.8 (-44.5,13.5)	-0.86 (-0.97, -0.75)
Guam	0.8 (0.5,1)	1142 (708.5,1328.2)	0.8 (0.6,1)	417.7 (340.1,516.5)	-63.4 (-72.2, -35.9)	-3.41 (-3.94, -2.88)
Kiribati	0.3 (0.2,0.3)	652.8 (488.1,805.7)	0.4 (0.3,0.5)	482.1 (360.8,603.2)	-26.2 (-45, -2.3)	-1.04 (-1.12, -0.95)
Marshall Islands	0.2 (0.1,0.2)	974.9 (627.5,1317)	0.3 (0.2,0.5)	903.8 (538,1249)	-7.3 (-30.4,24.3)	-0.26 (-0.37, -0.15)
Micronesia (Federated States of)	0.5 (0.3,0.7)	1031.8 (646.3,1349.1)	0.7 (0.4,1)	938.5 (526.8,1304.2)	-9 (-39.4,26.6)	-0.33 (-0.41, -0.25)
Nauru	0 (0,0)	853.4 (543.3,1136.3)	0 (0,0.1)	758.3 (435.5,1033.6)	-11.1 (-31.8,15.1)	-0.41 (-0.57, -0.26)
Niue	0 (0,0)	599.2 (404.4,816.9)	0 (0,0)	467.7 (297.4,637.2)	-21.9 (-41.9,5.6)	-0.84 (-0.95, -0.73)
Northern Mariana Islands	0 (0,0.1)	254 (171.6,326.1)	0.1 (0.1,0.1)	151.6 (120.3,181.7)	-40.3 (-54.2, -15.7)	-1.64 (-2.01, -1.27)
Palau	0 (0,0)	91.5 (65.4,125.2)	0 (0,0)	70.3 (54.5,88.5)	-23.2 (-47.4,12)	-0.9 (-1.01, -0.8)
Papua New Guinea	12 (7.2,17.9)	629 (379.4,920.4)	31.5 (18.4,46.4)	633.3 (381.1,910.9)	0.7 (-23.1,34.1)	0 (-0.09, 0.1)
Samoa	0.6 (0.4,0.8)	711.3 (502.2,906.1)	0.9 (0.6,1.1)	582.3 (382,767.2)	-18.1 (-40,10.3)	-0.69 (-0.74, -0.63)
Solomon Islands	1.3 (0.8,1.9)	876.5 (530.8,1228.9)	2.9 (1.5,4.4)	820.8 (462.9,1209.2)	-6.3 (-33.9,25.3)	-0.21 (-0.39, -0.03)
Tokelau	0 (0,0)	714.6 (469.7,1024.6)	0 (0,0)	496.3 (328.5,678)	-30.5 (-49.2, -1.1)	-1.25 (-1.28, -1.22)
Tonga	0.1 (0.1,0.1)	199.6 (144.8,256.8)	0.1 (0.1,0.2)	173.8 (130.9,224.7)	-12.9 (-36.2,17.1)	-0.44 (-0.62, -0.25)
Tuvalu	0.1 (0,0.1)	908.9 (584.5,1251.2)	0.1 (0,0.1)	660.1 (417,917.2)	-27.4 (-48.1,3.5)	-1.09 (-1.16, -1.03)
Vanuatu	0.5 (0.3,0.6)	674.3 (434.9,916.5)	1.3 (0.8,1.8)	718.7 (430,994.7)	6.6 (-24,51.6)	0.21 (-0.04, 0.45)
South Asia	1684.2 (998.2,2289.4)	334.1 (206.3,452.4)	3002.4 (2193.4,3898.3)	229.2 (166.1,296.9)	-31.4 (-44.7, -5)	-1.27 (-1.72, -0.81)

Bangladesh	174.1 (93.7,241.1)	387.8 (212.7,532.6)	319 (196.1,504.7)	261.4 (162.2,408.9)	-32.6 (-55.9,15.6)	-1.29 (-1.68, -0.91)
Bhutan	0.8 (0.4,1.1)	346.5 (186.9,509)	1.3 (0.9,1.7)	243.8 (177.9,327.3)	-29.6 (-55.7,18.9)	-1.2 (-1.26, -1.14)
India	1279.5 (778.9,1793.8)	321.6 (203.3,451.2)	2255.8 (1523.6,2999.1)	211.9 (143.3,281.8)	-34.1 (-48.3, -9.5)	-1.4 (-2.1, -0.69)
Nepal	24.7 (15.1,38.4)	284.9 (183.1,464.5)	47 (33.3,64.6)	232.6 (166.9,319)	-18.3 (-43.3,23.5)	-0.7 (-0.8, -0.61)
Pakistan	205.1 (121.9,275.9)	375.7 (229.8,515.2)	379.3 (241.6,499.3)	370.7 (246.9,481.9)	-1.3 (-28.3,25.2)	-0.03 (-0.09, 0.02)
Southeast Asia	1233 (859.6,1457.9)	497.9 (358.1,584.1)	2498.9 (1585.1,2834.2)	422.9 (274.2,476.8)	-15.1 (-29.6,4.1)	-0.54 (-0.64, -0.43)
Cambodia	30.3 (17.2,41.4)	682.1 (395,913)	60.1 (36.9,78.1)	524.5 (328.1,667.6)	-23.1 (-47.7,19.3)	-0.91 (-0.96, -0.86)
Indonesia	593.7 (365.6,744.8)	620.5 (396.2,773.4)	1162.9 (649.5,1428.8)	575.8 (334.8,693.7)	-7.2 (-24.1,18.2)	-0.25 (-0.31, -0.19)
Lao People's Democratic Republic	15.5 (8,23.6)	769.7 (410.9,1165.8)	22.3 (11.8,31.4)	531 (292,744.5)	-31 (-53.1,10.9)	-1.28 (-1.38, -1.17)
Malaysia	10.3 (8.1,12.1)	112.1 (88.4,131.8)	15 (11.7,18.8)	57.3 (45.1,72.3)	-48.9 (-62.5, -25.8)	-2.24 (-2.63, -1.84)
Maldives	0.2 (0.1,0.3)	232.1 (107.6,336.8)	0.3 (0.3,0.4)	122.4 (94.5,147.3)	-47.3 (-66.9,19.4)	-2.29 (-2.58, -1.99)
Mauritius	4.7 (3.6,5.1)	659.1 (514.8,721.1)	7.9 (6.2,9.9)	476.4 (374.3,590.4)	-27.7 (-41.1, -8.4)	-0.96 (-1.3, -0.62)
Myanmar	166.7 (95.2,243.2)	720 (427.8,1020.4)	201.2 (117.8,267.2)	451.3 (271,593.9)	-37.3 (-55.9, -1)	-1.61 (-1.72, -1.51)
Philippines	110.7 (98.3,130)	413.9 (368,480.2)	557.1 (375.2,671.4)	726.4 (513.9,866.7)	75.5 (14.9,116.1)	2.02 (1.56, 2.48)
Seychelles	0.8 (0.6,0.9)	1367.7 (1125.6,1509.6)	0.8 (0.7,1)	806.9 (698.8,938.8)	-41 (-49.7, -19.5)	-1.84 (-2.01, -1.67)
Sri Lanka	37.7 (33.5,43.3)	353.7 (314.1,426)	77.2 (54.3,102.4)	311.6 (222.3,410.1)	-11.9 (-42,22)	-0.39 (-0.94, 0.17)
Thailand	23.8 (18.2,29.8)	69.4 (52.3,88.6)	47.6 (36.4,61.5)	47.9 (36.7,61.6)	-31 (-49.4, -3.7)	-1.23 (-1.55, -0.91)
Timor-Leste	1.3 (0.7,1.9)	500.3 (293.1,731.7)	4.2 (2.4,6.2)	536.3 (313.8,791.3)	7.2 (-27.9,64)	0.25 (0.12, 0.38)
Viet Nam	235.7 (150.8,306.6)	607.9 (397.3,786)	339.1 (200.8,497)	386 (235.5,554.1)	-36.5 (-57.8, -5.2)	-1.56 (-1.6, -1.53)
Southern Latin America	112.5 (102.4,127.7)	251.7 (229.9,292.9)	175.5 (153.8,209.6)	207 (182.3,247.8)	-17.8 (-26.8, -7.8)	-0.65 (-0.93, -0.37)
Uruguay	6 (5.2,6.9)	153.6 (135.2,176.2)	8.7 (6.6,9.9)	149.3 (111.9,169.6)	-2.8 (-25.2,9.6)	-0.13 (-0.31, 0.06)
Argentina	86.5 (79.1,100)	275.7 (251.9,324.5)	121.7 (107.7,153.8)	221.5 (196.6,280.6)	-19.7 (-27.2, -7.4)	-0.77 (-1.04, -0.49)
Chile	19.9 (17.3,22.3)	218.2 (187.6,242.9)	45.1 (34.2,51.2)	188.8 (142.8,214.7)	-13.5 (-35, -2.4)	-0.49 (-0.68, -0.29)
Southern Sub-Saharan Africa	169.6 (150.4,196.2)	608.8 (535.7,712)	309.5 (277.9,349.1)	580.3 (519.5,648.9)	-4.7 (-17.5,5.9)	-0.16 (-0.73, 0.4)
Botswana	4.3 (2.9,5.9)	771 (536.5,1057.2)	9.4 (6.2,13.5)	720.7 (474.3,1009.5)	-6.5 (-42.1,42.9)	-0.23 (-0.49, 0.03)
Eswatini	2.5 (1.9,3.2)	881.1 (676.8,1102.7)	5.2 (3.2,8)	940.1 (601.4,1378.5)	6.7 (-29.4,57.2)	0.21 (-0.14, 0.56)

Lesotho	7.6 (5.8,9.8)	798.7 (615.7,1018.6)	14 (9.2,19.4)	1155.5 (776.4,1565.7)	44.7 (-2.3,111)	1.29 (1.08, 1.51)
Namibia	5.9 (4.2,7.8)	853.7 (606.8,1119.7)	9.6 (6.3,13.8)	713.1 (488,1002.4)	-16.5 (-43.2,22.1)	-0.58 (-0.76, -0.4)
South Africa	132.2 (116.6,156)	614.4 (535.9,744.9)	243.4 (216,275.6)	574.4 (504.8,647)	-6.5 (-19,3.5)	-0.26 (-0.93, 0.43)
Zimbabwe	17.1 (13.9,23.7)	440.4 (360.6,610.2)	27.8 (18.6,45)	407.5 (280.2,675.3)	-7.5 (-34.7,30.9)	-0.28 (-0.62, 0.06)
Tropical Latin America	370.6 (285.8,391.5)	415.4 (325.7,440.7)	572.4 (515.6,781.2)	240.4 (215.7,327.9)	-42.1 (-47.6, -8.8)	-1.85 (-2.03, -1.67)
Brazil	365.4 (280.5,386.1)	419.8 (328.4,445.5)	558.1 (502.3,769.8)	239.8 (215.3,330.2)	-42.9 (-48.3, -9)	-1.89 (-2.07, -1.7)
Paraguay	5.2 (4.4,5.8)	243.7 (202.8,273.4)	14.2 (10.8,18.2)	265.6 (200.2,340.5)	9 (-17.9,41.2)	0.38 (-0.26, 1.02)
Western Europe	721.6 (623.7,807.7)	121.9 (105.6,136.7)	1123 (847.3,1253.2)	103.5 (80.7,115.7)	-15.1 (-34, -4.2)	-0.56 (-0.68, -0.44)
Andorra	0.1 (0.1,0.1)	167.6 (121,223.1)	0.2 (0.1,0.2)	126.4 (96.7,162.7)	-24.6 (-47.2,7.1)	-0.96 (-1.07, -0.84)
Austria	19.3 (17.2,23.8)	156.4 (139.7,196.3)	32.8 (21.3,37.5)	155.7 (104.7,177.1)	-0.4 (-41.5,13.1)	-0.02 (-0.22, 0.19)
Belgium	7.8 (6.9,9.8)	50.3 (44.2,63)	10.2 (8.5,13.8)	37.9 (32,52.2)	-24.8 (-34.9, -6.5)	-0.99 (-1.35, -0.64)
Cyprus	1.4 (0.8,1.8)	231.6 (130.6,304.6)	2.1 (1.3,2.4)	129.1 (73.8,152)	-44.3 (-58.8, -4)	-2.01 (-2.39, -1.64)
Denmark	3.8 (3.1,4.4)	45.9 (36,51.9)	3.8 (3.2,4.8)	30.6 (26.3,39.4)	-33.2 (-44.2, -1.5)	-1.35 (-1.64, -1.07)
Finland	7.4 (6.3,8.2)	104.8 (88.1,115.8)	17.7 (9.7,20.4)	138.1 (71.9,158.8)	31.7 (-33.2,52)	0.91 (0.4, 1.43)
France	71 (51.9,79.7)	80.4 (58.9,89.5)	86.7 (65.1,103.1)	48.7 (38.6,58.6)	-39.4 (-46.8, -12.1)	-1.71 (-1.84, -1.59)
Germany	283.3 (251.5,331.9)	218.3 (194.1,256.7)	373.8 (310,438.2)	166.2 (141.2,205.8)	-23.9 (-34.3, -2.3)	-0.93 (-1.23, -0.63)
Greece	16.3 (14.4,19.7)	110.6 (97.6,134.9)	34.1 (25.7,39.1)	117 (90.4,132.4)	5.7 (-22.6,19.2)	0.08 (-0.5, 0.67)
Iceland	0.2 (0.1,0.2)	53.4 (46.8,66.7)	0.3 (0.2,0.3)	44.1 (35.3,52.1)	-17.4 (-37.7, -4.5)	-0.62 (-0.74, -0.5)
Ireland	2.4 (2,2.7)	60.2 (52.2,68.7)	2.7 (2.2,3.7)	35.2 (29.2,48)	-41.4 (-50, -13.5)	-1.87 (-2.18, -1.55)
Israel	5.6 (2.9,6.4)	119.3 (63.7,135.4)	3.4 (2.7,7.8)	27.8 (21.8,63.6)	-76.7 (-82, -9)	-4.89 (-5.53, -4.24)
Italy	167.2 (125.1,182.1)	186.1 (144.5,203)	310.6 (194.5,353.3)	173.1 (111.4,195.5)	-7 (-32.2,1.3)	-0.25 (-0.46, -0.04)
Luxembourg	0.4 (0.4,0.5)	81.6 (72,102.3)	0.7 (0.6,0.9)	63.5 (50.7,80.7)	-22.2 (-41.3, -5.3)	-0.9 (-1, -0.8)
Malta	0.4 (0.4,0.5)	105.8 (92.3,130.3)	0.7 (0.6,0.9)	74.2 (61.3,93.9)	-29.9 (-42.1, -15)	-1.2 (-1.56, -0.84)
Monaco	0.1 (0,0.1)	70.9 (56,93.1)	0.1 (0.1,0.1)	77.5 (61.3,94.2)	9.3 (-19,40.9)	0.32 (0.19, 0.46)
Netherlands	8 (7.2,9.7)	39.6 (35.6,47.8)	13.1 (10.4,15.2)	35.9 (29,41.4)	-9.4 (-30.9,2.2)	-0.32 (-0.58, -0.06)
Norway	3.8 (2.9,4.2)	52.7 (40,57.9)	3 (2.5,4.5)	28 (23.5,42.7)	-46.9 (-53.9, -3)	-2.13 (-2.38, -1.88)

Portugal	13.3 (9.7,14.6)	101.8 (75.3,111.8)	21.4 (14.5,24.8)	72.7 (51.6,83.3)	-28.6 (-42.1, -9.3)	-1.17 (-1.31, -1.04)
San Marino	0 (0,0)	84.1 (68.6,107.3)	0.1 (0.1,0.1)	91.8 (64.9,126.9)	9.2 (-28.3,57.1)	0.32 (0.22, 0.42)
Spain	34.4 (30.2,42.7)	63.9 (55.8,80.4)	88.6 (53.3,104)	72.5 (46.4,84.1)	13.6 (-35.3,30.9)	0.42 (0.22, 0.63)
Sweden	6.3 (5.2,9.4)	39.2 (32.8,58.3)	19.7 (9.8,23.2)	78.6 (40.7,92.1)	100.3 (-22,140.2)	2.43 (2.16, 2.71)
Switzerland	10.4 (6.3,12.5)	91.4 (57.7,109.1)	21.1 (10.2,25.2)	94.9 (50.5,111.5)	3.8 (-28.3,26.2)	0.1 (-0.05, 0.25)
United Kingdom	58.1 (53.4,78.5)	64.9 (60.1,84.8)	75.2 (67.1,100.7)	60.7 (54.3,74)	-6.6 (-29.5,1.1)	-0.22 (-0.3, -0.14)
Western Sub-Saharan Africa	345.3 (250.3,439.7)	387.9 (287,490.3)	761.6 (475.9,972.6)	388.9 (247.3,487.7)	0.2 (-36.7,26.8)	0.03 (-0.07, 0.13)
Benin	7.7 (5.6,9.7)	374.5 (276,466.3)	22.5 (13.3,32.4)	437.3 (261.7,613.2)	16.8 (-24.4,64.5)	0.55 (0.41, 0.69)
Burkina Faso	18.2 (12.7,24.1)	408.9 (291.2,539.2)	55.8 (30.8,75.4)	599.3 (337.2,795.1)	46.6 (-11.5,97.8)	1.36 (1.17, 1.55)
Cabo Verde	0.7 (0.5,0.9)	311.8 (236.9,380)	1.2 (0.9,1.4)	273.1 (213.3,329.6)	-12.4 (-35.2,20.4)	-0.43 (-0.7, -0.16)
Cameroon	18.6 (11.5,25.1)	401.9 (255.1,541.2)	52.2 (28.8,81.8)	409.4 (229.7,625.8)	1.9 (-35.1,54.1)	0.08 (-0.02, 0.18)
Chad	11.2 (7.6,15.1)	392.8 (271.6,528)	29.7 (16,41.9)	497.6 (273.2,685.4)	26.7 (-16.4,74.8)	0.83 (0.66, 0.99)
CÃ´te d'Ivoire	14.8 (9.8,19.9)	346.5 (239.8,453.6)	47.5 (25.2,70.6)	419.3 (233.2,607)	21 (-22,68.2)	0.66 (0.48, 0.84)
Gambia	1.1 (0.8,1.5)	314.4 (226.4,416.1)	4.8 (2.7,7.1)	485.6 (268.4,698.9)	54.4 (-4.9,132.5)	1.6 (0.76, 2.44)
Ghana	22.9 (15.6,30.1)	349.2 (241.9,453.7)	64.3 (37.6,86.6)	388.6 (230.9,510.8)	11.3 (-28.3,51.8)	0.38 (0.23, 0.54)
Guinea	13.7 (8.2,18.8)	407.7 (246.7,551.1)	30.5 (15.5,43)	526.9 (274.8,729.1)	29.3 (-20.9,89.5)	0.9 (0.69, 1.12)
Guinea-Bissau	2.5 (1.2,3.4)	563 (284.9,760.5)	5.3 (2.4,7.8)	656.3 (308.1,953.5)	16.6 (-25.9,70.2)	0.54 (0.43, 0.65)
Liberia	4.1 (2.6,5.4)	366.9 (237.3,486.1)	9.5 (5,15)	431.5 (229.8,661.6)	17.6 (-26.4,70.3)	0.53 (0.35, 0.7)
Mali	18.8 (10.9,25.5)	441.3 (262.6,598)	40.8 (23.5,55)	450.4 (262.6,600.5)	2.1 (-31.8,38.1)	0.11 (-0.13, 0.36)
Mauritania	4.4 (3,5.8)	432.4 (294.9,571.4)	7 (4.3,9.8)	335 (206.9,454)	-22.5 (-51.2,8.8)	-0.88 (-1.08, -0.69)
Niger	10.5 (6.5,15.1)	358.4 (225.9,514.7)	30.4 (17,46)	374.9 (214.2,559.1)	4.6 (-29.8,43.8)	0.2 (-0.01, 0.42)
Nigeria	175 (120.7,244.4)	397.8 (277.5,549.3)	299 (189.2,410.7)	334.1 (214.8,445.2)	-16 (-50.7,19.9)	-0.58 (-0.75, -0.41)
Sao Tome and Principe	0.1 (0.1,0.2)	200.1 (155.5,248.3)	0.2 (0.1,0.3)	183.6 (129.7,237.4)	-8.3 (-33,28.9)	-0.24 (-0.4, -0.07)
Senegal	10.5 (7.6,13.2)	320.5 (232.4,399.2)	29.1 (16.9,40)	380.4 (222.3,521.7)	18.7 (-23.4,63.5)	0.6 (0.19, 1.01)
Sierra Leone	5.6 (3.8,7.7)	289.4 (199.2,393.4)	14.7 (8.4,21.4)	384.4 (221.7,546)	32.8 (-16.1,85.1)	0.98 (0.81, 1.16)
Togo	4.8 (3.6,6.1)	365.6 (278,455.9)	16.8 (9.6,23.6)	440.1 (256.2,600.2)	20.4 (-22.3,68.5)	0.64 (0.39, 0.9)

Table S5. Changes in DALYs number according to population-level determinants from 1990 to 2019.

	Overall difference [a]	Change due to population-determinants (contribution to the total change)		
		Aging [b]	Population [c]	Epidemiological change [d]
**Global**	7564389.94	6903887.79 (91.27%)	6549676.76 (86.59%)	-5889174.61 (-77.85%)
**Sex**				
Male	3831838.28	3388280.43 (88.42%)	3057120.57 (79.78%)	-2613562.73 (-68.21%)
Female	3732551.66	3502667.83 (93.84%)	3496702.16 (93.68%)	-3266818.33 (-87.52%)
**SDI groups**				
High SDI	900722.73	729855.42 (81.03%)	380602.39 (42.26%)	-209735.07 (-23.29%)
High-middle SDI	1211402.3	1697408.69 (140.12%)	749423.92 (61.86%)	-1235430.31 (-101.98%)
Middle SDI	2604890.9	4349777.42 (166.99%)	2439346.42 (93.64%)	-4184232.93 (-160.63%)
Low-middle SDI	1742784.89	1399025.49 (80.28%)	1495165.55 (85.79%)	-1151406.15 (-66.07%)
Low SDI	1096621.5	101486.57 (9.25%)	1572752.22 (143.42%)	-577617.3 (-52.67%)
**Geographic regions**				
Andean Latin America	43731.27	34422.13 (78.71%)	34812.46 (79.61%)	-25503.32 (-58.32%)
Bolivia (Plurinational State of)	12250.64	7011.98 (57.24%)	10825.74 (88.37%)	-5587.08 (-45.61%)
Ecuador	25288.45	14939.64 (59.08%)	17466.47 (69.07%)	-7117.66 (-28.15%)
Peru	6192.18	10781.97 (174.12%)	8845.93 (142.86%)	-13435.72 (-216.98%)
Australasia	6709.08	8328.42 (124.14%)	6217.84 (92.68%)	-7837.19 (-116.81%)
Australia	6285.78	6826.25 (108.6%)	5257.96 (83.65%)	-5798.43 (-92.25%)
New Zealand	423.3	1531.88 (361.89%)	903.93 (213.54%)	-2012.51 (-475.44%)
Caribbean	91771.09	56788.99 (61.88%)	41388.61 (45.1%)	-6406.51 (-6.98%)
Antigua and Barbuda	233.72	91.63 (39.2%)	150.13 (64.23%)	-8.04 (-3.44%)

Bahamas	2553.36	1470.48 (57.59%)	1082.99 (42.41%)	-0.11 (0%)
Barbados	426.37	356.33 (83.57%)	165.51 (38.82%)	-95.46 (-22.39%)
Belize	756.69	189.57 (25.05%)	495.55 (65.49%)	71.56 (9.46%)
Bermuda	53.3	97.27 (182.51%)	10.58 (19.84%)	-54.55 (-102.35%)
Cuba	27810.9	13303.96 (47.84%)	1157.47 (4.16%)	13349.47 (48%)
Dominica	42.29	215.22 (508.92%)	-53.62 (-126.8%)	-119.31 (-282.12%)
Dominican Republic	22988.45	11885.08 (51.7%)	9652.4 (41.99%)	1450.97 (6.31%)
Grenada	141.16	98.47 (69.76%)	85.66 (60.68%)	-42.97 (-30.44%)
Guyana	1198.01	3295.61 (275.09%)	5.69 (0.47%)	-2103.3 (-175.57%)
Haiti	22239.66	4963.75 (22.32%)	27636.6 (124.27%)	-10360.7 (-46.59%)
Jamaica	723.2	4846.66 (670.17%)	2381.36 (329.28%)	-6504.82 (-899.45%)
Puerto Rico	7082.24	7597.43 (107.27%)	-307.42 (-4.34%)	-207.77 (-2.93%)
Saint Kitts and Nevis	82.36	48.27 (58.6%)	91.9 (111.58%)	-57.8 (-70.18%)
Saint Lucia	378.7	482.4 (127.38%)	171.26 (45.22%)	-274.96 (-72.61%)
Saint Vincent and the Grenadines	382.39	403.22 (105.45%)	19.01 (4.97%)	-39.84 (-10.42%)
Suriname	1210.75	835.58 (69.01%)	773.7 (63.9%)	-398.52 (-32.92%)
Trinidad and Tobago	-157.28	4255.68 (-2705.72%)	931.01 (-591.93%)	-5343.97 (3397.64%)
United States Virgin Islands	459.48	478.38 (104.11%)	-12.39 (-2.7%)	-6.51 (-1.42%)
Central Asia	145095.86	26036.76 (17.94%)	63978.62 (44.09%)	55080.48 (37.96%)
Armenia	4213.05	5110.95 (121.31%)	-905.17 (-21.48%)	7.27 (0.17%)
Azerbaijan	13747.48	7620.75 (55.43%)	10973.41 (79.82%)	-4846.69 (-35.26%)
Georgia	39254.51	17127.37 (43.63%)	-14921.23 (-38.01%)	37048.37 (94.38%)
Kazakhstan	10290.8	8492.23 (82.52%)	4835.62 (46.99%)	-3037.05 (-29.51%)
Kyrgyzstan	4256.13	233.28 (5.48%)	3387.79 (79.6%)	635.06 (14.92%)
Mongolia	-231.81	1987.95 (-857.59%)	2429.8 (-1048.19%)	-4649.55 (2005.78%)
Tajikistan	16431.08	-2609.82 (-15.88%)	15445.48 (94%)	3595.43 (21.88%)

Turkmenistan	6774.11	3202.4 (47.27%)	2582.21 (38.12%)	989.5 (14.61%)
Uzbekistan	50360.51	2614.45 (5.19%)	24152.28 (47.96%)	23593.78 (46.85%)
Central Europe	264376.59	302181.53 (114.3%)	-44281.75 (-16.75%)	6476.81 (2.45%)
Albania	1063.49	4300.35 (404.36%)	-821.23 (-77.22%)	-2415.63 (-227.14%)
Bosnia and Herzegovina	406.84	6790.74 (1669.13%)	-2744.63 (-674.62%)	-3639.26 (-894.51%)
Bulgaria	110598.65	51380.06 (46.46%)	-27671.76 (-25.02%)	86890.35 (78.56%)
Croatia	3274.85	13353.04 (407.74%)	-3150.74 (-96.21%)	-6927.44 (-211.53%)
Czechia	15094.09	6601.17 (43.73%)	553.98 (3.67%)	7938.94 (52.6%)
Hungary	20377.88	29428.07 (144.41%)	-5416.27 (-26.58%)	-3633.93 (-17.83%)
Montenegro	368.71	283.7 (76.94%)	-5.07 (-1.38%)	90.08 (24.43%)
North Macedonia	5296.7	6587.07 (124.36%)	831.38 (15.7%)	-2121.75 (-40.06%)
Poland	43659.19	55739.88 (127.67%)	814.25 (1.87%)	-12894.94 (-29.54%)
Romania	38045.35	100622.16 (264.48%)	-33144.68 (-87.12%)	-29432.13 (-77.36%)
Serbia	11602.32	21164.11 (182.41%)	-2817.24 (-24.28%)	-6744.55 (-58.13%)
Slovakia	10694.77	5238.78 (48.98%)	375.06 (3.51%)	5080.92 (47.51%)
Slovenia	3893.75	5376.72 (138.09%)	424.09 (10.89%)	-1907.06 (-48.98%)
Central Latin America	167611.72	209769.33 (125.15%)	133636.08 (79.73%)	-175793.69 (-104.88%)
Colombia	8076.15	75169.52 (930.76%)	35222.51 (436.13%)	-102315.87 (-1266.89%)
Costa Rica	5233.36	3704.83 (70.79%)	2458.12 (46.97%)	-929.59 (-17.76%)
El Salvador	2109.68	3001.69 (142.28%)	977.41 (46.33%)	-1869.42 (-88.61%)
Guatemala	5667.17	3799.08 (67.04%)	7772.49 (137.15%)	-5904.4 (-104.19%)
Honduras	10519	3755.11 (35.7%)	7818.76 (74.33%)	-1054.86 (-10.03%)
Mexico	77996.65	74408.28 (95.4%)	42244.37 (54.16%)	-38655.99 (-49.56%)
Nicaragua	7212.21	3767.51 (52.24%)	3562.05 (49.39%)	-117.35 (-1.63%)
Panama	3747.58	1459.74 (38.95%)	1644.94 (43.89%)	642.9 (17.16%)
Venezuela (Bolivarian Republic of)	47049.92	53710.6 (114.16%)	30385.27 (64.58%)	-37045.95 (-78.74%)

Central Sub-Saharan Africa	229605.05	2408.9 (1.05%)	309712.07 (134.89%)	-82515.93 (-35.94%)
Angola	38746.77	221.38 (0.57%)	68619.97 (177.1%)	-30094.57 (-77.67%)
Central African Republic	10300.47	-653.14 (-6.34%)	14341.69 (139.23%)	-3388.08 (-32.89%)
Congo	9027.08	3240.3 (35.9%)	15287.07 (169.35%)	-9500.29 (-105.24%)
Democratic Republic of the Congo	169074.05	2354.75 (1.39%)	199248.68 (117.85%)	-32529.39 (-19.24%)
Equatorial Guinea	295.65	-857.9 (-290.17%)	3921.83 (1326.5%)	-2768.28 (-936.33%)
Gabon	2161.02	520.49 (24.09%)	4237.8 (196.1%)	-2597.26 (-120.19%)
East Asia	636447.87	4660652 (732.29%)	1112312.87 (174.77%)	-5136517 (-807.06%)
China	552735.36	4556286.7 (824.32%)	1085347.04 (196.36%)	-5088898.38 (-920.68%)
Democratic People's Republic of Korea	61318.42	45599.46 (74.37%)	17925.82 (29.23%)	-2206.86 (-3.6%)
Taiwan (Province of China)	22394.09	59445.62 (265.45%)	9703.79 (43.33%)	-46755.31 (-208.78%)
Eastern Europe	221695.88	96530.57 (43.54%)	-25133.4 (-11.34%)	150298.71 (67.79%)
Belarus	-6107.75	3145.31 (-51.5%)	-1082.98 (17.73%)	-8170.08 (133.77%)
Estonia	23346.54	6588.45 (28.22%)	-2579.93 (-11.05%)	19338.02 (82.83%)
Latvia	8366.9	2538.97 (30.35%)	-1989.26 (-23.78%)	7817.19 (93.43%)
Lithuania	5450.84	2722.13 (49.94%)	-1739.95 (-31.92%)	4468.67 (81.98%)
Republic of Moldova	21040.32	7020.06 (33.36%)	-2663.83 (-12.66%)	16684.1 (79.3%)
Russian Federation	164848.99	76586.36 (46.46%)	-7492.88 (-4.55%)	95755.51 (58.09%)
Ukraine	4750.04	5047.46 (106.26%)	-3565.95 (-75.07%)	3268.54 (68.81%)
Eastern Sub-Saharan Africa	325421.26	30756.69 (9.45%)	730023.34 (224.33%)	-435358.76 (-133.78%)
Burundi	4331.37	-2407.43 (-55.58%)	20936.21 (483.36%)	-14197.41 (-327.78%)
Comoros	976.71	991.26 (101.49%)	1140.93 (116.81%)	-1155.48 (-118.3%)
Djibouti	2204.06	1319.27 (59.86%)	2127.64 (96.53%)	-1242.86 (-56.39%)
Eritrea	11389.99	4163.91 (36.56%)	15314.98 (134.46%)	-8088.9 (-71.02%)
Ethiopia	-23865.78	11973.92 (-50.17%)	189458.93 (-793.85%)	-225298.63 (944.02%)

Kenya	66725.86	15709.8 (23.54%)	58260.44 (87.31%)	-7244.38 (-10.86%)
Madagascar	62981.11	-1278.71 (-2.03%)	86687.24 (137.64%)	-22427.42 (-35.61%)
Malawi	13608.63	250.97 (1.84%)	25001.66 (183.72%)	-11644 (-85.56%)
Mozambique	37338.8	-14921.53 (-39.96%)	59101.84 (158.29%)	-6841.51 (-18.32%)
Rwanda	-3504.86	7087.73 (-202.23%)	21538.03 (-614.52%)	-32130.61 (916.75%)
Somalia	27806.01	-4428.68 (-15.93%)	53942.36 (194%)	-21707.67 (-78.07%)
South Sudan	-258.46	402.66 (-155.79%)	9963.05 (-3854.79%)	-10624.17 (4110.58%)
Uganda	34882.91	-4640.62 (-13.3%)	51615.73 (147.97%)	-12092.2 (-34.67%)
United Republic of Tanzania	53805.54	6673.83 (12.4%)	98066.48 (182.26%)	-50934.76 (-94.66%)
Zambia	36689.88	1891.09 (5.15%)	36763.04 (100.2%)	-1964.25 (-5.35%)
High-income Asia Pacific	-3137.96	263900.11 (-8409.92%)	20982.83 (-668.68%)	-288020.9 (9178.6%)
Brunei Darussalam	246.74	275.67 (111.72%)	274.28 (111.16%)	-303.21 (-122.88%)
Japan	-2144.48	201260.24 (-9385.02%)	2858.66 (-133.3%)	-206263.38 (9618.33%)
Republic of Korea	-10728.22	86088.46 (-802.45%)	15669.72 (-146.06%)	-112486.41 (1048.51%)
Singapore	9488	5965.8 (62.88%)	5638.97 (59.43%)	-2116.76 (-22.31%)
High-income North America	575985.39	221449.62 (38.45%)	203193.16 (35.28%)	151342.62 (26.28%)
Canada	13149.81	8678.25 (66%)	5522.76 (42%)	-1051.21 (-7.99%)
Greenland	28.71	53.25 (185.48%)	1.04 (3.62%)	-25.58 (-89.1%)
United States of America	562801.39	206315 (36.66%)	195613.61 (34.76%)	160872.79 (28.58%)
North Africa and Middle East	1027121.49	629795.37 (61.32%)	930199.17 (90.56%)	-532873.06 (-51.88%)
Afghanistan	64925.07	-95386.47 (-146.92%)	184652.75 (284.41%)	-24341.2 (-37.49%)
Algeria	68904.78	79225.85 (114.98%)	68972.22 (100.1%)	-79293.29 (-115.08%)
Bahrain	654.86	499.76 (76.32%)	772.72 (118%)	-617.62 (-94.31%)
Egypt	219541.43	72213.98 (32.89%)	207381.86 (94.46%)	-60054.4 (-27.35%)
Iran (Islamic Republic of)	193201.09	175956.49 (91.07%)	87085.25 (45.07%)	-69840.65 (-36.15%)
Iraq	33981.96	9013.6 (26.52%)	40707.59 (119.79%)	-15739.23 (-46.32%)

Jordan	25736.82	11999.11 (46.62%)	25772.43 (100.14%)	-12034.72 (-46.76%)
Kuwait	5277.94	3547.39 (67.21%)	5931.01 (112.37%)	-4200.46 (-79.59%)
Lebanon	11353.34	8205.17 (72.27%)	8218.46 (72.39%)	-5070.3 (-44.66%)
Libya	15048.93	7602.11 (50.52%)	6645.01 (44.16%)	801.81 (5.33%)
Morocco	95417.13	69154.15 (72.48%)	50444.13 (52.87%)	-24181.15 (-25.34%)
Oman	2500.09	662.53 (26.5%)	3829.18 (153.16%)	-1991.62 (-79.66%)
Palestine	7074.79	502.51 (7.1%)	6467.25 (91.41%)	105.04 (1.48%)
Qatar	446.57	141.79 (31.75%)	837.92 (187.64%)	-533.14 (-119.39%)
Saudi Arabia	13546.97	5838.33 (43.1%)	10942.5 (80.77%)	-3233.86 (-23.87%)
Sudan	57290.14	1071.88 (1.87%)	84989.78 (148.35%)	-28771.52 (-50.22%)
Syrian Arab Republic	5479.06	9929.68 (181.23%)	1683.89 (30.73%)	-6134.52 (-111.96%)
Tunisia	27290.72	24656.7 (90.35%)	12199.2 (44.7%)	-9565.19 (-35.05%)
Turkey	68683.54	146965 (213.97%)	67417.82 (98.16%)	-145699.27 (-212.13%)
United Arab Emirates	33613.62	10398.33 (30.93%)	22460.47 (66.82%)	754.81 (2.25%)
Yemen	75713.06	16855.79 (22.26%)	70000.56 (92.46%)	-11143.29 (-14.72%)
Oceania	25405.02	5279.56 (20.78%)	23011.25 (90.58%)	-2885.78 (-11.36%)
American Samoa	43.53	69.8 (160.33%)	16.49 (37.87%)	-42.75 (-98.2%)
Cook Islands	52.79	217.2 (411.44%)	-17.72 (-33.56%)	-146.69 (-277.88%)
Fiji	1640.84	1976.75 (120.47%)	712.83 (43.44%)	-1048.74 (-63.91%)
Guam	-36.55	644.22 (-1762.39%)	208.03 (-569.11%)	-888.8 (2431.5%)
Kiribati	96.66	53.96 (55.83%)	149.76 (154.93%)	-107.06 (-110.76%)
Marshall Islands	170.22	128.2 (75.32%)	52.68 (30.95%)	-10.67 (-6.27%)
Micronesia (Federated States of)	190.18	265.38 (139.54%)	-12.44 (-6.54%)	-62.76 (-33%)
Nauru	0.83	3.66 (441.44%)	1.05 (126.42%)	-3.88 (-467.86%)
Niue	-3.06	3.65 (-119.17%)	-3.85 (125.77%)	-2.86 (93.4%)
Northern Mariana Islands	29.73	70.45 (236.96%)	-4.63 (-15.57%)	-36.09 (-121.39%)

Palau	5.88	7.37 (125.22%)	1.89 (32.15%)	-3.37 (-57.37%)
Papua New Guinea	19560.96	1799.48 (9.2%)	17707.5 (90.52%)	53.98 (0.28%)
Samoa	241.72	198.38 (82.07%)	188.83 (78.12%)	-145.49 (-60.19%)
Solomon Islands	1523.65	389.93 (25.59%)	1301.78 (85.44%)	-168.05 (-11.03%)
Tokelau	-2.85	1.51 (-52.92%)	-1.42 (49.91%)	-2.94 (103.01%)
Tonga	27.62	38.18 (138.21%)	6.95 (25.17%)	-17.51 (-63.38%)
Tuvalu	4.81	11.47 (238.73%)	15.26 (317.56%)	-21.93 (-456.29%)
Vanuatu	828.59	230.5 (27.82%)	528.56 (63.79%)	69.53 (8.39%)
South Asia	1318148.47	1091876.87 (82.83%)	1165066.53 (88.39%)	-938794.93 (-71.22%)
Bangladesh	144872.22	163226.05 (112.67%)	94495.25 (65.23%)	-112849.08 (-77.9%)
Bhutan	506.62	713.31 (140.8%)	215.78 (42.59%)	-422.46 (-83.39%)
India	976257.26	904703.57 (92.67%)	864310.93 (88.53%)	-792757.25 (-81.2%)
Nepal	22293.06	16470.34 (73.88%)	15529.9 (69.66%)	-9707.19 (-43.54%)
Pakistan	174219.31	-16599.37 (-9.53%)	195258.84 (112.08%)	-4440.15 (-2.55%)
Southeast Asia	1265962.66	922273.84 (72.85%)	666665.77 (52.66%)	-322976.95 (-25.51%)
Cambodia	29778.02	23138.15 (77.7%)	20887.82 (70.15%)	-14247.96 (-47.85%)
Indonesia	569173.21	396600.08 (69.68%)	287164.03 (50.45%)	-114590.9 (-20.13%)
Lao People's Democratic Republic	6716.24	4293.52 (63.93%)	10449.97 (155.59%)	-8027.26 (-119.52%)
Malaysia	4710.04	6451.4 (136.97%)	7652.62 (162.47%)	-9393.97 (-199.45%)
Maldives	144.78	144.75 (99.98%)	226.91 (156.72%)	-226.88 (-156.7%)
Mauritius	3267.68	4621.97 (141.44%)	961.4 (29.42%)	-2315.69 (-70.87%)
Myanmar	34484.55	79634.84 (230.93%)	54321.09 (157.52%)	-99471.38 (-288.45%)
Philippines	446435.04	110117.74 (24.67%)	158306.7 (35.46%)	178010.59 (39.87%)
Seychelles	73.24	250.2 (341.61%)	282.86 (386.2%)	-459.82 (-627.81%)
Sri Lanka	39542.17	34742.06 (87.86%)	13365.01 (33.8%)	-8564.89 (-21.66%)
Thailand	23783.2	30428.76 (127.94%)	7674.2 (32.27%)	-14319.76 (-60.21%)

Timor-Leste	2848.71	1387.36 (48.7%)	1309.96 (45.98%)	151.39 (5.31%)
Viet Nam	103370.81	143769.38 (139.08%)	103305.21 (99.94%)	-143703.78 (-139.02%)
Southern Latin America	63063.81	50577.95 (80.2%)	42314.73 (67.1%)	-29828.86 (-47.3%)
Uruguay	2756.14	2197.91 (79.75%)	654.63 (23.75%)	-96.4 (-3.5%)
Argentina	35166.42	27237.8 (77.45%)	31880.61 (90.66%)	-23951.99 (-68.11%)
Chile	25136.98	20451.15 (81.36%)	9904.09 (39.4%)	-5218.26 (-20.76%)
Southern Sub-Saharan Africa	139852.02	72844.02 (52.09%)	94030.84 (67.24%)	-27022.84 (-19.32%)
Botswana	5189.9	2073.95 (39.96%)	3815.38 (73.52%)	-699.44 (-13.48%)
Eswatini	2690.09	1193.08 (44.35%)	1294.65 (48.13%)	202.36 (7.52%)
Lesotho	6396.01	1059.43 (16.56%)	1535.5 (24.01%)	3801.08 (59.43%)
Namibia	3620.75	1252.28 (34.59%)	4085.84 (112.85%)	-1717.37 (-47.43%)
South Africa	111252.57	62414.93 (56.1%)	75237.98 (67.63%)	-26400.34 (-23.73%)
Zimbabwe	10702.69	3660.55 (34.2%)	8205.01 (76.66%)	-1162.87 (-10.87%)
Tropical Latin America	201783.89	301584.01 (149.46%)	186094.38 (92.22%)	-285894.5 (-141.68%)
Brazil	192769.68	299068.32 (155.14%)	180534.67 (93.65%)	-286833.31 (-148.8%)
Paraguay	9014.21	3520.69 (39.06%)	4793.88 (53.18%)	699.64 (7.76%)
Western Europe	401472.86	399108.61 (99.41%)	114209.33 (28.45%)	-111845.08 (-27.86%)
Andorra	120.62	103.56 (85.86%)	55.05 (45.64%)	-37.99 (-31.5%)
Austria	13535.62	9396.76 (69.42%)	3477.7 (25.69%)	661.15 (4.88%)
Belgium	2420.39	3425.63 (141.53%)	1207.56 (49.89%)	-2212.8 (-91.42%)
Cyprus	694.12	1139.06 (164.1%)	948.61 (136.66%)	-1393.56 (-200.77%)
Denmark	-70.99	932.88 (-1314.13%)	462.92 (-652.1%)	-1466.79 (2066.23%)
Finland	10311.77	6015.24 (58.33%)	1193.46 (11.57%)	3103.06 (30.09%)
France	15680.89	42363.29 (270.16%)	11006.58 (70.19%)	-37688.99 (-240.35%)
Germany	90485.58	143001.78 (158.04%)	19719.62 (21.79%)	-72235.82 (-79.83%)
Greece	17762.51	16686.04 (93.94%)	-126.32 (-0.71%)	1202.8 (6.77%)

Iceland	115.9	86.11 (74.3%)	64.8 (55.91%)	-35.01 (-30.21%)
Ireland	292.48	920.61 (314.76%)	809.72 (276.85%)	-1437.85 (-491.61%)
Israel	-2209.88	1728 (-78.19%)	3468.13 (-156.94%)	-7406.01 (335.13%)
Italy	143350.39	140513.47 (98.02%)	14022.03 (9.78%)	-11185.1 (-7.8%)
Luxembourg	269.69	137.88 (51.13%)	271.58 (100.7%)	-139.77 (-51.83%)
Malta	294.8	416.5 (141.28%)	99.82 (33.86%)	-221.52 (-75.14%)
Monaco	30.41	8.18 (26.9%)	14.58 (47.94%)	7.65 (25.16%)
Netherlands	5153.83	4567.94 (88.63%)	1451.13 (28.16%)	-865.25 (-16.79%)
Norway	-834.82	440.67 (-52.79%)	811.67 (-97.23%)	-2087.15 (250.01%)
Portugal	8164.18	13181.66 (161.46%)	876.82 (10.74%)	-5894.31 (-72.2%)
San Marino	43.56	23.85 (54.74%)	15.57 (35.74%)	4.15 (9.52%)
Spain	54240.21	35358.45 (65.19%)	9840.78 (18.14%)	9040.98 (16.67%)
Sweden	13421.81	2841.01 (21.17%)	2107.85 (15.7%)	8472.95 (63.13%)
Switzerland	10706.14	6000.4 (56.05%)	3683.51 (34.41%)	1022.23 (9.55%)
United Kingdom	17113.74	12225.2 (71.43%)	10359.82 (60.54%)	-5471.28 (-31.97%)
Western Sub-Saharan Africa	416266.6	-38327.31 (-9.21%)	456432.63 (109.65%)	-1838.72 (-0.44%)
Benin	14788.34	-563.68 (-3.81%)	13162.26 (89%)	2189.75 (14.81%)
Burkina Faso	37655.29	-3674.45 (-9.76%)	28865.15 (76.66%)	12464.59 (33.1%)
Cabo Verde	473.48	173.91 (36.73%)	441.74 (93.3%)	-142.16 (-30.02%)
Cameroon	33648.46	-359.12 (-1.07%)	33457.71 (99.43%)	549.87 (1.63%)
Chad	18539.34	-5670.92 (-30.59%)	19252.93 (103.85%)	4957.33 (26.74%)
Coted'Ivoire	32658.89	6218.46 (19.04%)	20937.49 (64.11%)	5502.94 (16.85%)
Gambia	3687.13	508.46 (13.79%)	2029.17 (55.03%)	1149.5 (31.18%)
Ghana	41465.09	8848.68 (21.34%)	29345.3 (70.77%)	3271.11 (7.89%)
Guinea	16782.34	-3833.89 (-22.84%)	15060.52 (89.74%)	5555.71 (33.1%)
Guinea-Bissau	2821.31	-18.08 (-0.64%)	2328.44 (82.53%)	510.95 (18.11%)

Liberia	5467.17	-1367.68 (-25.02%)	5769.46 (105.53%)	1065.39 (19.49%)
Mali	21996.45	-4688.33 (-21.31%)	26707.55 (121.42%)	-22.77 (-0.1%)
Mauritania	2632.3	503.74 (19.14%)	3797.99 (144.28%)	-1669.43 (-63.42%)
Niger	19864.57	-800.98 (-4.03%)	19963.51 (100.5%)	702.05 (3.53%)
Nigeria	124010.16	-37893.19 (-30.56%)	206656.49 (166.64%)	-44753.13 (-36.09%)
Sao Tome and Principe	74.28	3.22 (4.34%)	84.82 (114.19%)	-13.76 (-18.52%)
Senegal	18580.14	3134.48 (16.87%)	12365.59 (66.55%)	3080.06 (16.58%)
Sierra Leone	9150.62	-1522.5 (-16.64%)	7715.7 (84.32%)	2957.43 (32.32%)
Togo	11972.17	2973.07 (24.83%)	7278.45 (60.79%)	1720.65 (14.37%)

a. Change in DALYs number between year 2019 and 1990.

b. Change in DALYs number due to change in the age structure.

c. Change in DALYs number due to change in population number.

d. Change in DALYs number due to epidemiologic changes. Epidemiologic changes refer to the DALYs number change when age structure and population number hold constant.

Table S6. Frontier DALYs, and effective difference by country or territory.

Location	SDI	Age-standardized DALYs (95% UI)	Frontier DALYs	Effective difference	Effective difference rank (Age-standardized DALYs rank)
Afghanistan	0.343	1374.12 (467.17 to 2020.7)	173.34	1200.78	202 (204)
Albania	0.681	99.32 (74.58 to 130.79)	27.88	71.44	36 (32)
Algeria	0.652	562.26 (246.65 to 750.88)	46.77	515.49	154 (147)
American Samoa	0.712	290.56 (239.08 to 350.1)	26.87	263.69	95 (87)
Andorra	0.894	126.37 (96.67 to 162.7)	26.96	99.42	44 (41)
Angola	0.47	799.65 (487.96 to 1071.54)	94.64	705.01	178 (176)
Antigua and Barbuda	0.743	536.95 (425.2 to 637.88)	27.83	509.12	151 (144)
Argentina	0.708	221.46 (196.57 to 280.58)	26.98	194.48	77 (69)
Armenia	0.689	233.45 (123.44 to 280.02)	28.53	204.91	80 (72)
Australia	0.839	38.38 (32 to 45.2)	26.94	11.45	9 (9)
Austria	0.849	155.67	26.84	128.83	55 (51)

		(104.7 to 177.11)			
Azerbaijan	0.683	510.48 (373.69 to 682.34)	26.88	483.59	150 (139)
Bahamas	0.796	1089.42 (866.61 to 1363.28)	26.96	1062.46	198 (197)
Bahrain	0.751	121.27 (96.7 to 160.47)	26.84	94.43	41 (37)
Bangladesh	0.483	261.39 (162.23 to 408.85)	95	166.38	68 (79)
Barbados	0.742	259.95 (210.07 to 322.99)	26.93	233.02	88 (78)
Belarus	0.745	51.38 (37.18 to 86.35)	27.39	23.99	17 (15)
Belgium	0.851	37.86 (31.97 to 52.24)	26.94	10.92	8 (8)
Belize	0.603	393.42 (321.86 to 463.71)	53.82	339.59	115 (108)
Benin	0.352	437.29 (261.72 to 613.19)	163.3	273.99	98 (119)
Bermuda	0.813	125.51 (96.87 to 156.06)	26.98	98.53	42 (39)
Bhutan	0.455	243.84 (177.86 to 327.3)	94.89	148.95	62 (74)
Bolivia (Plurinational State of)	0.566	293.27 (206.44 to 400.08)	70.55	222.72	87 (89)
Bosnia and Herzegovina	0.718	144.59	27.89	116.7	49 (45)

		(114.08 to 213.69)			
Botswana	0.634	720.66 (474.33 to 1009.54)	47.69	672.97	174 (172)
Brazil	0.64	239.83 (215.27 to 330.22)	48.51	191.31	75 (73)
Brunei Darussalam	0.823	275.28 (233.61 to 369.95)	26.96	248.32	92 (82)
Bulgaria	0.764	1238.83 (758.46 to 1564.62)	27.35	1211.48	203 (200)
Burkina Faso	0.257	599.32 (337.23 to 795.1)	240	359.32	120 (156)
Burundi	0.284	651.72 (282.79 to 1155.59)	229.55	422.18	131 (160)
Cabo Verde	0.525	273.15 (213.25 to 329.62)	90.22	182.92	72 (81)
Cambodia	0.469	524.5 (328.09 to 667.55)	94.67	429.83	133 (140)
Cameroon	0.49	409.36 (229.74 to 625.78)	94.71	314.65	110 (111)
Canada	0.873	39.09 (29.3 to 44.38)	26.95	12.14	10 (10)
Central African Republic	0.274	1321.78 (787.64 to 1924.36)	237.45	1084.33	199 (202)
Chad	0.238	497.61 (273.19 to 685.44)	282.72	214.88	83 (137)
Chile	0.759	188.79	27.37	161.42	66 (61)

		(142.78 to 214.7)			
China	0.686	312.88 (214.08 to 363.65)	26.91	285.97	101 (91)
Colombia	0.633	152.77 (115.33 to 244.94)	47.53	105.24	47 (49)
Comoros	0.455	666.57 (393.82 to 1026.96)	95.21	571.37	163 (166)
Congo	0.568	984.8 (633.52 to 1307.59)	68.27	916.53	193 (191)
Cook Islands	0.764	1356.17 (1110.65 to 1654.83)	27.75	1328.42	204 (203)
Costa Rica	0.68	166.14 (131.09 to 211.64)	28.01	138.12	57 (53)
Coted'Ivoire	0.408	419.3 (233.17 to 606.99)	116.04	303.25	107 (113)
Croatia	0.794	247 (178.2 to 309.88)	26.96	220.04	85 (75)
Cuba	0.668	209.34 (113.94 to 263.45)	27.85	181.49	71 (65)
Cyprus	0.841	129.05 (73.77 to 152.05)	26.93	102.12	46 (42)
Czechia	0.828	119.75 (88.5 to 149.32)	26.96	92.8	40 (36)
Democratic People's Republic of Korea	0.558	376.14 (293.91 to 479.9)	69.76	306.38	108 (102)
Democratic Republic of the Congo	0.382	1007.4	120.33	887.07	192 (193)

		(626.95 to 1419.49)			
Denmark	0.89	30.64<br>(26.3 to 39.4)	26.87	3.77	3 (3)
Djibouti	0.459	650.37<br>(384.68 to 977.28)	94.82	555.55	162 (159)
Dominica	0.729	817.53<br>(667.38 to 1013.08)	27.91	789.62	184 (179)
Dominican Republic	0.592	401.7<br>(293.21 to 536.54)	60.64	341.06	116 (109)
Ecuador	0.64	318.89<br>(248.52 to 392.98)	49.52	269.36	97 (93)
Egypt	0.658	827.6<br>(337.69 to 1419.8)	39.48	788.12	183 (181)
El Salvador	0.573	109.33<br>(83.99 to 139.65)	67.54	41.79	22 (33)
Equatorial Guinea	0.685	672.72<br>(387.75 to 958.55)	27.83	644.89	170 (168)
Eritrea	0.396	998.01<br>(550.78 to 1610.23)	122.77	875.24	190 (192)
Estonia	0.835	953.31<br>(273.93 to 1252.67)	26.87	926.43	195 (190)
Eswatini	0.577	940.13<br>(601.41 to 1378.5)	68.52	871.61	189 (189)
Ethiopia	0.343	559.74<br>(322.52 to 920.44)	180.42	379.32	122 (145)
Fiji	0.664	633.91	29.78	604.13	165 (158)

		(504.63 to 797.76)			
Finland	0.856	138.12 (71.9 to 158.78)	26.81	111.31	48 (44)
France	0.834	48.68 (38.64 to 58.56)	26.86	21.81	16 (14)
Gabon	0.656	867.56 (555.11 to 1137.8)	44.53	823.03	186 (184)
Gambia	0.399	485.58 (268.43 to 698.93)	127.59	358	118 (133)
Georgia	0.702	860.06 (464.97 to 1036.65)	27.03	833.03	188 (183)
Germany	0.898	166.16 (141.24 to 205.81)	26.94	139.22	58 (54)
Ghana	0.557	388.63 (230.89 to 510.76)	69.68	318.96	111 (107)
Greece	0.794	116.97 (90.39 to 132.37)	26.96	90.02	38 (34)
Greenland	0.761	156.11 (123.84 to 193.8)	26.93	129.18	56 (52)
Grenada	0.669	491.13 (394.79 to 564.82)	27.86	463.27	145 (135)
Guam	0.813	417.68 (340.05 to 516.46)	26.68	390.99	124 (112)
Guatemala	0.526	117.32 (91.97 to 170.18)	87.78	29.53	18 (35)
Guinea	0.325	526.92	188.2	338.71	114 (141)

		(274.78 to 729.06)			
Guinea-Bissau	0.355	656.26 (308.11 to 953.45)	145.4	510.86	152 (163)
Guyana	0.618	1157.4 (894.93 to 1490.03)	51.23	1106.18	200 (199)
Haiti	0.432	762.51 (450.22 to 1180.99)	110.78	651.72	171 (175)
Honduras	0.496	285.5 (192.55 to 464.2)	94.84	190.66	74 (84)
Hungary	0.791	436.04 (302.49 to 538.33)	26.93	409.11	128 (117)
Iceland	0.869	44.08 (35.34 to 52.11)	26.86	17.21	13 (12)
India	0.566	211.89 (143.33 to 281.79)	69.48	142.41	60 (67)
Indonesia	0.66	575.83 (334.78 to 693.74)	40.5	535.33	159 (151)
Iran (Islamic Republic of)	0.67	498.63 (436.88 to 540.58)	27.22	471.41	146 (138)
Iraq	0.671	314.7 (251.47 to 376.43)	27.12	287.58	102 (92)
Ireland	0.867	35.23 (29.23 to 48)	26.93	8.3	6 (6)
Israel	0.803	27.79 (21.78 to 63.59)	26.92	0.87	1 (1)
Italy	0.801	173.12	26.94	146.18	61 (56)

		(111.43 to 195.46)			
Jamaica	0.684	451.09 (357.44 to 594.82)	28.11	422.98	132 (123)
Japan	0.87	34.71 (28.13 to 56.14)	26.93	7.77	5 (5)
Jordan	0.731	664.81 (440.78 to 797.98)	27.8	637.01	169 (165)
Kazakhstan	0.723	275.43 (217.9 to 531.29)	27.83	247.6	91 (83)
Kenya	0.508	564.71 (343.25 to 903.33)	88.42	476.29	148 (148)
Kiribati	0.527	482.06 (360.83 to 603.18)	87.53	394.53	126 (132)
Kuwait	0.851	371.52 (302.91 to 490.8)	26.83	344.69	117 (100)
Kyrgyzstan	0.596	252.2 (193.65 to 299.66)	57.23	194.97	78 (77)
Lao People's Democratic Republic	0.49	530.96 (291.98 to 744.47)	94.5	436.46	135 (142)
Latvia	0.82	247.7 (84.17 to 321.63)	26.97	220.74	86 (76)
Lebanon	0.708	462.83 (205.46 to 615.98)	27.85	434.98	134 (126)
Lesotho	0.507	1155.48 (776.43 to 1565.67)	94.54	1060.95	197 (198)
Liberia	0.37	431.52	129.08	302.44	106 (115)

		(229.83 to 661.58)			
Libya	0.709	470.77<br>(222.8 to 681.05)	27.81	442.97	140 (130)
Lithuania	0.843	166.29<br>(94.36 to 212.57)	26.9	139.38	59 (55)
Luxembourg	0.895	63.47<br>(50.71 to 80.7)	26.84	36.62	21 (18)
Madagascar	0.396	1258.83<br>(740.06 to 1886.4)	118.82	1140.01	201 (201)
Malawi	0.384	653.02<br>(377.99 to 1037.4)	126.38	526.64	156 (162)
Malaysia	0.737	57.32<br>(45.09 to 72.34)	27.76	29.56	19 (16)
Maldives	0.562	122.37<br>(94.51 to 147.26)	68.29	54.08	30 (38)
Mali	0.263	450.36<br>(262.64 to 600.53)	241.54	208.83	82 (122)
Malta	0.801	74.16<br>(61.31 to 93.86)	26.9	47.26	26 (23)
Marshall Islands	0.544	903.76<br>(537.95 to 1248.97)	76.23	827.53	187 (187)
Mauritania	0.496	334.95<br>(206.95 to 454.02)	94.9	240.05	89 (97)
Mauritius	0.705	476.36<br>(374.26 to 590.37)	27.33	449.04	143 (131)
Mexico	0.649	136.59	46.42	90.17	39 (43)

		(113 to 160.35)			
Micronesia (Federated States of)	0.58	938.53 (526.8 to 1304.25)	62.33	876.2	191 (188)
Monaco	0.902	77.45 (61.32 to 94.18)	26.91	50.55	27 (25)
Mongolia	0.606	211.03 (161.85 to 267.86)	56.35	154.68	64 (66)
Montenegro	0.791	81.31 (65.13 to 99.46)	26.88	54.42	31 (27)
Morocco	0.548	682.95 (305.88 to 920.55)	71.28	611.67	166 (169)
Mozambique	0.307	899.39 (543.08 to 1336.29)	203.82	695.58	176 (186)
Myanmar	0.521	451.29 (271 to 593.93)	91.48	359.81	121 (124)
Namibia	0.612	713.11 (488.03 to 1002.43)	51.7	661.41	173 (170)
Nauru	0.618	758.28 (435.46 to 1033.62)	52.56	705.73	179 (174)
Nepal	0.422	232.62 (166.91 to 318.96)	108.08	124.54	54 (71)
Netherlands	0.883	35.87 (29.04 to 41.42)	26.96	8.92	7 (7)
New Zealand	0.84	41.87 (35.89 to 56.08)	26.95	14.92	11 (11)
Nicaragua	0.517	286.03	94.19	191.85	76 (85)

		(230.78 to 334.71)			
Niger	0.162	374.87<br>(214.16 to 559.11)	358.76	16.1	12 (101)
Nigeria	0.515	334.15<br>(214.84 to 445.2)	92.88	241.27	90 (96)
Niue	0.711	467.67<br>(297.45 to 637.15)	27.34	440.33	137 (128)
North Macedonia	0.744	560.97<br>(441.04 to 673.26)	27.91	533.06	157 (146)
Northern Mariana Islands	0.771	151.63<br>(120.32 to 181.7)	27.85	123.78	52 (48)
Norway	0.913	27.98<br>(23.51 to 42.67)	26.95	1.03	2 (2)
Oman	0.783	385.48<br>(296.43 to 461.08)	27.38	358.1	119 (105)
Pakistan	0.449	370.71<br>(246.9 to 481.88)	94.55	276.16	99 (99)
Palau	0.738	70.26<br>(54.53 to 88.52)	26.91	43.35	23 (20)
Palestine	0.588	587.37<br>(410.37 to 686.47)	62.04	525.33	155 (154)
Panama	0.686	125.63<br>(93.94 to 159.52)	26.94	98.69	43 (40)
Papua New Guinea	0.394	633.25<br>(381.1 to 910.88)	121.89	511.37	153 (157)
Paraguay	0.638	265.6	48.6	217	84 (80)

		(200.16 to 340.5)			
Peru	0.648	67.34<br>(50.85 to 90.01)	49.24	18.1	14 (19)
Philippines	0.623	726.41<br>(513.94 to 866.68)	50.49	675.92	175 (173)
Poland	0.802	189.41<br>(147.2 to 229.89)	26.89	162.52	67 (62)
Portugal	0.743	72.68<br>(51.59 to 83.3)	27.85	44.83	25 (22)
Puerto Rico	0.814	229.48<br>(168.41 to 294.66)	26.96	202.52	79 (70)
Qatar	0.83	87.11<br>(64.4 to 114.29)	26.86	60.26	32 (28)
Republic of Korea	0.878	77.44<br>(62.77 to 111.45)	26.81	50.62	28 (24)
Republic of Moldova	0.696	432.7<br>(144.18 to 534.95)	27.32	405.38	127 (116)
Romania	0.76	469.84<br>(369.82 to 573.02)	26.85	443	141 (129)
Russian Federation	0.805	149.68<br>(102.69 to 176.46)	26.85	122.83	51 (47)
Rwanda	0.429	591.48<br>(185.06 to 1040.01)	112.18	479.3	149 (155)
Saint Kitts and Nevis	0.746	466.34<br>(371.54 to 571.69)	27.81	438.53	136 (127)
Saint Lucia	0.67	420.41	27.82	392.59	125 (114)

		(346.47 to 511.47)			
Saint Vincent and the Grenadines	0.627	670.46 (557.4 to 788.31)	50.87	619.59	167 (167)
Samoa	0.641	582.33 (381.97 to 767.2)	47.48	534.85	158 (152)
San Marino	0.884	91.85 (64.94 to 126.94)	26.97	64.88	33 (29)
Sao Tome and Principe	0.502	183.62 (129.68 to 237.41)	94.81	88.81	37 (59)
Saudi Arabia	0.805	95.68 (73.26 to 120.81)	26.93	68.75	35 (31)
Senegal	0.389	380.38 (222.33 to 521.67)	122.09	258.29	93 (103)
Serbia	0.767	290.03 (222.33 to 351.34)	27.74	262.29	94 (86)
Seychelles	0.724	806.86 (698.8 to 938.81)	28.4	778.47	182 (178)
Sierra Leone	0.347	384.41 (221.7 to 546)	178.69	205.71	81 (104)
Singapore	0.861	184.86 (119.01 to 212.41)	26.94	157.92	65 (60)
Slovakia	0.812	207.29 (147.71 to 260.55)	26.95	180.34	70 (64)
Slovenia	0.84	202.68 (120.25 to 262.43)	26.87	175.81	69 (63)
Solomon Islands	0.407	820.85	120.67	700.17	177 (180)

		(462.86 to 1209.22)			
Somalia	0.081	1012.32 (619.26 to 1508.15)	358.05	654.27	172 (194)
South Africa	0.678	574.44 (504.77 to 647.03)	26.99	547.46	161 (150)
South Sudan	0.363	573.81 (335.55 to 989.69)	132.6	441.21	138 (149)
Spain	0.767	72.51 (46.42 to 84.13)	27.79	44.72	24 (21)
Sri Lanka	0.69	311.63 (222.3 to 410.09)	27.97	283.65	100 (90)
Sudan	0.515	845.63 (353.78 to 1262.77)	93.66	751.96	180 (182)
Suriname	0.636	437.04 (351.48 to 530.31)	46.96	390.08	123 (118)
Sweden	0.872	78.62 (40.71 to 92.15)	26.83	51.78	29 (26)
Switzerland	0.929	94.86 (50.46 to 111.45)	26.96	67.89	34 (30)
Syrian Arab Republic	0.619	152.79 (117.93 to 200.31)	52.86	99.93	45 (50)
Taiwan (Province of China)	0.868	179.69 (143.55 to 241.43)	26.97	152.72	63 (58)
Tajikistan	0.539	873.59 (556.44 to 1085.37)	68.63	804.96	185 (185)
Thailand	0.687	47.91	27.82	20.08	15 (13)

		(36.74 to 61.63)			
Timor-Leste	0.514	536.28 (313.75 to 791.27)	94.3	441.98	139 (143)
Togo	0.417	440.13 (256.17 to 600.18)	110.56	329.57	112 (120)
Tokelau	0.626	496.3 (328.52 to 678.01)	50.37	445.94	142 (136)
Tonga	0.636	173.78 (130.86 to 224.72)	49.62	124.17	53 (57)
Trinidad and Tobago	0.757	323.2 (236.84 to 539.1)	27.48	295.71	103 (94)
Tunisia	0.672	443.5 (205.22 to 622.25)	27.93	415.57	130 (121)
Turkey	0.748	292.69 (204.44 to 360.56)	27.78	264.91	96 (88)
Turkmenistan	0.67	324.69 (250.49 to 415.84)	28.3	296.4	104 (95)
Tuvalu	0.589	660.1 (416.98 to 917.22)	59.77	600.32	164 (164)
Uganda	0.404	583.23 (222.53 to 995.65)	107.38	475.85	147 (153)
Ukraine	0.736	31.93 (26.22 to 44.89)	27.05	4.88	4 (4)
United Arab Emirates	0.88	802.29 (297.63 to 1587.53)	26.96	775.34	181 (177)
United Kingdom	0.847	60.68	26.82	33.86	20 (17)

		(54.28 to 73.98)			
United Republic of Tanzania	0.423	652.29 (339.01 to 1044.29)	113.96	538.34	160 (161)
United States of America	0.859	212.19 (138.06 to 230.45)	26.97	185.22	73 (68)
United States Virgin Islands	0.799	489.06 (407.76 to 574.98)	26.94	462.11	144 (134)
Uruguay	0.697	149.27 (111.94 to 169.58)	27.35	121.91	50 (46)
Uzbekistan	0.631	462.25 (311.79 to 622.59)	50.79	411.46	129 (125)
Vanuatu	0.485	718.68 (429.99 to 994.74)	94.63	624.05	168 (171)
Venezuela (Bolivarian Republic of)	0.607	350.82 (263.93 to 486.71)	53.33	297.49	105 (98)
Viet Nam	0.617	385.97 (235.52 to 554.07)	51.29	334.68	113 (106)
Yemen	0.412	1024.08 (426.76 to 1623.17)	102.41	921.67	194 (195)
Zambia	0.505	1034.13 (635.33 to 1388.76)	94.84	939.29	196 (196)
Zimbabwe	0.476	407.47 (280.17 to 675.29)	94.57	312.9	109 (110)

DALYs: disability-adjusted life years; SDI: socio-demographic index; UI: uncertainty interval.
